# Supplementary material for: PEP4LEP study protocol: integrated skin screening and SDR-PEP administration for leprosy prevention: comparing the effectiveness and feasibility of a community-based intervention to a health centre-based intervention in Ethiopia, Mozambique and Tanzania
Source: BMJ Open. 2021 Aug 26;11(8):e046125. doi: 10.1136/bmjopen-2020-046125 (PMC8395349; doi:10.1136/bmjopen-2020-046125)
Supplement: Author's manuscript [file bmjopen-2020-046125.draft_revisions.pdf]

BMJ Open

BMJ Open is committed to open peer review. As part of this commitment we make the peer review history of every article we publish publicly available.

When an article is published we post the peer reviewers' comments and the authors' responses online. We also post the versions of the paper that were used during peer review. These are the versions that the peer review comments apply to.

The versions of the paper that follow are the versions that were submitted during the peer review process. They are not the versions of record or the final published versions. They should not be cited or distributed as the published version of this manuscript.

BMJ Open is an open access journal and the full, final, typeset and author-corrected version of record of the manuscript is available on our site with no access controls, subscription charges or pay-per-view fees (http://bmjopen.bmj.com).

If you have any questions on BMJ Open's open peer review process please email info.bmjopen@bmj.com

BMJ Open

The PEP4LEP study protocol: Integrated skin screening and SDR-PEP administration for leprosy prevention. Comparing the effectiveness and feasibility of a community-based intervention to a health center-based intervention in Ethiopia, Mozambique and Tanzania

Journal:	*BMJ Open*
Manuscript ID	bmjopen-2020-046125
Article Type:	Protocol
Date Submitted by the Author:	20-Oct-2020
Complete List of Authors:	Schoenmakers, Anne; NLR<br>Hambridge, Thomas; Erasmus Medical Center<br>van Wijk, Robin; NLR<br>Kasang, Christa; DAHW<br>Richardus, Jan Hendrik; Erasmus Medical Center, Department of Public Health<br>Bobosha, Kidist; Armauer Hansen Research Institute<br>Mitano, Fernando; Lurio University<br>Mshana, Stephen E.; Catholic University of Health and Allied Sciences, Department of Microbiology and Immunology<br>Mamo, Ephrem; Armauer Hansen Research Institute<br>Marega, Abdoulaye; Lurio University<br>Mwageni, Nelly; Catholic University of Health and Allied Sciences, Department of Microbiology and Immunology<br>Letta, Taye; Ethiopia Ministry of Health<br>Muloliwa, Artur; Ministry of Health, Mozambique<br>Kamara, Vedastus; Ministry of Health, Tanzania<br>Eman, Ahmed; DAHW, Ethiopia<br>Raimundo, Litos; NLR, Mozambique<br>Njako, Blasdus; DAHW, Tanzania<br>Mieras, Liesbeth; NLR
Keywords:	Public health < INFECTIOUS DISEASES, Infectious diseases & infestations < DERMATOLOGY, Tropical medicine < INFECTIOUS DISEASES

SCHOLARONE™
Manuscripts

I, the Submitting Author has the right to grant and does grant on behalf of all authors of the Work (as defined in the below author licence), an exclusive licence and/or a non-exclusive licence for contributions from authors who are: i) UK Crown employees; ii) where BMJ has agreed a CC-BY licence shall apply, and/or iii) in accordance with the terms applicable for US Federal Government officers or employees acting as part of their official duties; on a worldwide, perpetual, irrevocable, royalty-free basis to BMJ Publishing Group Ltd ("BMJ") its licensees and where the relevant Journal is co-owned by BMJ to the co-owners of the Journal, to publish the Work in this journal and any other BMJ products and to exploit all rights, as set out in our licence.

The Submitting Author accepts and understands that any supply made under these terms is made by BMJ to the Submitting Author unless you are acting as an employee on behalf of your employer or a postgraduate student of an affiliated institution which is paying any applicable article publishing charge ("APC") for Open Access articles. Where the Submitting Author wishes to make the Work available on an Open Access basis (and intends to pay the relevant APC), the terms of reuse of such Open Access shall be governed by a Creative Commons licence – details of these licences and which Creative Commons licence will apply to this Work are set out in our licence referred to above.

Other than as permitted in any relevant BMJ Author's Self Archiving Policies, I confirm this Work has not been accepted for publication elsewhere, is not being considered for publication elsewhere and does not duplicate material already published. I confirm all authors consent to publication of this Work and authorise the granting of this licence.

For peer review only

The PEP4LEP study protocol: Integrated skin screening and SDR-PEP administration for leprosy prevention. Comparing the effectiveness and feasibility of a community-based intervention to a health center-based intervention in Ethiopia, Mozambique and Tanzania

A. Schoenmakers[1*], T. Hambridge[2*], R. van Wijk[1], C. Kasang[3], J.H. Richardus[2], K. Bobosha[4], F. Mitano[5], S.E. Mshana[6], E. Mamo[4], A. Marega[5], N. Mwageni[6], Taye Letta[7], A. M. Muloliwa[8], D. V. Kamara[9], A. M. Eman[10], L. Raimundo[11], B. Njako[12], L. Mieras[1]

[1] NLR, The Netherlands; [2] Erasmus MC, University Medical Center Rotterdam, The Netherlands; [3] DAHW, Germany; [4] AHRI, Ethiopia; [5] Universidade Lúrio, Mozambique; [6] CUHAS University, Tanzania, [7] Ministry of Health, Ethiopia; [8] Ministry of Health, Mozambique; [9] Ministry of Health, Tanzania; [10] GLRA, Ethiopia; [11] NLR, Mozambique, [12] GLRA, Tanzania

Both authors contributed equally to this article.

Correspondence: t.hambridge@erasmusmc.nl

For peer review only

Abstract

Introduction

Leprosy, or Hansen's disease, remains a cause of preventable disability. Early detection, treatment and prevention are key to reduce *Mycobacterium leprae* transmission. Post-exposure prophylaxis with single-dose rifampicin (SDR-PEP) reduces the risk of developing leprosy when administered to screened contacts of patients. This has been adopted in the World Health Organization (WHO) guidelines on leprosy. The PEP4LEP study aims to determine the most effective and feasible method of screening people at risk of developing leprosy and administering chemoprophylaxis to contribute to interrupting transmission.

Methods and analysis

PEP4LEP is a cluster-randomized implementation trial comparing two interventions of integrated skin screening combined with SDR-PEP distribution to contacts of leprosy patients in Ethiopia, Mozambique, and Tanzania. One intervention is community-based, using skin camps to screen approximately 100 community contacts per leprosy patient and to administer SDR-PEP to eligible contacts. The other intervention is health center-based, inviting household contacts of leprosy patients to be screened in a local health center and subsequently receive SDR-PEP when eligible. The mobile health (mHealth) tool SkinApp will support health workers' capacity in integrated skin screening. The effectiveness of both interventions will be compared by assessing

the rate of leprosy patients detected and the period of case detection delay, as well as feasibility in terms of cost-effectiveness and acceptability.

Ethics and dissemination

Ethical approval has been obtained in the project countries. Results from this study will be published open access in peer-reviewed journals and provide evidence for the implementation of novel leprosy screening methods and chemoprophylaxis to policymakers.

Trial registration: The PEP4LEP project is registered at the Netherlands Trial Register (NTR), receiving trial registration number NL7294 (NTR7503), registration date September 10, 2018.

Keywords: leprosy, Hansen's disease, NTD, chemoprophylaxis, prevention, skin screening, case detection, single dose rifampicin, SDR-PEP, post-exposure prophylaxis, detection delay, skin camps, Ethiopia, Mozambique, Tanzania, Africa, feasibility, acceptability, cost-effectiveness, mHealth, eHealth

Article Summary

Strengths and Limitations

- In both interventions, newly diagnosed patients can be screened and treated for leprosy and other skin diseases / skin NTDs, while SDR-PEP will be administered – according to the World Health Organization's guidelines – to eligible contacts of leprosy patients to reduce their risk to develop leprosy
- An integrated skin diseases approach will be used in which multiple diseases can be detected and treated at once, which may also overcome the frequently negative associations with leprosy that can prevent people from participating in leprosy-related interventions; the included leprosy patients do not need to share their disease status with their contacts in the community (skin camp) intervention arm
- The SkinApp will be used as a mHealth tool to support peripheral health workers in recognizing and treating signs and symptoms of skin diseases
- Because of the long incubation period of leprosy as well as the delays in case detection, the epidemiological impact of this study on the new case detection rate will not become apparent within the study duration of four years

For peer review only - http://bmjopen.bmj.com/site/about/guidelines.xhtml

- As difficulties in recalling the first signs and symptoms are expected to increase over a longer duration of the disease, only recently diagnosed index patients will be included in this study to establish case detection delay

Introduction

Leprosy, or Hansen's disease, is a communicable disease caused by *Mycobacterium leprae* that is still a public health problem in many countries. It is formally recognized by the World Health Organization (WHO) as a neglected tropical disease (NTD).[1] The annual reported number of newly detected leprosy patients was 208,613 in 2018.[2] If left untreated, leprosy potentially results in disability, which can have severe consequences such as stigma and poverty.[3] Leprosy has a long and variable incubation time, ranging from 2 to 20 years, during which it is assumed that transmission can take place.[4] The risk of developing leprosy is higher in household contacts and neighbors of patients than it is in the general community.[5] Moet et al. demonstrated that physical and genetic distance were independently associated with the risk of a contact developing leprosy.[6]

The WHO provides multidrug therapy (MDT) free of charge to all leprosy patients since 1995.[7] However, to overcome ongoing transmission in high-endemic areas, innovative measures are needed. In 2008, a large randomized controlled trial in Bangladesh (Chemoprophylaxis of Leprosy study, COLEP) demonstrated that a single dose of rifampicin (SDR) given to contacts of newly diagnosed leprosy patients is effective in reducing the risk of leprosy by 57% (95% CI: 24–75%).[8] SDR-PEP was found to be cost-effective in Bangladesh.[9] In the Leprosy Post-Exposure Prophylaxis (LPEP) program, SDR-PEP was implemented in areas representing various health systems across three continents and eight countries, to evaluate the feasibility, effectiveness and impact (Richardus, et al. under publication).[10] The implementation of SDR-PEP within the routine leprosy control programs was proven to be safe and generally well accepted.[11] Based on the LPEP program and a microsimulation leprosy model (SIMCOLEP), SDR-PEP was also found to be cost-effective in India.[12] The concern that SDR-PEP could lead to increased rifampicin resistance in other diseases, such as tuberculosis (TB), was considered in an expert consultation that concluded that SDR-PEP given to contacts of leprosy patients, in the absence of symptoms of active TB, poses a negligible risk of generating resistance in *Mycobacterium tuberculosis* in individuals and in populations.[13]

Skin screening is an important detection strategy for skin-NTDs like leprosy.[1,14,15] Screening for multiple skin diseases at once (integrated or common skin screening) is promoted by WHO.[1,16,17] Integration is considered to increase effectiveness and efficiency by minimizing costs and

For peer review only - http://bmjopen.bmj.com/site/about/guidelines.xhtml

expanding intervention coverage.[16,18] An important obstacle for integrated skin screening is the scarcity of dermatologists in many areas with a high skin NTD endemicity.[19] In sub-Saharan Africa, the situation is critical, with approximately 1 dermatologist per 500,000–1 million inhabitants and even larger shortages in Mozambique and Tanzania according to field reports from PEP4LEP consortium members.[20,21] According to the WHO, community health workers (CHWs) and village volunteers can play a role in screening for skin diseases, but improved knowledge, capacity, and motivation of health workers and community volunteers is essential.[14,16,22–26]

As both integrated skin screening for NTDs and SDR-PEP against leprosy are promoted by the WHO, additional implementation studies are necessary to establish whether a combined intervention is acceptable, feasible, and cost-effective in leprosy endemic areas.[1,4,16]

Objectives

The PEP4LEP project is a cross-functional collaboration among study consortium members in five countries in sub-Saharan Africa and the European Union (EU) (Figure 1). The overall aim of this cluster-randomized implementation trial is to contribute to interrupt the transmission of *M. leprae* by identifying the most effective and feasible method of screening people at risk of developing leprosy and by administering post-exposure chemoprophylaxis in Ethiopia, Mozambique, and Tanzania. The primary study objectives are to compare the effectiveness and feasibility of a community-based screening and prophylaxis (skin camp) intervention with a health center-based screening and prophylaxis intervention solely for household contacts of a leprosy patient. The case detection delay will be the primary outcome measure to assess effectiveness. Additional objectives are to assess the cost-effectiveness, acceptability and health workers' capacity regarding the integrated skin diseases approach and the use of the supportive mobile health (mHealth) tool SkinApp.[27,28]

For peer review only - http://bmjopen.bmj.com/site/about/guidelines.xhtml

For peer review only

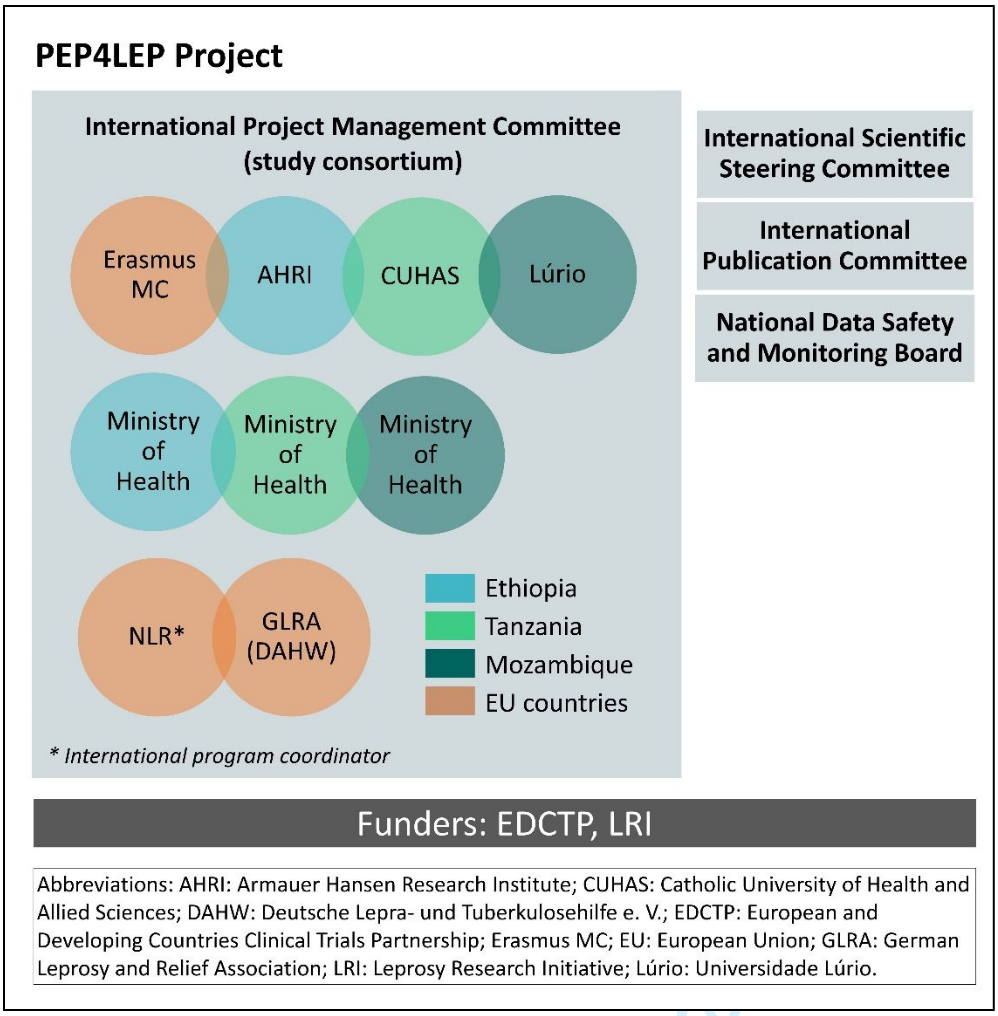

Figure 1. PEP4LEP Project organization chart

Methods and analysis

<u>Study setting</u>

This study will take place in three countries in sub-Saharan Africa: Ethiopia, Mozambique, and Tanzania. The three countries differ socioculturally and in the endemicity for NTDs like leprosy (Figure 2).[29] Districts within these countries were purposefully chosen because of the high distribution of reported leprosy cases. In Ethiopia, three endemic districts are located in East Hararghe Zone (Oromiya region): Girawa, Jarso, and Midega. In Mozambique, the included districts are located in Nampula province: Meconta, Mogovolas, and Murrupula. The Tanzanian districts are Lindi in Lindi Region and Morogoro and Mvomero in Morogoro Region. The original overall study period was October 2018 until January 2023, with an estimated duration of 2.5–3 years for the inclusion of leprosy patients and their contacts.

For peer review only - http://bmjopen.bmj.com/site/about/guidelines.xhtml

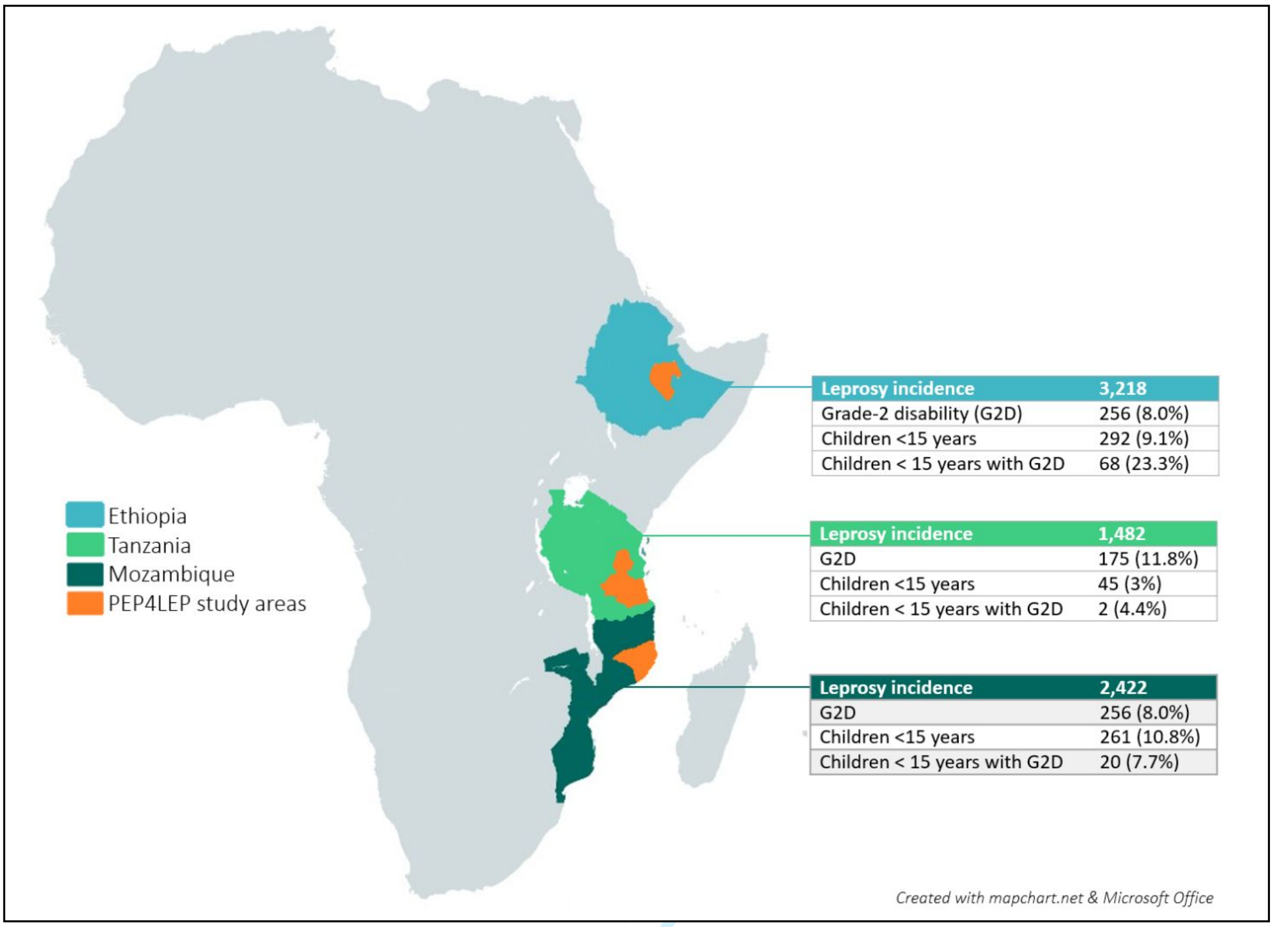

Figure 2. PEP4LEP countries' leprosy incidence (2018) according to the World Health Organization[29]

Participants and eligibility criteria

New leprosy patients enrolled in the PEP4LEP study are referred to as "index patients". The inclusion and exclusion criteria for index patients and contacts are summarized in Table 1 and are based on the WHO guidelines and the LPEP program.[11,30] Following the emergence of the Coronavirus Disease 2019 (COVID-19) pandemic, a suspicion of a COVID-19 infection was added as contact exclusion criteria, as physical distancing cannot be guarded when performing skin screening.[31–34] Index patients with suspected COVID-19 can still be included after they have been tested negative and are symptom-free for at least 2 weeks.[31–33] Information, Education and Communication (IEC)-materials will be designed to inform potential study participants.[32]

Table 1. PEP4LEP eligibility criteria[4,10]

For peer review only - http://bmjopen.bmj.com/site/about/guidelines.xhtml

	Index patients	Contacts
**Inclusion criteria**	• Consent to participate in the PEP4LEP project<br>• Diagnosed with leprosy (preferred maximum of 6 months prior to inclusion)<br>• Residence in the PEP4LEP districts for ≥3 months prior to the date of diagnosis<br>• Index patient has started MDT<br>• Community-based skin camp intervention: Leprosy patient gives permission for the set-up of a skin camp in his/her community (sharing their leprosy diagnosis with their contacts is not needed)<br>• Health center-based household screening intervention: Leprosy patient with household contacts, and who is willing to inform these contacts about PEP4LEP	• Consent to participate in the PEP4LEP project<br>• Community-based skin camp intervention: Community contact of the index patient for ≥3 months<br>• Health center-based household screening intervention: Contact which is a household member of the index patient for ≥3 months, visiting the screening health center ≤3 months after the index patient was included
**Exclusion criteria**	• Index patient or parents/legal guardians unable to understand the purpose and risks of participating in the PEP4LEP study	• Contact or parents/legal guardians unable to understand the purpose and risks of participating in the PEP4LEP study<br>• Age <2 years and/or <10 kg of weight*<br>• Pregnancy*<br>• Receiving or having received rifampicin for any reason in the last 2 years<br>• Known allergy to rifampicin<br>• History of liver or renal disorders<br>• Individuals with leprosy and those who have possible signs and/or symptoms of leprosy (e.g., leprosy-like skin lesions or nerve manifestations) until their disease status has been clarified[35]**

For peer review only - http://bmjopen.bmj.com/site/about/guidelines.xhtml

		• Individuals with possible signs and/or symptoms of TB (cough for more than two weeks or cough in known HIV/AIDS patients, night sweats, unexplained fever, weight loss) until their disease status has been clarified[35]*** • Individuals with possible signs and/or symptoms of COVID-19 (self-assessed temperature ≥38°C, respiratory or cold-like symptoms, sudden loss of smell/taste) or possible contact with a COVID-19 patient in the past 14 days.[31–34]***
* A voucher will be given for repeated skin screening and SDR-PEP. This can be used in a PEP4LEP affiliated health center when this person becomes eligible (e.g., after giving birth).<br>** If referral was needed and no leprosy is detected, repeated skin screening and SDR-PEP can be provided in a PEP4LEP affiliated health center.<br>*** Skin screening and SDR-PEP can only be provided in a PEP4LEP affiliated health center after the contact is tested negative for COVID-19/TB (according to national guidelines).[31–34]		
Abbreviations: COVID-19: Coronavirus Disease 2019; MDT: multidrug therapy; SDR-PEP: single-dose rifampicin post-exposure prophylaxis; TB: tuberculosis		

<u>Study design</u>

The study is a two-arm, cluster-randomized implementation trial (Figure 3). One intervention is community-based, using skin camps to screen approximately 100 community contacts (household members and neighbors) of a leprosy index patient and to provide them with SDR-PEP when eligible. The second intervention is health center-based, inviting the household contacts of an index patient to be screened and given SDR-PEP when eligible.

<u>Community-based skin camp intervention</u>

A skin camp will be organized when a leprosy patient is diagnosed by inviting approximately 100 people living in the surrounding area (or inhabitants from the 20 closest houses). Health camps are designed to bring specialized care closer to the community, thus expanding access.[36] Besides preventive and curative treatment, these camps often also play a significant role to create awareness.[37] Community "skin health camps" have been proposed as an effective way to screen for leprosy and other NTDs.[38] Skin camps are organized at the community level and in

For peer review only - http://bmjopen.bmj.com/site/about/guidelines.xhtml

close collaboration with community leaders and local organizations.[36,39] In a skin camp, health staff screen individuals for skin diseases and then treat or refer patients if necessary. Assistance from a dermatologist (or, if none available, a senior health staff member with sufficient dermatology experience) is vital.[40] A key advantage of this community intervention is that the identity of the person affected by leprosy can be protected since no individual disease disclosure is needed. This non-disclosure approach is of utmost importance, as people affected by leprosy are often stigmatized and discriminated against and are therefore reluctant to share their disease status.[41–43] Moreover, including a wider group of contacts and using an integrated skin diseases approach may overcome the frequently negative associations with leprosy that can prevent people from participating in a leprosy-related intervention.[16] Including approximately 100 contacts per identified leprosy patient in the PEP4LEP skin camps is in line with the risk profiles of the contact groups and is operationally manageable within one skin camp day, also when using time slots to prevent crowding considering COVID-19.[6,32,34,37,38,44–47]

Health center-based intervention for household contacts

In the second intervention, newly detected leprosy patients will be asked to invite their household contacts to visit a health center to have their skin screened and, if eligible, to be offered SDR-PEP. Clustering of the disease within households is commonly seen.[6,47,48] Household contacts are defined as living under the same roof as the leprosy index patient for a minimum of three months.[11,30,49] To prevent re-infection within a household and for operational management reasons, contacts need to visit the health center within three months after the index patient was included, which is also in-line with contact tracing interventions in literature.[50] Around six household contacts per patient are expected to visit the health center for screening.[11] Previous studies showed that leprosy patients are usually willing to disclose their leprosy diagnosis to their household members to facilitate screening and prophylaxis, but they are often reluctant to share this information with neighbors or other social contacts.[41–43]

For peer review only - http://bmjopen.bmj.com/site/about/guidelines.xhtml

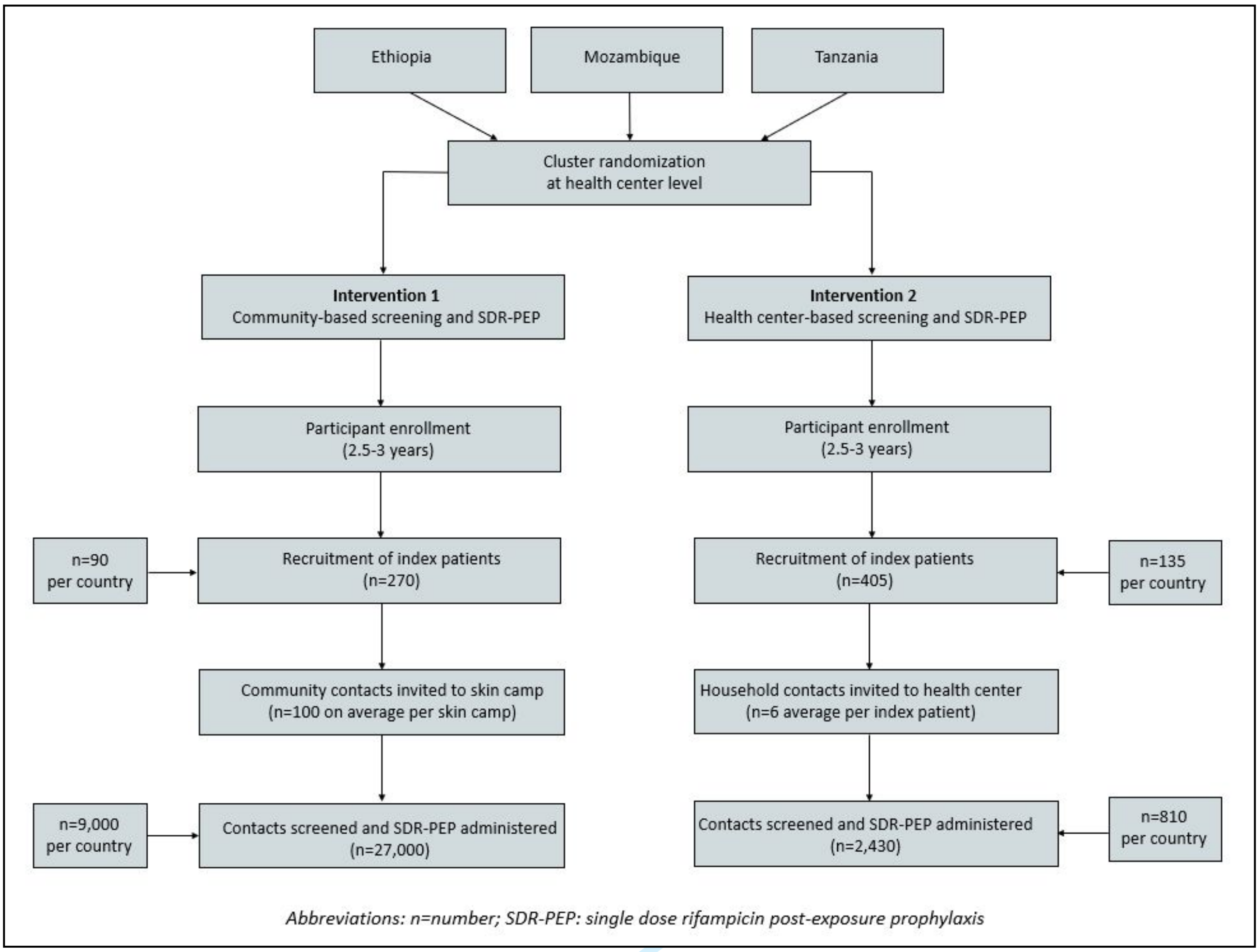

Figure 3. Flow of participants through the PEP4LEP study

<u>Integrated skin screening</u>

For contact screening in both interventions, an integrated skin diseases approach - also called common skin screening approach - will be used to diagnose common skin diseases (e.g., eczema), skin conditions related to HIV/AIDS (e.g., Kaposi's sarcoma), and skin-NTDs (e.g., leprosy). "Integration" in this context refers to combined screening for a minimum of two diseases at the same time in the same communities.[51] In the PEP4LEP project, free topical treatment for the most frequently diagnosed skin diseases will be provided as well as referral advice, in-line with national medical guidelines. The screening for signs and symptoms of skin diseases, as well as the chemoprophylaxis distribution, will follow standard operating procedures (SOPs) in which the eligibility criteria for SDR-PEP are clearly stated. In both interventions, the integrated skin diseases approach will be used and supported by the SkinApp, a mHealth tool developed by NLR and Erasmus University Medical Center (Erasmus MC). The SkinApp will support peripheral health workers in recognizing and treating signs and symptoms of skin diseases, including skin-NTDs like leprosy.[27,28] A senior health staff member with sufficient dermatology experience

For peer review only - http://bmjopen.bmj.com/site/about/guidelines.xhtml

(preferably a dermatologist) will attend in person or via secure medical messaging via the application (app) Siilo.[52]

Post-exposure prophylaxis

Chemoprophylaxis with SDR-PEP has been adopted in the 2018 WHO *Guidelines for the diagnosis, treatment and prevention of leprosy*.[4] The SDR-PEP dosages used in this project (Table 2) are consistent with the WHO guidelines and the LPEP program.[4,11,53]

For peer review only

Table 2. PEP4LEP single-dose rifampicin dosages[4,10]

Age and body weight of contact	Rifampicin dosage
≥15 years	600 mg
10-14 years	450 mg
6-9 years and body weight of ≥20 kg	300 mg
≥2 years old and body weight between 10-20 kg	150 mg

Contacts who are temporarily ineligible to receive SDR-PEP (e.g., because of pregnancy, Table 1) will receive skin screening and a SDR-PEP voucher, useable in an affiliated PEP4LEP health center when becoming eligible (e.g., after giving birth). Contacts receiving SDR-PEP will also receive a SDR-PEP Red Card to keep in their homes. This card indicates that the person has received SDR-PEP for leprosy prevention and is ineligible to receive this again within the next two years. These methods were previously used as part of the LPEP program in Tanzania.[11] In PEP4LEP, serious adverse events (SAEs) will be reported and followed up according to national and PEP4LEP guidelines (see ethical section).[54]

Outcomes

The primary objectives of this study are to identify the most effective and feasible approach for screening contacts of leprosy patients in combination with administering chemoprophylaxis to prevent leprosy. Because of the long incubation period of leprosy, it will not be possible to observe reduced transmission at the population level, in terms of a reduced new case detection rate, during this project period. The active case finding component and raised awareness, however, are expected to lead to more cases improved early case detection (i.e., a shorter case detection delay) and reduced child cases and disability at the time of diagnosis.

Primary outcome measures

The primary outcome measures of effectiveness in the comparison of the two interventions are:

1) Case detection delay, measured in months since the first signs or symptoms of leprosy until diagnosis and in the number of patients with G2D.
2) Number of new patients with leprosy, subdivided into child proportion, female proportion, and multibacillary (MB) / paucibacillary (PB) classification.
3) Number of contacts screened and receiving SDR-PEP.

Secondary outcome measures

For peer review only - http://bmjopen.bmj.com/site/about/guidelines.xhtml

Feasibility will be assessed by looking at outcome measures related to cost-effectiveness and acceptability. A cost-effectiveness analysis will be undertaken in the third year of the project. The perspective will be social, which encompasses the costs incurred by the health system and the beneficiaries (out-of-pocket expenditure). The acceptability of both interventions will be determined by comparing the number of index patients and contacts included, as well as by using qualitative research methods such as interviews and focus group discussions (FGDs) with relevant stakeholders.

Additional objectives

The additional objectives are to assess the acceptability of integrated skin screening and the use of the SkinApp as well as health workers' capacity regarding the integrated skin screening approach. This will be measured by the number of contacts diagnosed with skin diseases and NTDs and by observing use of the SkinApp during contact screening. The capacity of health workers to diagnose leprosy and other skin diseases will be determined by a series of four assessments: before (baseline) and after PEP4LEP training, during the study, at the end of the study. Additionally, qualitative methods including interviews, FGDs, and potentially observations will be used for both objectives.

<u>Case detection delay</u>

Case detection delay is defined by Muthuvel et al. as the number of months between the onset of signs and symptoms of leprosy and the time of diagnosis, including both "patient delay" and "health-system delay".[55] Several studies have investigated delay in leprosy diagnosis in countries like Bangladesh, Brazil, India, Colombia, and Paraguay.[55–62] However, recent literature on delay in diagnosis is limited and mainly focuses on other geographical regions. Therefore, delay in this study will be determined with a, for this project, structured questionnaire designed in the project countries, with input from several stakeholders, which will be shared open access (publication expected). The questionnaire includes two annexes: a set of clinical photos of signs of leprosy and a context-specific calendar indicating important local dates, such as festivities, agricultural seasons and religious celebrations.[63,64] The questionnaires were culturally validated in all three countries, based on the conceptual framework of Herdman et al. (publication expected).[65]

Table 3. PEP4LEP project outcomes and statistical methods

Objective	Outcome	Hypothesis	Outcome measure	Method of analysis

For peer review only - http://bmjopen.bmj.com/site/about/guidelines.xhtml

1.1 To compare the effectiveness of a skin camp prophylaxis intervention with a health center-based prophylaxis intervention in terms of the rate of leprosy patients detected and delay in case detection	Primary: Case detection delay	Reduction in case detection delay is expected to be greater in the community-based intervention compared with the health center-based household contact approach	Number of months since first signs or symptoms of leprosy until diagnosis; G2D percentage among newly diagnosed leprosy patients	Descriptive statistics; linear mixed models; non-parametric tests
	Primary: Number of contacts diagnosed with leprosy	The community-based intervention will identify more cases of leprosy from contact screening compared with the health center household contact-based approach	Number of contacts diagnosed with leprosy; child proportion; female proportion; MB/PB classification of newly diagnosed leprosy patients	Descriptive statistics; Pearson's chi square test; Fisher's exact test; multivariate logistic regression analysis
	Primary: Number of contacts who received chemoprophylax is	The community-based intervention will allow more contacts to be screened and receive SDR-PEP compared with the health center-based household contact approach	Number of contacts screened; number of contacts who received SDR-PEP	Descriptive statistics
1.2 To compare the feasibility of the two chemoprophylaxis interventions (screening household contacts	Secondary: Cost-effectiveness of each intervention	The community-based intervention will be more expensive but will have a greater impact compared	Number of index patients included; number of contacts screened; number of cases	Health economic evaluations

For peer review only - http://bmjopen.bmj.com/site/about/guidelines.xhtml

or screening contacts via skin camps) in terms of cost- effectiveness and acceptability		with the health center-based household contact approach	prevented; number of disabilities avoided; operational costs; out-of-pocket expenses	
	Secondary: Acceptability of each intervention	Both interventions will be accepted in participating countries	Number of index patients included; number of contacts screened; and qualitative methods	Descriptive statistics; qualitative content analysis of interviews; FGDs and potentially observations
2.1 To assess the acceptability of an integrated skin diseases approach and the use of the SkinApp	Additional: Number of contacts diagnosed with other skin diseases	The community-based intervention will identify more cases of other skin diseases from contact screening compared with the health center-based household contact approach	Number of contacts diagnosed with skin diseases including and with NTDs that manifest with skin lesions	Descriptive statistics; Pearson's chi square test; Fisher's exact test; multivariate logistic regression analysis
	Additional: Acceptability of an integrated skin screening approach and the use of the SkinApp	The integrated skin screening approach will encourage screening participation, and the SkinApp will help health workers to diagnose skin diseases	Number of contacts diagnosed with skin diseases and with NTDs that manifest with skin lesions; utilization of the SkinApp during contact screening; and qualitative methods	Descriptive statistics; sensitivity and specificity; positive and negative predictive values; qualitative content analysis of interviews, FGDs, and

For peer review only - http://bmjopen.bmj.com/site/about/guidelines.xhtml

				potentially observations
2.2 To compare the capacity of health workers in diagnosing leprosy, other skin diseases and other NTDs that manifest with skin lesions before the start of the study with their capacity in the third year	Additional: Capacity of health workers in diagnosing leprosy and other skin diseases	Participation in training and the use of the SkinApp will improve health worker capacity	Results of health worker capacity assessments and qualitative methods	Descriptive statistics; qualitative content analysis of interviews, FGDs, and potentially observations

Abbreviations: FGD: focus group discussion; G2D: grade-2 disability; MB: multibacillary; NTD: neglected tropical disease; PB: paucibacillary; SDR-PEP: single-dose rifampicin

<u>Sample size</u>

The sample size calculation was based on case detection delay as main outcome measure. The mean or median delay will be compared between both interventions and with the baseline. For the sample size calculation, a literature-based estimated average case detection delay of 24 months for leprosy patients was used, with the conservative assumption that a minimal delay difference of three months would be detected between the two interventions.[66,67] In order to achieve this, we aim to include at least 675 index patients in the study: 270 in the community-based intervention areas (30 per country per year) and 405 new patients in the health center-based intervention areas (45 per country per year). Approximately 100 contacts will be screened per index patient in the community-based intervention areas, and approximately 6 contacts will be screened per index patient in the health center-based intervention areas; thus, a total of approximately 30,000 contacts will be screened (Figure 3). All fully trained health staff involved in the PEP4LEP project will be asked to consent to enroll in the capacity assessment.

<u>Randomization</u>

PEP4LEP used randomization without blinding at the (clustered) health center level (health centers/posts), ensuring that clusters were similar in size. Blinding is impossible because of the varying interventions' characteristics. Cluster randomization is commonly used when trying to capture the impact of an intervention at community level on both infectiousness and susceptibility.[68] This method is stated to be feasible logistically, and contamination (e.g.,

For peer review only - http://bmjopen.bmj.com/site/about/guidelines.xhtml

For peer review only

information-sharing between participants from both interventions) is unlikely.[68] Randomization was performed using the statistical software package R.[69] Per country, health centers were randomly divided into the community-based intervention or health center-based intervention.

Data collection and management

The PEP4LEP data management plan was developed by Erasmus MC in collaboration with the consortium. Regarding quantitative data, collectors will record their findings onto paper-based forms. Information from the forms will be entered into the Research Electronic Data Capture (REDCap) system from Vanderbilt University.[70] The REDCap software will be linked to a centralized database server hosted by Erasmus MC.

Qualitative data collection will be audio-recorded and/or paper-based. Data will be transcribed (verbatim) and entered into computer-assisted qualitative data analysis software.[71] The transcriptions will be securely stored at Erasmus MC after analysis. A system of identification (ID) codes has been developed to record and maintain data systematically, as well as to maintain "pseudo-anonymity."

Data analysis

Data from the PEP4LEP study will be analyzed primarily through quantitative methods using descriptive analysis for all variables (Table 3). Mean and median case detection delays will be compared between both interventions and the established baseline. This includes newly diagnosed cases identified through each contact screening intervention as well as those detected through ongoing passive case finding, the current main method of detection in routine leprosy programs in the three countries. The p-values for each statistical test will be two-tailed with $p \leq 0.05$ considered significant and 95% confidence intervals (CI) presented for regression analyses. Quantitative analysis will be conducted using statistical software such as SPSS.[72]

The acceptability and capacity assessments will include qualitative research data (Table 3), which will be coded and analyzed using computer-assisted qualitative data analysis software, including Atlas.ti.[71,73] Data coding is necessary to categorize and define what the data signify by identifying concepts, patterns, relations, and themes.[74] Data reanalysis will occur until no new topics are emerging and data saturation is reached.[75]

Availability of data and materials

Data will be stored for 25 years according to EU regulation 536/2014 considering clinical medication-related research projects.[59] Data will be made available in a repository for potential authorized re-use for future data analysis or study replication. Sharing data and study materials as well as open access publishing are important values of the EU research and innovation

For peer review only - http://bmjopen.bmj.com/site/about/guidelines.xhtml

program Horizon 2020, the European and Developing Countries Clinical Trials Partnership (EDCTP) and the PEP4LEP consortium.[59,76]

Patient and public involvement

Community leaders, people affected by leprosy, and representatives of disabled people organizations (DPO) are and will be involved in monitoring the study as well as in mobilizing community participation.

Ethics

Ethical approval was obtained in Ethiopia, Tanzania, and Mozambique according to national guidelines. Erasmus MC, as European consortium member, received a waiver of full medical ethics review and approval from its ethical board according to the Dutch Medical Research Involving Human Subjects Act (Wet Medisch-Wetenschappelijk Onderzoek met mensen, WMO).[77]

Written (or thumbprint) informed consent will be obtained from all study participants. If a participant is below 18 years old, a parent or legal guardian will be asked for consent. Study information given to the study participants contains the study purpose, the right to withdraw, possible side effects of SDR-PEP (i.e., urine discoloration), the incidental findings procedure and national contact information. AEs are expected to be rare after SDR-PEP. In LPEP program's interim analysis, one adverse event was reported (a severe allergic reaction to rifampicin in Brazil) after administering SDR-PEP to 109,727 contacts of leprosy patients in seven countries.[44] Nevertheless, in (chemo)prophylaxis programs AEs are of utmost importance because large numbers of healthy individuals are involved. In PEP4LEP, SAEs will be reported following national pharmacovigilance guidelines and by using the PEP4LEP AE Form for registration and to inform the principal investigator.[10,54] An emergency allergy kit was recommended to be available at community study sites where no health center is located. All participants with suspected AEs will be referred for proper medical management and treated free of charge according to national standard treatment guidelines.[54]

During both screening interventions and research projects involving human subjects, incidental findings with potential health importance may be observed.[78] Incidental findings are discoveries made during a research or screening project which are outside the scope of the project.[79] Examples of possible incidental findings when performing full body skin screening include: signs

For peer review only - http://bmjopen.bmj.com/site/about/guidelines.xhtml

of cancer, venous insufficiency, bleeding diathesis, herniation, dental problems, or indications of possible abuse. Incidental findings in a research setting are often not explicit enough to be used for diagnosis, treatment, or clinical care.[80]
The procedures for reporting both SAEs and incidental findings are included in the evidence-based PEP4LEP SOPs, on the participant information sheet and in the health workers' training [54,78,79,81,82] The importance will also be emphasized during ongoing monitoring activities, including field visits.[44]

During the developmental phase of this project, the COVID-19 pandemic emerged. Regarding COVID-19, national governmental and WHO guidelines will be followed.[31–34] Information about COVID-19 and project implications (e.g., physical distancing, working in time slots) are included in the project's SOPs, IEC-materials and health workers' training. Hand washing facilities and personal protective equipment (PPE) such as gloves, face masks and aprons, will be made available at the study sides.

A code of conduct will be designed for the PEP4LEP consortium, based on the code of conducts from WHO and All European Academies (ALLEA).[83,84] All researchers in the project are encouraged to participate in good clinical practice (GCP) courses, facilitated by the research consortium.[85] National data-safety monitoring boards, an international publication committee, and an international scientific steering committee were formed to monitor the project (Figure 1).

<u>Trial registration</u>

The PEP4LEP project is registered at The Netherlands Trial Register (NTR), receiving trial registration number NL7294 (NTR7503), registration date September 10, 2018.[86]

Discussion

The PEP4LEP study will use an integrated skin screening approach, which is also recommended by the WHO.[1,16,17] Skin diseases are among the most common human illnesses, affecting almost 900 million people worldwide.[20] They are thought to be the fourth leading cause of global non-fatal disease burden and can result in disabilities, stigmatization, and discrimination.[20,87] Additionally, dermatological problems can be the first expression of systemic or chronic diseases, including HIV/AIDS, diabetes, and NTDs.[14,88] Integrated skin screening is therefore expected to generate a greater health benefit compared with vertical health programs which focus on one disease only. Pooling diseases in projects like PEP4LEP can also be helpful in educating and in raising awareness, as health workers' knowledge of NTDs like leprosy has been declining.[51,89,90]

For peer review only - http://bmjopen.bmj.com/site/about/guidelines.xhtml

This was reflected in a study performed by Abeje et al. among general health workers diagnosing leprosy in Ethiopia, which revealed that only 18% diagnosed leprosy correctly.[91] Detecting skin NTDs like leprosy therefore requires capacity-strengthening programs.[14,16,22–26]

This study will also use mHealth solutions to support peripheral health workers in recognizing and treating signs and symptoms of skin diseases. Evidence indicates that mobile technology tools can substantially benefit healthcare workers, their patients, and adequate health care delivery.[92] In dermatology, electronic health (eHealth) was adopted early, with teledermatology as a widespread example, fostering the possibility of remote patient care and education.[93,94] This is especially valuable if health services are scarce and during periods of service disruption (e.g., flooding, civil unrest, COVID-19 pandemic).[34,52,94–96] We emphasize the importance of studying the effect of mHealth technologies, aimed at capacity strengthening, like NLR's SkinApp, before fully focusing on upscaling.[27,28,92,96]

Despite the conclusion of the expert meeting that SDR-PEP poses negligible risk of generating rifampicin resistance in *M. tuberculosis*, ongoing resistance surveillance is important to consider.[13,97–99] However, because of the limited study period, resistance surveillance in the PEP4LEP implementation areas alone would add no value to the project as the number of patients will be too small and the project duration would be too short for any resistance to emerge during that period. It is therefore recommended to integrate the surveillance of rifampicin resistance in the PEP4LEP project areas with the resistance surveillance systems for TB and leprosy during the project period and beyond, consistent with WHO recommendations on resistance surveillance.[97–99]

Although SDR-PEP has been adopted in the WHO guidelines on leprosy, little is known about the feasibility of several implementation methods of SDR as chemoprophylaxis for leprosy in combination with varying and integrated contact screening methods, especially in sub-Saharan Africa.[4] Tanzania was the only sub-Sahara African country included in the LPEP Program.[11] Ortuno-Gutierrez et al. recently outlined the Post-Exposure Prophylaxis for Leprosy in the Comoros and Madagascar (PEOPLE) study protocol.[100] PEOPLE assesses the effectiveness of different modalities of SDR-PEP, using door-to-door surveys and a double dose of SDR-PEP. Both the PEOPLE and the PEP4LEP research questions comply with the Aligned Research Agenda for Zero Leprosy from the Global Partnership for Zero Leprosy (GPZL) regarding the call for more operational studies and research focusing on SDR-PEP and on digital health.[101,102] Too often, innovative medical interventions fail because the factors contributing to success are poorly understood and hence not considered.[103] Therefore, our goal is to share key insights gained from the PEP4LEP study to foster the implementation of integrated skin screening and chemoprophylaxis for leprosy in the sub-Sahara African context.

For peer review only - http://bmjopen.bmj.com/site/about/guidelines.xhtml

Declarations

Acknowledgments

Our thanks go to all those involved in the PEP4LEP project, including the study participants; the full research consortium; and our funders European and Developing Countries Clinical Trials Partnership (EDCTP) and Leprosy Research Initiative (LRI).

Author contributions

LM, CK, JHR, AS, TH and RvW designed the study. KB, FM, SEM, EM, AM, NM, TL, AMM, DVK, AME, LR, BN supported the development of country-specific protocols, materials and coordinate the study implementation. AS, TH and RvW have drafted the manuscript. All authors have reviewed the draft manuscript and have read and approved the final version.

Funding

This project was supported by the EDCTP2 program under Horizon 2020 (grant number RIA2017NIM-1839-PEP4LEP). The project also received funding from the Leprosy Research Initiative (LRI; www.leprosyresearch.org) under LRI grant number 707.19.58. Both funding bodies reviewed and approved the study proposal.

Competing interests

No competing interest have been declared by the authors.

For peer review only

References

1. World Health Organization. Promoting an integrated approach to enhance detection of neglected tropical diseases of the skin. WHO. https://www.who.int/neglected_diseases/news/Promoting-integrated-approach-to-enhance-detection-skin-NTDs/en/. Published 2019. Accessed June 19, 2019.
2. World Health Organization. Global leprosy update, 2018: moving towards a leprosy-free world. *WHO Wkly Epidemiol Rec*. 2019;94(35/36):389-411. https://www.who.int/wer/2019/wer9435_36/en/.
3. Smith CS, Noordeen SK, Richardus JH, et al. A strategy to halt leprosy transmission. *Lancet Infect Dis*. 2014;14(2):96-98. doi:10.1016/S1473-3099(13)70365-7
4. World Health Organization. *Guidelines for the Diagnosis, Treatment and Prevention of Leprosy.*; 2018. http://nlep.nic.in/pdf/WHO Guidelines for leprosy.pdf.

For peer review only - http://bmjopen.bmj.com/site/about/guidelines.xhtml

5. Van Beers SM, Hatta M, Klatser PR. Patient contact is the major determinant in incident leprosy: Implications for future control. *Int J Lepr Other Mycobact Dis*. 1999;67(2):119-128.
6. Moet FJ, Pahan D, Schuring RP, Oskam L, Richardus JH. Physical distance, genetic relationship, age, and leprosy classification are independent risk factors for leprosy in contacts of patients with leprosy. *J Infect Dis*. 2006;193(3):346-353. doi:10.1086/499278
7. Smith WC, van Brakel W, Gillis T, Saunderson P, Richardus JH. The Missing Millions: A Threat to the Elimination of Leprosy. Lockwood DNJ, ed. *PLoS Negl Trop Dis*. 2015;9(4):e0003658. doi:10.1371/journal.pntd.0003658
8. Moet FJ, Pahan D, Oskam L, Richardus JH. Effectiveness of single dose rifampicin in preventing leprosy in close contacts of patients with newly diagnosed leprosy: cluster randomised controlled trial. *Bmj*. 2008;336(7647):761-764.
9. Idema WJ, Majer IM, Pahan D, Oskam L, Polinder S, Richardus JH. Cost-effectiveness of a chemoprophylactic intervention with single dose rifampicin in contacts of new leprosy patients. *PLoS Negl Trop Dis*. 2010;4(11):e874-e874. doi:10.1371/journal.pntd.0000874
10. Barth-Jaeggi T, Steinmann P, Mieras L, et al. Leprosy Post-Exposure Prophylaxis (LPEP) programme: study protocol for evaluating the feasibility and impact on case detection rates of contact tracing and single dose rifampicin. *BMJ Open*. 2016;6(11):e013633.
11. Richardus JH, Barth-Jaeggi T, Cavaliero A, et al. Emerging evidence from the Leprosy Post-Exposure Prophylaxis (LPEP) program. In: *ECTMIH Antwerp*. ; 2017:62. https://www.ectmih2017.be/site/assets/files/1014/abstracts_organised_sessions-1.pdf.
12. Tiwari A, Blok DJ, Arif M, Richardus JH. Leprosy post-exposure prophylaxis in the Indian health system: A cost-effectiveness analysis. *PLoS Negl Trop Dis*. 2020;14(8):e0008521. doi:10.1371/journal.pntd.0008521
13. Mieras L, Anthony R, van Brakel W, et al. Negligible risk of inducing resistance in Mycobacterium tuberculosis with single-dose rifampicin as post-exposure prophylaxis for leprosy. *Infect Dis poverty*. 2016;5(1):46.
14. Yotsu R, Yotsu, R. R. Integrated Management of Skin NTDs—Lessons Learned from Existing Practice and Field Research. *Trop Med Infect Dis*. 2018;3(4):120. doi:10.3390/tropicalmed3040120
15. World Health Organization. *Recognizing Neglected Tropical Diseases through Changes on the Skin*. World Health Organization; 2018. http://www.who.int/neglected_diseases/resources/9789241513531/en/. Accessed July 27, 2020.
16. Mitjà O, Marks M, Bertran L, et al. Integrated control and management of neglected tropical skin diseases. *PLoS Negl Trop Dis*. 2017;11(1):e0005136.
17. Hay RJ, Asiedu K. Skin-Related Neglected Tropical Diseases (Skin NTDs)—A New Challenge. *Trop Med Infect Dis*. 2018;4(1):4. doi:10.3390/tropicalmed4010004

For peer review only - http://bmjopen.bmj.com/site/about/guidelines.xhtml

18. Kabatereine NB, Malecela M, Lado M, Zaramba S, Amiel O, Kolaczinski JH. How to (or Not to) Integrate Vertical Programmes for the Control of Major Neglected Tropical Diseases in Sub-Saharan Africa. Brooker S, ed. *PLoS Negl Trop Dis*. 2010;4(6):e755. doi:10.1371/journal.pntd.0000755
19. Ryan TJ, Ersser SJ, Fuller LC. The public health intervention of skin care for all: community dermatology. In: *Public Health-Social and Behavioral Health*. InTech; 2012.
20. Hay RJ, Johns NE, Williams HC, et al. The global burden of skin disease in 2010: an analysis of the prevalence and impact of skin conditions. *J Invest Dermatol*. 2014;134(6):1527-1534.
21. United Nations. World Population Prospects 2019. https://population.un.org/wpp/DataQuery/. Accessed April 23, 2020.
22. Abdela SG, Diro E, Zewdu FT, et al. Looking for NTDs in the skin; an entry door for offering patient centered holistic care. *J Infect Dev Ctries*. 2020;14(61):16S-21S. doi:10.3855/jidc.11707
23. Figueroa JI, Fuller LC, Abraha A, Hay RJ. Dermatology in southwestern Ethiopia: rationale for a community approach. *Int J Dermatol*. 1998;37(10):752-758.
24. Hay R, Estrada R, Grossmann H. Managing skin disease in resource-poor environments - the role of community-oriented training and control programs. *Int J Dermatol*. 2011;50(5):558-563. doi:10.1111/j.1365-4632.2011.04954.x
25. Faye O, Hay RJ, Ryan TJ, Keita S, Traore AK, Mahe A. A public health approach for leprosy detection based on a very short term-training of primary health care workers in basic dermatology. *Lepr Rev*. 2007;78(1):11.
26. Maheé A, N'diaye HT, Bobin P. The proportion of medical consultations motivated by skin diseases in the health centers of Bamako (Republic of Mali). *Int J Dermatol*. 1997;36(3):185-186.
27. NLR. SkinApp. https://leprosyrelief.org/skinapp. Accessed July 30, 2018.
28. Mieras L, Taal A, Post E, Ndeve A, van Hees C. The development of a mobile application to support peripheral health workers to diagnose and treat people with skin diseases in resource-poor settings. *Trop Med Infect Dis*. 2018;3(3):102.
29. World Health Organization. Global leprosy update, 2018: moving towards a leprosy-free world. *WHO Wkly Epidemiol Rec*. 2019;94(35/36):389-411. https://apps.who.int/iris/handle/10665/326776.
30. Khoudri I, Elyoussfi Z, Mourchid Y, et al. Trend analysis of leprosy in Morocco between 2000 and 2017: Evidence on the single dose rifampicin chemoprophylaxis. *PLoS Negl Trop Dis*. 2018;12(12):e0006910. doi:10.1371/journal.pntd.0006910
31. World Health Organization. Coronavirus disease (COVID-19). https://www.who.int/emergencies/diseases/novel-coronavirus-2019. Accessed July 17,

For peer review only - http://bmjopen.bmj.com/site/about/guidelines.xhtml

2020.

32. World Health Organization. *Considerations for Implementing Mass Treatment, Active Case-finding and Population-Based Surveys for Neglected Tropical Diseases in the Context of the COVID-19 Pandemic.*; 2020. https://www.who.int/publications/i/item/WHO-2019-nCoV-neglected-tropical-diseases-2020-1. Accessed August 3, 2020.

33. Government of the Netherlands. Frequently asked questions about coronavirus and health | Coronavirus COVID-19 | Government.nl. https://www.government.nl/topics/coronavirus-covid-19/frequently-asked-questions-about-coronavirus-and-health. Accessed July 17, 2020.

34. World Health Organization. COVID-19 significantly impacts health services for noncommunicable diseases. World Health Organisation. https://www.who.int/news-room/detail/01-06-2020-covid-19-significantly-impacts-health-services-for-noncommunicable-diseases. Published 2020. Accessed July 24, 2020.

35. Mayo Clinic Staff. Tuberculosis. Diseases & Conditions. https://www.mayoclinic.org/diseases-conditions/tuberculosis/symptoms-causes/syc-20351250. Published 2018. Accessed July 30, 2018.

36. Gajuryal SH, Gautam S, Satyal N, Pant B. Organizing a Health Camp: Management Perspective. *Nepal Med J*. 2019;2(1):196-198. doi:10.3126/nmj.v2i1.23557

37. Ali M. What Are the Requirements to Organize a Free Medical Camp. https://www.transparenthands.org/what-are-the-requirements-to-organize-a-free-medical-camp/. Published 2018. Accessed June 17, 2019.

38. Grover S, Ranyal RK, Bedi MK. A cross section of skin diseases in rural Allahabad. *Indian J Dermatol*. 2008;53(4):179.

39. Tindana PO, Singh JA, Tracy CS, et al. Grand challenges in global health: Community engagement in research in developing countries. *PLoS Med*. 2007;4(9):1451-1455. doi:10.1371/journal.pmed.0040273

40. Shrestha R, Shrestha DP, Lama L, Gurung D, Rosdahl I. Pattern of skin diseases in a rural village development community of Nepal. *Nepal J Dermatology, Venereol Leprol*. 2014;12(1):41-44.

41. Feenstra SG, Nahar Q, Pahan D, Oskam L, Richardus JH. Acceptability of chemoprophylaxis for household contacts of leprosy patients in Bangladesh: a qualitative study. *Lepr Rev*. 2011;82(2):178-187. http://www.ncbi.nlm.nih.gov/pubmed/21888142. Accessed December 7, 2018.

42. Espiridion-Calma AD V, Dofitas BL, Elinor M, Sison GQ. *Acceptability of Immunoprophylaxis and/or Chemoprophylaxis for Household Contacts of Patients with Hansen's Disease: A Prospective, Single-Center, Mixed Methods Study*. Vol 54.; 2020. https://actamedicaphilippina.upm.edu.ph/index.php/acta/article/view/1663. Accessed July

For peer review only

For peer review only - http://bmjopen.bmj.com/site/about/guidelines.xhtml

27, 2020.
43. Peters R, Mieras L, Subedi M, et al. A single dose of rifampicin to prevent leprosy: Qualitative analysis of perceptions of persons affected, contacts, community members and health professionals towards chemoprophylaxis and the impact on their attitudes in India, Nepal and Indonesia. *Lepr Rev*. 2018;89(4):335-352. https://research.vu.nl/en/publications/a-single-dose-of-rifampicin-to-prevent-leprosy-qualitative-analys. Accessed July 27, 2020.
44. Steinmann P, Cavaliero A, Aerts A, et al. The Leprosy Post-Exposure Prophylaxis (LPEP) programme: Update and interim analysis. *Lepr Rev*. 2018;89(2):102-116.
45. Aarogyasri Health Care Trust. *Revised Health Camp Policy-Guidelines*. https://www.aarogyasri.telangana.gov.in/documents/10202/0/Revised+Health+Camp+Policy.pdf/e5794475-1546-4221-9f63-f9a3ca1d2005. Accessed June 17, 2019.
46. Sathyasai. *Guidelines for Medical Camps Conducted under the The Auspices of Sathya Sai International Organization*.; 2013. www.sathyasai.org/organisation/guidelines/medical-camps.
47. Hoeven TA, Fischer EAJ, Pahan D, Richardus JH. Social distance and spatial distance are not the same, observations on the use of GIS in leprosy epidemiology. *Epidemiol Infect*. 2008;136(12):1624-1627. doi:10.1017/S0950268808000381
48. Fischer E, Vlas DS, Meima A, Habbema D, Richardus J. Different mechanisms for heterogeneity in leprosy susceptibility can explain disease clustering within households. *PLoS One*. 2010;5(11). doi:10.1371/journal.pone.0014061
49. Cavaliero A, Greter H, Fürst T, et al. An innovative approach to screening and chemoprophylaxis among contacts of leprosy patients in low endemic settings: experiences from Cambodia. Small PLC, ed. *PLoS Negl Trop Dis*. 2019;13(3):e0007039. doi:10.1371/journal.pntd.0007039
50. Smith WCS, Aerts A. Role of contact tracing and prevention strategies in the interruption of leprosy transmission. *Lepr Rev*. 2014;85(1):2-17. http://www.ncbi.nlm.nih.gov/pubmed/24974438. Accessed June 19, 2019.
51. Chandler DJ, Fuller LC. The skin—A common pathway for integrating diagnosis and management of NTDs. *Trop Med Infect Dis*. 2018;3(3):101. doi:10.3390/tropicalmed3030101
52. Siilo. The free secure messaging app for medical team players. https://www.siilo.com/. Accessed November 27, 2018.
53. Tiwari A, Dandel S, Djupuri R, Mieras L, Richardus JH. Population-wide administration of single dose rifampicin for leprosy prevention in isolated communities: a three year follow-up feasibility study in Indonesia. *BMC Infect Dis*. 2018;18(1):324.
54. World Health Organization. *Assuring Safety of Preventive Chemotherapy Interventions for*

For peer review only - http://bmjopen.bmj.com/site/about/guidelines.xhtml

the Control of Neglected Tropical Diseases Practical Advice for National Programme Managers on the Prevention, Detection and Management of Serious Adverse Events.; 2011. http://www.who.int/about/licensing/. Accessed July 27, 2020.

55. Muthuvel T, Govindarajulu S, Isaakidis P, et al. "I Wasted 3 Years, Thinking It's Not a Problem": Patient and Health System Delays in Diagnosis of Leprosy in India: A Mixed-Methods Study. *PLoS Negl Trop Dis*. 2017;11(1):1-15. doi:10.1371/journal.pntd.0005192

56. Fischer EAJ, de Vlas SJ, Habbema JDF, Richardus JH. The long term effect of current and new interventions on the new case detection of leprosy: a modeling study. *PLoS Negl Trop Dis*. 2011;5(9):e1330. doi:10.1371/journal.pntd.0001330

57. Henry M, GalAn N, Teasdale K, et al. Factors Contributing to the Delay in Diagnosis and Continued Transmission of Leprosy in Brazil – An Explorative, Quantitative, Questionnaire Based Study. *PLoS Negl Trop Dis*. 2016;10(3):1-12. doi:10.1371/journal.pntd.0004542

58. Deps PD, Guedes BVS, Filho B, Andreatta MK, Marcari RS, Rodrigues LC. Delay in the diagnosis of leprosy in the Metropolitan Region of Vito. *Lepr Rev*. 2006;77(1):41-47.

59. European Commission. Guidelines on Open Access to Scientific Publications and Research Data in Horizon 2020, Version 2.1. 2016;(February):1-10. https://ec.europa.eu/research/participants/data/ref/h2020/grants_manual/hi/oa_pilot/h2020-hi-oa-pilot-guide_en.pdf. Accessed July 31, 2018.

60. Srinivas G, Muthuvel T, Lal V, Vaikundanathan K, Schwienhorst-Stich EM, Kasang C. Risk of disability among adult leprosy cases and determinants of delay in diagnosis in five states of India: A case-control study. *PLoS Negl Trop Dis*. 2019;13(6). doi:10.1371/journal.pntd.0007495

61. Gómez L, Rivera A, Vidal Y, et al. Factors associated with the delay of diagnosis of leprosy in north-eastern Colombia: a quantitative analysis. *Trop Med Int Heal*. 2018;23(2):193-198. doi:10.1111/tmi.13023

62. Nicholls PG, Wiens C, Smith WCS. Delay in Presentation in the Context of Local Knowledge and Attitude Towards Leprosy—The Results of Qualitative Fieldwork in Paraguay. *Int J Lepr Other Mycobact Dis*. 2003;71(3):198. doi:10.1489/1544-581X(2003)71<198:DIPITC>2.0.CO;2

63. Schmier J, Halpern MT. Patient recall and recall bias of health state and health status. *Expert Rev Pharmacoeconomics Outcomes Res*. 2004;4(2):159-163. doi:10.1586/14737167.4.2.159

64. Stull DE, Leidy NK, Parasuraman B, Chassany O. Optimal recall periods for patient-reported outcomes: Challenges and potential solutions. *Curr Med Res Opin*. 2009;25(4):929-942. doi:10.1185/03007990902774765

65. Herdman M, Fox-Rushby J, Badia X. A model of equivalence in the cultural adaptation of HRQoL instruments: The universalist approach. *Qual Life Res*. 1998;7(4):323-335.

For peer review only

For peer review only - http://bmjopen.bmj.com/site/about/guidelines.xhtml

doi:10.1023/A:1008846618880

66. Li J, Yang L, Wang Y, Liu H, Liu J, Cross H. How to improve early case detection in low endemic areas with pockets of leprosy: A study of newly detected leprosy patients in Guizhou Province, People's Republic of China. *Lepr Rev*. 2016;87(1):23-31.

67. Van Veen NHJ, Meima A, Richardus JH. The relationship between detection delay and impairment in leprosy control: a comparison of patient cohorts from Bangladesh and Ethiopia. *Lepr Rev*. 2006;77(4):356.

68. Hayes RJ, Alexander NDE, Bennett S, Cousens SN. Design and analysis issues in cluster-randomized trials of interventions against infectious diseases. *Stat Methods Med Res*. 2000;9(2):95-116. doi:10.1191/096228000670953670

69. The R Foundation. The R Project for Statistical Computing. https://www.r-project.org/. Accessed November 9, 2018.

70. Vanderbilt University. About – REDCap. https://projectredcap.org/about/. Accessed February 24, 2020.

71. Denzin NK, Lincoln YS. *Collecting and Interpreting Qualitative Materials*. Sage Publications; 2008. https://books.google.co.in/books?hl=nl&lr=&id=ocGxhJEMf0kC&oi=fnd&pg=PR5&dq=ethnography+focus+group+observation+interviews&ots=teTaeexex4&sig=GV0ayItmc-CfhKAERa_RQ51NT-8&redir_esc=y#v=onepage&q=ethnography focus group observation interviews&f=false. Accessed December 7, 2018.

72. International Business Machines Corporation. IBM SPSS Software. https://www.ibm.com/analytics/spss-statistics-software. Accessed May 1, 2020.

73. ATLAS.ti: The Qualitative Data Analysis & Research Software. ATLAS.ti. https://atlasti.com/. Accessed March 23, 2020.

74. Sage GG-A qualitative data. L, 2007 undefined. Thematic coding and categorizing. *methods.sagepub.com*. https://methods.sagepub.com/base/download/BookChapter/analyzing-qualitative-data/n4.xml. Accessed February 24, 2020.

75. Morse JM. The significance of saturation. *Qual Health Res*. 1995;5(2):147-149. doi:10.1177/104973239500500201

76. European and Developing Countries Clinical Trials Partnership. *EDCTP2 Policy on Clinical Trials Registration, Publication and Data*.; 2018. http://www.edctp.org/web/app/uploads/2018/07/EDCTP2_policy_on_registering_and_reporting_clinical_studies-1.pdf.

77. Centrale Commissie Mensgebonden Onderzoek. Uw onderzoek: WMO-plichtig of niet? https://www.ccmo.nl/onderzoekers/wet-en-regelgeving-voor-medisch-wetenschappelijk-onderzoek/uw-onderzoek-wmo-plichtig-of-niet. Accessed July 31, 2018.

For peer review only

For peer review only - http://bmjopen.bmj.com/site/about/guidelines.xhtml

78. Wolf SM, Lawrenz FP, Nelson CA, et al. Managing Incidental Findings in Human Subjects Research: Analysis and Recommendations. *J Law, Med Ethics*. 2008;36(2):219-248. doi:10.1111/j.1748-720X.2008.00266.x

79. University of Waterloo - Office of Research Ethics. *Guideline for the Reporting of Incidental and Secondary Findings to Study Participants University of Waterloo Office of Research Ethics.*; 2014. https://uwaterloo.ca/research/sites/ca.research/files/uploads/files/guideline_on_incidental_findings_reporting_october_2014.pdf.

80. Phung C. Ethics of disclosing results of genetic testing of donor-derived leukemia to recipient in a hereditary cancer biology research setting Connie Phung, MS 1The. *bioethics.yale.edu*. https://bioethics.yale.edu/sites/default/files/files/Ethics of Donor Derived Leukemia.pdf. Accessed December 13, 2018.

81. Illes J, Kirschen MP, Edwards E, et al. Ethics. Incidental findings in brain imaging research. *Science*. 2006;311(5762):783-784. doi:10.1126/science.1124665

82. Council of Europe. *Convention for the Protection of Human Rights and Dignity of the Human Being with Regard to the Application of Biology and Medicine: Convention on Human Rights and Biomedicine.*; 1997. https://www.coe.int/en/web/conventions/full-list/-/conventions/treaty/164. Accessed May 11, 2020.

83. World Health Organization. *WHO Ethics: Promoting Compliance, Risk Management and Ethics.*; 2003. www.who.int/about/ethics. Accessed January 8, 2020.

84. All European Academies. *The European Code of Conduct for Research Integrity*. ALLEA; 2017. www.allea.org. Accessed October 20, 2020.

85. European Union. *Good Clinical Practice.*; 1997. https://ec.europa.eu/health/sites/health/files/files/eudralex/vol-10/3cc1aen_en.pdf. Accessed August 6, 2018.

86. Netherlands Trial Register. Trial NL7294 (NTR7503) - PEP4LEP. https://www.trialregister.nl/trial/7294. Accessed June 19, 2019.

87. Mphande FA. Skin Diseases: Need for Attention. In: *Skin Disorders in Vulnerable Populations*. Springer Singapore; 2020:1-12. doi:10.1007/978-981-15-3879-7_1

88. Engelman D, Fuller LC, Solomon AW, et al. Opportunities for Integrated Control of Neglected Tropical Diseases That Affect the Skin. *Trends Parasitol*. 2016;32(11):843-854. doi:10.1016/j.pt.2016.08.005

89. Henderson CA. Skin disease in rural Tanzania. *Int J Dermatol*. 1996;35(9):640-642.

90. Muloliwa AM, Dreva D, Banquimane M, et al. Descrição da tendência de registo de casos de lepra em três distritos de Nampula, 2014-2018. *II Jornadas Reg Saúde - Região Norte, Programa Científico e Livro Resumo*. 2019:61.

91. Abeje T, Negera E, Kebede E, et al. Performance of general health workers in leprosy

For peer review only

For peer review only - http://bmjopen.bmj.com/site/about/guidelines.xhtml

control activities at public health facilities in Amhara and Oromia States, Ethiopia. *BMC Health Serv Res*. 2016;16(1):122.

92. White A, Thomas DSK, Ezeanochie N, Bull S. Health Worker mHealth Utilization: A Systematic Review. *CIN - Comput Informatics Nurs*. 2016;34(5):206-214. doi:10.1097/CIN.0000000000000231

93. Faye O, Bagayoko C, Dicko A, et al. A Teledermatology Pilot Programme for the Management of Skin Diseases in Primary Health Care Centres: Experiences from a Resource-Limited Country (Mali, West Africa). *Trop Med Infect Dis*. 2018;3(3):88. doi:10.3390/tropicalmed3030088

94. Wurm EMT, Hofmann-Wellenhof R, Wurm R, Soyer HP. Telemedicine and teledermatology: Past, present and future. *JDDG*. 2008;6(2):106-112. doi:10.1111/j.1610-0387.2007.06440.x

95. Källander K, Tibenderana JK, Akpogheneta OJ, et al. Mobile health (mhealth) approaches and lessons for increased performance and retention of community health workers in lowand middle-income countries: A review. *J Med Internet Res*. 2013;15(1):e17. doi:10.2196/jmir.2130

96. Aranda-Jan CB, Mohutsiwa-Dibe N, Loukanova S. Systematic review on what works, what does not work and why of implementation of mobile health (mHealth) projects in Africa. *BMC Public Health*. 2014;14(1):188. doi:10.1186/1471-2458-14-188

97. World Health Organization. *Guidelines for Surveillance of Drug Resistance in Tuberculosis - Fourth Edition*.; 2009.

98. World Health Organization. A guide for surveillance of antimicrobial resistance in leprosy: 2017 update. *WHO*. August 2017. https://www.who.int/lep/resources/9789290226192/en/. Accessed April 6, 2020.

99. Schoenmakers A, Mieras L, Budiawan T, van Brakel WH. The State of Affairs in Post-Exposure Leprosy Prevention: A Descriptive Meta-Analysis on Immuno- and Chemo-Prophylaxis. *Res Rep Trop Med*. 2020;Volume 11:97-117. doi:10.2147/RRTM.S190300

100. Ortuno-Gutierrez N, Younoussa A, Randrianantoandro A, et al. Protocol, rationale and design of PEOPLE (Post ExpOsure Prophylaxis for LEprosy in the Comoros and Madagascar): a cluster randomized trial on effectiveness of different modalities of implementation of post-exposure prophylaxis of leprosy contacts. *BMC Infect Dis*. 2019;19(1):1033. doi:10.1186/s12879-019-4649-0

101. Global Partnership for Zero Leprosy. Action Framework for Zero Leprosy. 2019. https://zeroleprosy.org/wp-content/uploads/2019/04/Action-Framework-PPT-slide-12-March-1.pdf.

102. Blok D. GPZL Reports on Research Priorities. *Lepr Rev*. 2019;90:237-289.

103. Wiltsey Stirman S, Kimberly J, Cook N, Calloway A, Castro F, Charns M. The sustainability

For peer review only

For peer review only - http://bmjopen.bmj.com/site/about/guidelines.xhtml

of new programs and innovations: A review of the empirical literature and recommendations for future research. *Implement Sci*. 2012;7(1). doi:10.1186/1748-5908-7-17

For peer review only

For peer review only - http://bmjopen.bmj.com/site/about/guidelines.xhtml

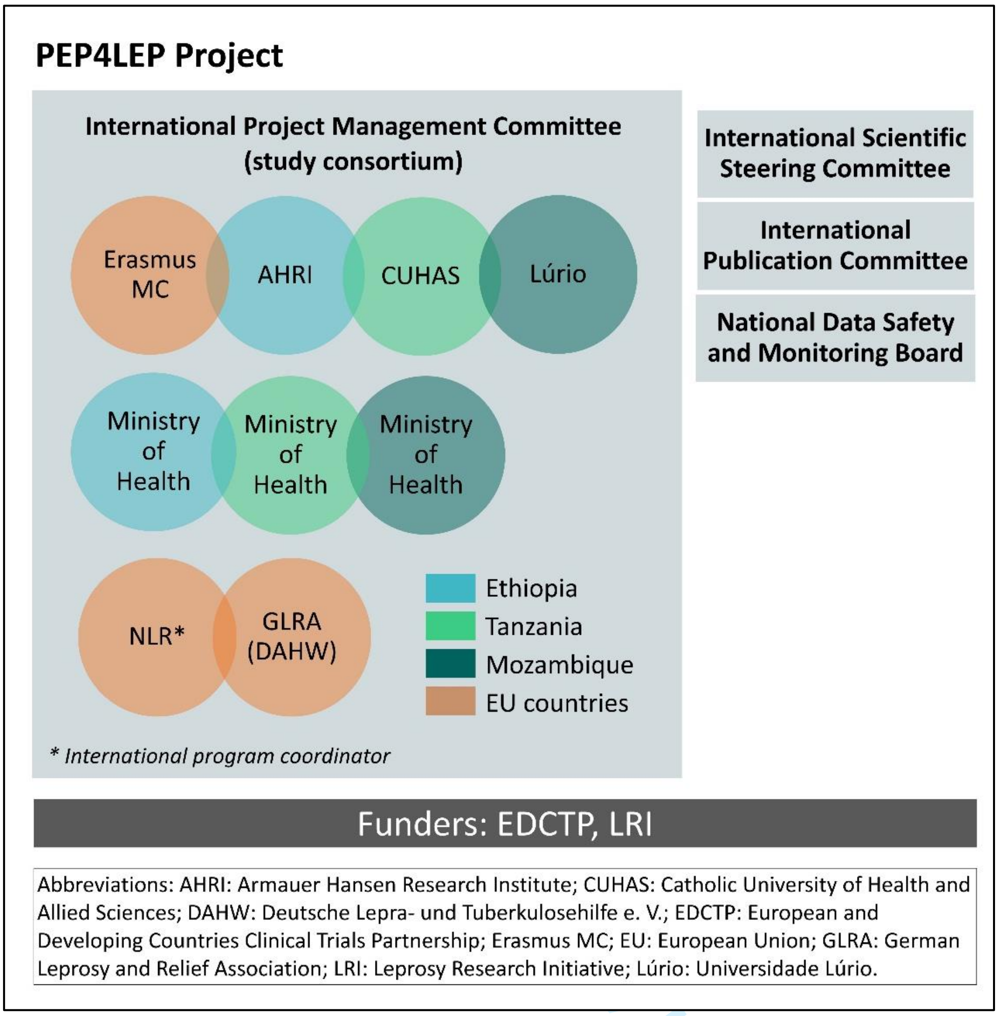

Figure 1. PEP4LEP Project organization chart

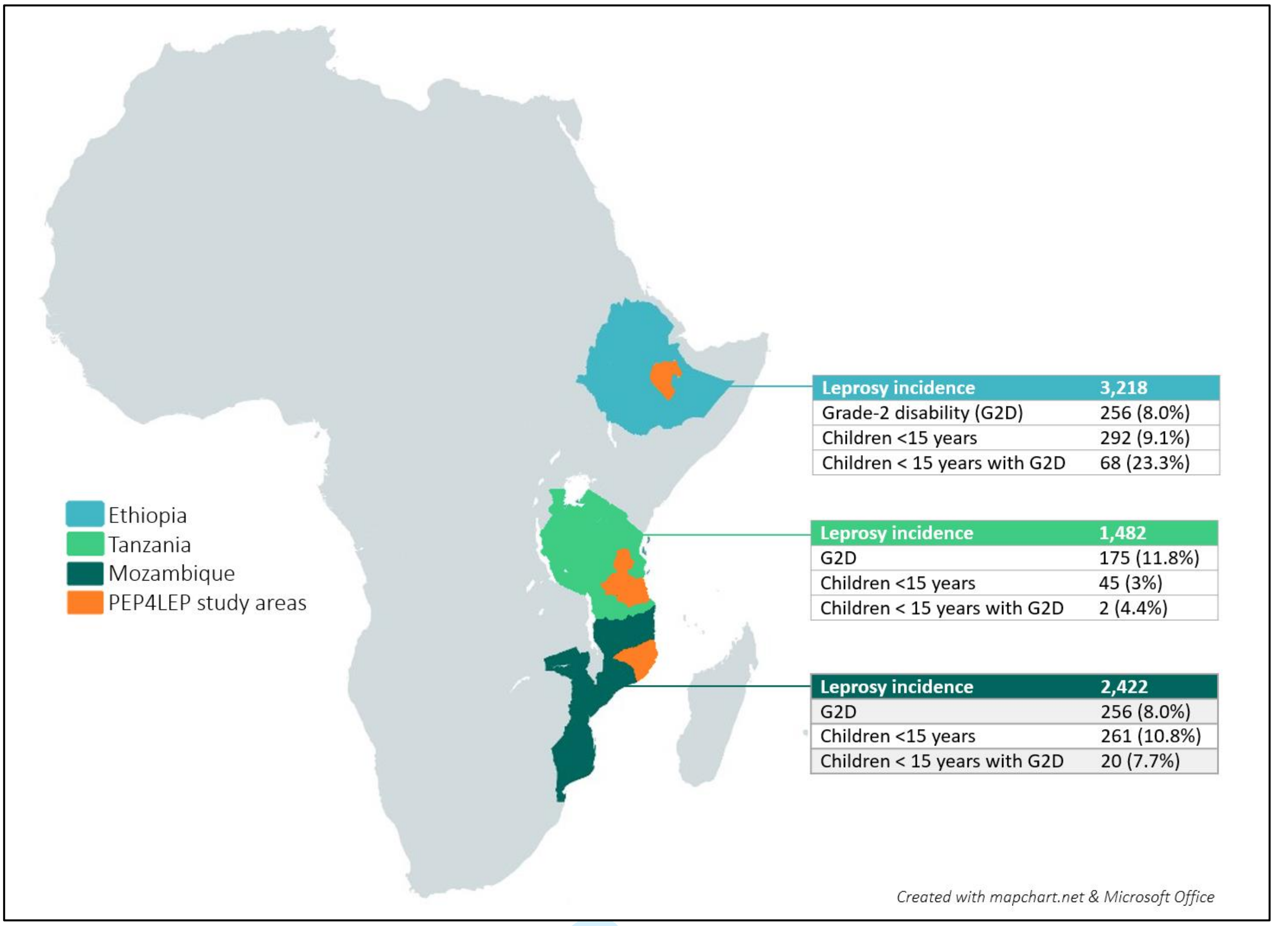

Figure 2. PEP4LEP countries' leprosy incidence (2018) according to the World Health Organization[29]

Table 1. PEP4LEP eligibility criteria[4,10]

	Index patients	Contacts
Inclusion criteria	• Consent to participate in the PEP4LEP project<br>• Diagnosed with leprosy (preferred maximum of 6 months prior to inclusion)<br>• Residence in the PEP4LEP districts for ≥3 months prior to the date of diagnosis<br>• Index patient has started MDT<br>• Community-based skin camp intervention: Leprosy patient gives permission for the set-up of a skin camp in his/her community (sharing their leprosy diagnosis with their contacts is not needed)<br>• Health center-based household screening intervention: Leprosy patient with household contacts, and who is willing to inform these contacts about PEP4LEP	• Consent to participate in the PEP4LEP project<br>• Community-based skin camp intervention: Community contact of the index patient for ≥3 months<br>• Health center-based household screening intervention: Contact which is a household member of the index patient for ≥3 months, visiting the screening health center ≤3 months after the index patient was included
Exclusion criteria	• Index patient or parents/legal guardians unable to understand the purpose and risks of participating in the PEP4LEP study	• Contact or parents/legal guardians unable to understand the purpose and risks of participating in the PEP4LEP study<br>• Age <2 years and/or <10 kg of weight*<br>• Pregnancy*<br>• Receiving or having received rifampicin for any reason in the last 2 years<br>• Known allergy to rifampicin<br>• History of liver or renal disorders<br>• Individuals with leprosy and those who have possible signs and/or symptoms of leprosy (e.g., leprosy-like skin lesions or nerve manifestations) until their disease status has been clarified[35]**<br>• Individuals with possible signs and/or symptoms of TB (cough for more than two weeks or cough in known HIV/AIDS patients, night sweats, unexplained fever, weight loss) until their disease status has been clarified[35]***<br>• Individuals with possible signs and/or symptoms of COVID-19 (self-assessed temperature ≥38°C, respiratory or cold-like symptoms, sudden loss of smell/taste) or possible contact with a COVID-19 patient in the past 14 days.[31–34]***

* A voucher will be given for repeated skin screening and SDR-PEP. This can be used in a PEP4LEP affiliated health center when this person becomes eligible (e.g., after giving birth).
** If referral was needed and no leprosy is detected, repeated skin screening and SDR-PEP can be provided in a PEP4LEP affiliated health center.
*** Skin screening and SDR-PEP can only be provided in a PEP4LEP affiliated health center after the contact is tested negative for COVID-19/TB (according to national guidelines).[31–34]

Abbreviations: COVID-19: Coronavirus Disease 2019; MDT: multidrug therapy; SDR-PEP: single-dose rifampicin post-exposure prophylaxis; TB: tuberculosis

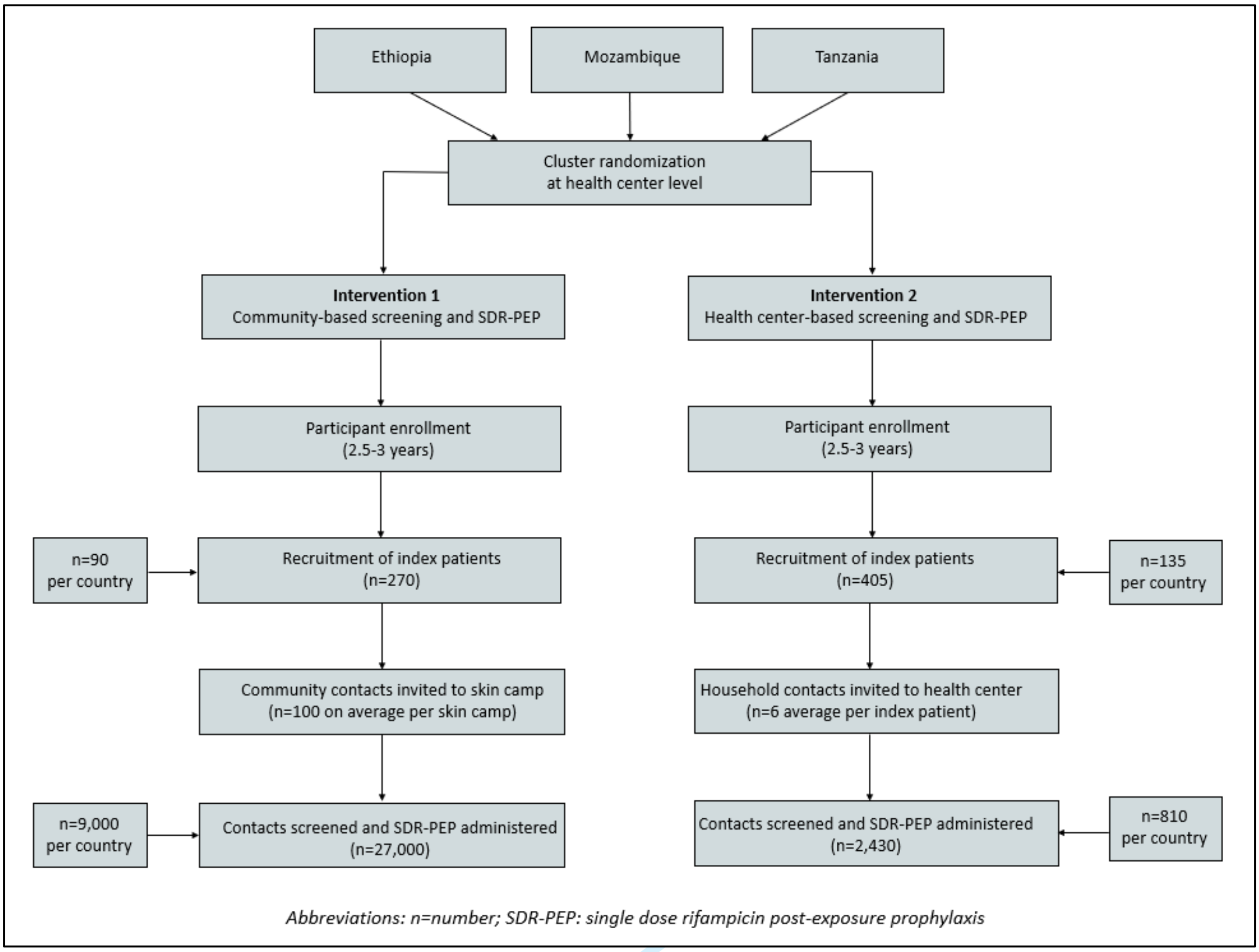

Figure 3. Flow of participants through the PEP4LEP study

Table 2. PEP4LEP single-dose rifampicin dosages[4,10]

Age and body weight of contact	Rifampicin dosage
≥15 years	600 mg
10-14 years	450 mg
6-9 years and body weight of ≥20 kg	300 mg
≥2 years old and body weight between 10-20 kg	150 mg

For peer review only

Table 3. PEP4LEP project outcomes and statistical methods

Objective	Outcome	Hypothesis	Outcome measure	Method of analysis
1.1 To compare the effectiveness of a skin camp prophylaxis intervention with a health center-based prophylaxis intervention in terms of the rate of leprosy patients detected and delay in case detection	Primary: Case detection delay	Reduction in case detection delay is expected to be greater in the community-based intervention compared with the health center-based household contact approach	Number of months since first signs or symptoms of leprosy until diagnosis; G2D percentage among newly diagnosed leprosy patients	Descriptive statistics; linear mixed models; non-parametric tests
	Primary: Number of contacts diagnosed with leprosy	The community-based intervention will identify more cases of leprosy from contact screening compared with the health center household contact-based approach	Number of contacts diagnosed with leprosy; child proportion; female proportion; MB/PB classification of newly diagnosed leprosy patients	Descriptive statistics; Pearson's chi square test; Fisher's exact test; multivariate logistic regression analysis
	Primary: Number of contacts who received chemoprophylaxis	The community-based intervention will allow more contacts to be screened and receive SDR-PEP compared with the health center-based household contact approach	Number of contacts screened; number of contacts who received SDR-PEP	Descriptive statistics
1.2 To compare the feasibility of the two chemoprophylaxis interventions (screening household contacts or screening contacts via skin camps) in terms of cost- effectiveness and acceptability	Secondary: Cost-effectiveness of each intervention	The community-based intervention will be more expensive but will have a greater impact compared with the health center-based household contact approach	Number of index patients included; number of contacts screened; number of cases prevented; number of disabilities avoided; operational costs; out-of-pocket expenses	Health economic evaluations
	Secondary: Acceptability of each intervention	Both interventions will be accepted in participating countries	Number of index patients included; number of contacts screened; and qualitative methods	Descriptive statistics; qualitative content analysis of interviews; FGDs and potentially observations
2.1 To assess the acceptability of an integrated skin diseases approach and the use of the SkinApp	Additional: Number of contacts diagnosed with other skin diseases	The community-based intervention will identify more cases of other skin diseases from contact screening	Number of contacts diagnosed with skin diseases including and with NTDs that manifest with skin lesions	Descriptive statistics; Pearson's chi square test; Fisher's exact test; multivariate logistic regression analysis

		compared with the health center-based household contact approach		
	Additional: Acceptability of an integrated skin screening approach and the use of the SkinApp	The integrated skin screening approach will encourage screening participation, and the SkinApp will help health workers to diagnose skin diseases	Number of contacts diagnosed with skin diseases and with NTDs that manifest with skin lesions; utilization of the SkinApp during contact screening; and qualitative methods	Descriptive statistics; sensitivity and specificity; positive and negative predictive values; qualitative content analysis of interviews, FGDs, and potentially observations
2.2 To compare the capacity of health workers in diagnosing leprosy, other skin diseases and other NTDs that manifest with skin lesions before the start of the study with their capacity in the third year	Additional: Capacity of health workers in diagnosing leprosy and other skin diseases	Participation in training and the use of the SkinApp will improve health worker capacity	Results of health worker capacity assessments and qualitative methods	Descriptive statistics; qualitative content analysis of interviews, FGDs, and potentially observations

Abbreviations: FGD: focus group discussion; G2D: grade-2 disability; MB: multibacillary; NTD: neglected tropical disease; PB: paucibacillary; SDR-PEP: single-dose rifampicin

For peer review only

የሳይንስና ከፍተኛ ትምህርት ሚኒስቴር
Ministry of Science and Higher Education - Ethiopia

Ref.No. MoSHE//RD/124/1/10116/20

Date: 17 FEB 2020

Armuer Hansen Research Institute (AHRI)
Addis Ababa

Subject: Letter of Approval

The Ministry of Science and Higher Education (MoSHE) via its National Research Ethics Review Committee has reviewed **"Comparing the Effectiveness and Feasibility of a Skincamp Intervention to a Healthcentre Based Intervention as Countries Scale-up Use of Chemoprophylaxis for Leprosy"** project protocol in an expedited manner. We are writing to advise you that MoSHE has granted full approval to the above named project, for a period of **one year (February 17, 2020- February 16, 2021)..**

All your most recently submitted documents have been approved for use in this study. The study should comply with the international and national scientific and ethical standard guidelines. Any change to the approved protocol or consent material must be reviewed and approved through the amendment process prior to its implementation. In addition, any adverse or unanticipated events should be reported within 24-48 hours to MoSHE. Please ensure that you submit biannual progress report to MoSHE once in six months and annual renewal application 30 days prior to the expiry date.

We, therefore, request you as PI and your esteemed organization to ensure the commencement and conduct of the study accordingly and wish for the successful completion of the project.

Sincerely

Solomon Benor Belay (PhD)
Director General for Science and Research Affairs

Cc.

- Office of the State Minister (Sector for Science, Research and Community Service)
- Science and Research Affairs Directiorate General
- Research Ethics Directorate

MoSHE

- Dr.Kidest Bobosha (PI)

Addis Ababaw

www.moshe.gov.et info@ethernet.edu.et www.facebook.com/SHE.Ethio

☎ +251-118-721747 ✉ 23976 ፖ.ሣ.ቁ/ CODE 1000

Scanned by CamScanner

የሳይንስና ከፍተኛ ትምህርት ሚኒስቴር
Ministry of Science and Higher Education - Ethiopia

Ref.No. MoSHE//RD/14.1/10116/20

Date: 17 FEB 2020

Armuer Hansen Research Institute (AHRI)
Addis Ababa

Subject: ***Letter of Approval***

The Ministry of Science and Higher Education (MoSHE) via its National Research Ethics Review Committee has reviewed **"Comparing the Effectiveness and Feasibility of a Skincamp Intervention to a Healthcentre Based Intervention as Countries Scale-up Use of Chemoprophylaxis for Leprosy"** project protocol in an expedited manner. We are writing to advise you that MoSHE has granted full approval to the above named project, for a period of **one year (February 17, 2020- February 16, 2021)**..

All your most recently submitted documents have been approved for use in this study. The study should comply with the international and national scientific and ethical standard guidelines. Any change to the approved protocol or consent material must be reviewed and approved through the amendment process prior to its implementation. In addition, any adverse or unanticipated events should be reported within 24-48 hours to MoSHE. Please ensure that you submit biannual progress report to MoSHE once in six months and annual renewal application 30 days prior to the expiry date.

We, therefore, request you as PI and your esteemed organization to ensure the commencement and conduct of the study accordingly and wish for the successful completion of the project.

Sincerely

Solomon Benor Belay (PhD)
Director General for Science and Research Affairs

Cc.

- Office of the State Minister (Sector for Science, Research and Community Service)
- Science and Research Affairs Directiorate General
- Research Ethics Directorate

MoSHE

- Dr.Kidest Bobosha (PI)

Addis Ababaw

www.moshe.gov.et info@ethernet.edu.et www.facebook.com/SHE.Ethio

☎ +251-118-721747 ✉ 23976 ከድ/ CODE 1000

Scanned by CamScanner

REPÚBLICA DE MOÇAMBIQUE
MINISTÉRIO DA SAÚDE
COMITÉ NACIONAL DE BIOÉTICA PARA A SAÚDE
IRB00002657

Exmo. Senhor
Dr. Fernando Mitano
Unilúrio

Ref:342/CNBS/20 **Data 15 de Julho de 2020**

Assunto: Renovação da aprovação do protocolo de estudo intitulado: "***Ensaio de implementação médica sobre triagem cutânea e administração de rifampicina como profilaxia pós-exposição para contactos de pessoas afetadas pela lepra em Murrupula, Meconta e Mogovolas***"

O Comité Nacional de Bioética para a Saúde (CNBS) analisou o pedido de renovação anual da aprovação do protocolo de estudo intitulado: ***"Ensaio de implementação médica sobre triagem cutânea e administração de rifampicina como profilaxia pós-exposição para contactos de pessoas afetadas pela lepra em Murrupula, Meconta e Mogovolas"***, *e sobre* o mesmo o CNBS chegou a seguinte conclusão:

Não havendo nenhum inconveniente de ordem ética que impeça a continuação do estudo, o CNBS dá a autorização.

Todavia, recomenda aos investigadores que mantenham o CNBS informado do decurso do estudo.

A aprovação da renovação tem a validade de um ano, terminando esta a 15 de Julho de 2021. Os investigadores deverão submeter o pedido de renovação da aprovação um mês antes de terminar o prazo.

Sem mais de momento, queiram aceitar as nossas cordiais saudações.

O Presidente

REPÚBLICA DE MOÇAMBIQUE MINISTÉRIO DA SAÚDE COMITÉ NACIONAL DE BIOÉTICA PARA A SAÚDE

Dr. João Fernando Lima Schwalbach

Endereço:
Ministério da Saúde - 2º andar dto
Av. Eduardo Mondlane / Salvador Allende
Maputo - Moçambique

C.Postal: 264
Telefone: +258 82 406 6350
E-mail: cnbsmocambique@gmail.com

REPÚBLICA DE MOÇAMBIQUE
MINISTÉRIO DA SAÚDE
COMITÉ NACIONAL DE BIOÉTICA PARA A SAÚDE
IRB00002657

Exmo. Senhor
Professor Doutor Francisco Mitano
CIBS-Unilúrio

Ref:385/CNBS/19 **Data 16 de Agosto de 2019**

Assunto: Aprovação do Comité Nacional de Bioética para Saúde (CNBS) referente ao protocolo de estudo intitulado: "*Ensaio de implementação médica sobre triagem cutânea e administração de rifampicina como profilaxia pós-exposição para contactos de pessoas afectadas pela Lepra em Morrupula, Meconta e Mogovolas*"

O Comité Nacional de Bioética para Saúde (CNBS) analisou as correcções efectuadas no protocolo de estudo intitulado: "***Ensaio de implementação médica sobre triagem cutânea e administração de rifampicina como profilaxia pós-exposição para contactos de pessoas afectadas pela Lepra em Morrupula, Meconta e Mogovolas***"

Registado no CNBS com o número 31/CNBS/2019, conforme os requisitos da Declaração de Helsínquia.

Não havendo nenhum inconveniente de ordem ética que impeça a realização do estudo, o CNBS dá a sua devida aprovação aos seguintes documentos:

- Protocolo de estudo, *versão S/N de Fevereiro de 2019*
- Consentimento informado, *versão S/N de Fevereiro de 2019*
- Instrumento de recolha de dados, *versão S/N de Fevereiro de 2019*

Todavia, o CNBS informa que:

1- Qualquer alteração a ser introduzida no protocolo, incluindo os seus anexos deve ser submetida ao CNBS para aprovação.
2- A presente aprovação não substitui a autorização administrativa.
3- Não houve declaração de conflitos de interesse por nenhum dos membros do CNBS.
4- A aprovação terá a validade de um ano, terminando esta a 16 de Agosto de 2020. Os investigadores deverão submeter o pedido de renovação da aprovação um mês antes de terminar o prazo.
5- Recomenda-se aos investigadores que mantenham o CNBS informado do decurso do estudo.
6- A lista actualizada dos membros do CNBS esta disponível na secretaria do Comité.

Sem mais do momento, queiram aceitar as nossas mais cordiais saudações.

O Presidente

Dr. João Fernando Lima Schwalbach

Endereço:
Ministério da Saúde - 2º andar dto
Av. Eduardo Mondlane / Salvador Allende
Maputo - Moçambique

C.Postal: 264
Telefone: +258 82 406 6350
E-mail: cnbsmocambique@gmail.com

16 August 2019

Subject: Approval of the National Committee on Bioethics in Health (Comisión Nacional de Bioética en Saúde, CNBS)

Referring to the protocol of the study "Chemoprophylaxis for leprosy: comparing the effectiveness and feasibility of a skin camp intervention to a health centre-based intervention" ("PEP4LEP").

CNBS analyzed the corrections made to the protocol of the studio "PEP4LEP" registered at CNBS with number 31/CNBS/2019. According to the requirements of the Declaration of Helsinki, there are no ethical inconvenience found that impede the realization of the study. Therefore, the CNBS gives its approval to the following documents:

- PEP4LEP Study Protocol, version February 2019
- PEP4LEP Informed Consent Forms, version February 2019
- PEP4LEP Data Collection Forms, version February 2019

PEP4LEP: IMPLEMENTATION TRIAL ON SKIN SCREENING AND RIFAMPICIN AS POST-EXPOSURE PROPHYLAXIS FOR CONTACTS OF PEOPLE AFFECTED BY LEPROSY IN ETHIOPIA, MOZAMBIQUE AND TANZANIA.

FUNDED BY:

This project is part of the EDCTP2 programme supported by the European Union

This project has received funding from the Leprosy Research Initiative (LRI; www.leprosyresearch.org) under LRI Grant number 707.19.58

THE UNITED REPUBLIC OF TANZANIA

National Institute for Medical Research
3 Barack Obama Drive
P.O. Box 9653
11101 Dar es Salaam
Tel: 255 22 2121400
Fax: 255 22 2121360
Email: nimrethics@gmail.com

Ministry of Health, Community Development, Gender, Elderly &Children
University of Dodoma, College of Business Studies and Law
Building No. 11
P.O. Box 743
40478 Dodoma

NIMR/HQ/R.8c/Vol. I /1530

20th July 2020

Dr. Beatrice Mutayoba
Ministry of Health, Community Development,
Gender, Elderly & Children
National TB and Leprosy Programme
P O Box 9083
Dar es Salaam

RE: APPROVAL FOR EXTENSION OF ETHICAL CLEARANCE

This letter is to confirm that your application for extension on the already approved proposal: PEP4LEP: comparing the effectiveness and feasibility of a skin camp intervention to a health center based intervention as countries scale up use of chemoprophylaxis for leprosy. An implementation trial in Mozambique, Ethiopia and Tanzania (Mutayoba B. et al), has been approved.

The extension approval is based on the progress report dated 22nd June, 2020 on the project, Ref. NIMR/HQ/R.8a/Vol. IX/3131, dated 17th June, 2019. Extension approval is valid until 16th June, 2021.

The Principal Investigator must ensure that other conditions of approval remain as per ethical clearance letter. The PI should ensure that progress and final reports are submitted in a timely manner.

Name: Prof. Yunus Daud Mgaya

Signature
CHAIRPERSON
MEDICAL RESEARCH
COORDINATING COMMITTEE

Name: Prof. Abel Nkono Makubi

Signature
CHIEF MEDICAL OFFICER
MINISTRY OF HEALTH, COMMUNITY
DEVELOPMENT, GENDER, ELDERLY
& CHILDREN

THE UNITED REPUBLIC OF TANZANIA

National Institute for Medical Research
3 Barack Obama Drive
P.O. Box 9653
11101 Dar es Salaam
Tel: 255 22 2121400
Fax: 255 22 2121360
E-mail: nimrethics@gmail.com

Ministry of Health, Community Development, Gender, Elderly & Children
University of Dodoma, College of Business Studies and Law
Building No. 11
P.O. Box 743
40478 Dodoma

NIMR/HQ/R.8a/Vol. IX/3131

17th June, 2019

Dr. Beatrice Mutayoba
Ministry of Health, Community Development, Gender, Elderly & Children
National TB and Leprosy Programme
P. O. Box 9083
Dar es Salaam

RE: ETHICAL CLEARANCE CERTIFICATE FOR CONDUCTING MEDICAL RESEARCH IN TANZANIA

This is to certify that the research entitled: PEP4LEP: comparing the effectiveness and feasibility of a skin camp intervention to a health centre based intervention as countries scale up use of chemoprophylaxis for leprosy. An implementation trial in Mozambique, Ethiopia and Tanzania (Mutayoba B. et al), has been granted ethical clearance to be conducted in Tanzania.

The Principal Investigator of the study must ensure that the following conditions are fulfilled:

1. Progress report is submitted to the Ministry of Health, Community Development, Gender, Elderly & Children and the National Institute for Medical Research, Regional and District Medical Officers after every six months.
2. Permission to publish the results is obtained from National Institute for Medical Research.
3. Copies of final publications are made available to the Ministry of Health, Community Development, Gender, Elderly & Children and the National Institute for Medical Research.
4. Any researcher, who contravenes or fails to comply with these conditions, shall be guilty of an offence and shall be liable on conviction to a fine as per NIMR Act No. 23 of 1979, PART III Section 10(2).
5. Sites: Lindi District Council in Lindi region, Morogoro District Council and Mvomero District Council in Morogoro region.

Approval is valid for one year: 17th June 2019 to 16th June 2020.

Name: Prof. Yunus Daud Mgaya

Signature
CHAIRPERSON
MEDICAL RESEARCH COORDINATING COMMITTEE

Name: Prof. Muhammad Bakari Kambi

Signature
CHIEF MEDICAL OFFICER
MINISTRY OF HEALTH, COMMUNITY DEVELOPMENT, GENDER, ELDERLY & CHILDREN

CC: Director, Health Services -TAMISEMI, Dodoma
RMO of Lindi and Morogoro regions
DMO/DED of respective districts

Erasmus MC
Universitair Medisch Centrum Rotterdam

Medische Ethische Toetsings Commissie
Erasmus MC
www.erasmusmc.nl/commissies/metc/

Prof.dr. J.H. Richardus
Afdeling iMGZ Maatsch.Gezondheidszorg Algemeen
Kamer NA-2322
Erasmus MC

Doorkiesnummer +31 10 7033625/34428
Kamernummer Ae-337
E-mail metc@erasmusmc.nl
Ons kenmerk WT/aj/MEC-2019-0130
Datum 11 april 2019

Betreft: MEC-2019-0130, Besluit onderzoek is niet WMO-plichtig
Multicenter, Erasmus MC initieert

Protocol titel:
'Chemoprophylaxis for leprosy: comparing the effectiveness and feasibility of a skin camp intervention to a health centre based intervention. An implementation trial in Mozambique, Ethiopia and Tanzania'

Protocol versie 3.2 d.d. 28 december 2018

Geachte heer Richardus,

De Medisch Ethische Toetsings Commissie Erasmus MC heeft het door u ingediende bovenvermeld onderzoeksvoorstel, volledig conform de eisen van de METC ontvangen op 24 februari 2019 ter beoordeling van de WMO-plichtigheid.

Het dagelijks bestuur van de commissie heeft beoordeeld of dit onderzoek al dan niet binnen de reikwijdte van de WMO valt. In verband hiermee is het dagelijks bestuur tot de conclusie gekomen dat:

- de proefpersonen wel aan een handeling worden onderworpen of er wordt hen een gedragswijze opgelegd.
- de proefpersonen niet aan een handeling worden onderworpen en er wordt hen geen gedragswijze opgelegd, beide zoals bedoeld in de WMO.

Omdat aan één van beide voorwaarden voor WMO-plichtigheid niet is voldaan, heeft het dagelijks bestuur van de commissie d.d. 2 april 2019 besloten dat bovenvermeld onderzoek niet WMO-plichtig is. U mag dit onderzoek uitvoeren in het Erasmus MC en u kunt de resultaten te zijner tijd voor publicatie aanbieden aan een wetenschappelijk tijdschrift.

De commissie attendeert u op de volgende punten

- De commissie heeft alleen de WMO-plichtigheid beoordeeld. Er heeft verder geen inhoudelijke toets van het onderzoek plaatsgevonden.

- U en uw afdeling zijn verantwoordelijk voor de correcte uitvoering van het onderzoek volgens de geldende wet– en regelgeving. Hierbij vestigen wij uw aandacht op het volgende:

Postadres
Postbus 2040
3000 CA Rotterdam

Bezoekadres
Dr. Molenwaterplein 40
3015 CD Rotterdam

Parkeergarage
Westzeedijk 361
3015 AA Rotterdam

Voorzitters
Prof.dr. H.W. Tilanus
Prof.dr. H.J. Metselaar

Secretarissen
Mw. mr. C.P. Bron-van Vliet
Mw.drs. N. Loekabino
Mw.dr. F.M. Spoelstra
Mw.ing. W.C.M. Tielemans

Secretaresses
Mw. A. de Jong
Mw. S. Sneevliet

Het secretariaat is geopend van maandag tot en met vrijdag van 08.30 tot 17.00 uur

Erasmus MC
Universitair Medisch Centrum Rotterdam

- Voor prospectief onderzoek, waarbij gegevens van proefpersonen worden verzameld en verwerkt, is toestemming van de proefpersonen nodig. U vindt een voorbeeld patiënteninformatie- en toestemmingsformulier voor niet WMO-plichtig onderzoek op de site van de METC. (www.erasmusmc.nl /commissies/metc/)

- Voor retrospectief onderzoek, waarbij gegevens van proefpersonen gecodeerd worden verzameld en verwerkt is toestemming van de proefpersonen nodig. U vindt een voorbeeld patiënteninformatie- en toestemmingsformulier voor niet WMO-plichtig onderzoek op de site van de METC (www.erasmusmc.nl /commissies/metc/).
  (Bij retrospectief *anoniem* onderzoek is toestemming niet verplicht, hierbij zijn de gegevens nooit meer herleidbaar tot de proefpersonen.)

- Wanneer in een onderzoek gegevens worden verzameld van proefpersonen, dient hiermee correct te worden omgegaan zoals bepaald in de Gedragscode Gezondheidsonderzoek (Code Goed Gedrag), het Privacy Reglement Erasmus MC en de Algemene Verordening Gegevensbescherming (AVG). U vindt hierover meer informatie op de website van de METC (www.erasmusmc.nl /commissies/metc/) en op de website van FEDERA (www.federa.org).

- Wanneer in een onderzoek (lichaams)materiaal van proefpersonen wordt verzameld en verwerkt dient hiermee correct te worden omgegaan zoals bepaald in de Code Goed Gebruik. U vindt hierover meer informatie op de website van FEDERA (www.federa.org).

- Vergunningplichtig bevolkingsonderzoek moet worden ingediend bij de Commissie Bevolkingsonderzoek ter toetsing conform de Wet bevolkingsonderzoek. U vindt hierover meer informatie op de website van de CCMO (www.ccmo.nl).

- Niet WMO-plichtig Fase IV Geneesmiddelen onderzoek dat wordt geïnitieerd door de farmaceutische industrie dient te worden getoetst en uitgevoerd conform de Gedragscode Geneesmiddelenreclame. U vindt hierover meer informatie op de site van de stichting code geneesmiddelen reclame (www.cgr.nl).

- Amendementen en/of addenda bij dit onderzoek dienen aan de commissie ter beoordeling te worden voorgelegd zodat kan worden beoordeeld of het onderzoek nog steeds buiten de reikwijdte van de WMO blijft, of dat er door het amendement/addendum sprake is van WMO-plichtig onderzoek.

- Onderzoekers in het Erasmus MC dienen zich te houden aan de research codes, zoals vastgelegd in de uitgave 'Research Codes' van de afdeling Onderzoeksbeleid, te vinden op Intranet.

- Voor ethische toetsing van Onderwijsonderzoek verwijst de commissie u naar de website van de NVMO-ERB (www.nvmo.nl).

Pagina 3/3
Ons kenmerk WT/aj/MEC-2019-0130
Datum 11 april 2019

Erasmus MC
Universitair Medisch Centrum Rotterdam

- De commissie verzoekt u haar op de hoogte te brengen van de volgende gegevens betreffende dit onderzoek:
    - Startdatum (datum inclusie eerste proefpersoon) en/of start gegevens onderzoek
    - einddatum (datum stop studie laatste proefpersoon) en/of stop gegevens onderzoek
    - publicaties en/of eindrapport

Wanneer u vragen heeft over het opzetten, financieren, of uitvoeren van wetenschappelijk onderzoek, kunt u terecht bij het Consultatiecentrum Patiëntgebonden Onderzoek (CPO) voor advies en hulp. Het CPO organiseert ook meerdere keren per jaar de BROK cursus (Basiscursus Regelgeving en Organisatie van Klinisch Onderzoek), die door de commissie van harte wordt aanbevolen. Het volgen van de BROK cursus is, conform landelijke afspraken, alleen verplicht bij WMO-plichtig onderzoek. Voor informatie over de BROK-cursusdata kunt u contact opnemen met het Congresbureau, intern tel.nr. 43584.

Op de site van de METC kunt u links terugvinden naar de hierboven vermelde wet- en regelgeving. Wanneer u vragen heeft over dit METC besluit, kunt u contact opnemen met het secretariaat van de METC.

Met vriendelijke groet,
namens de Medisch Ethische Toetsings Commissie Erasmus MC,

i.o.

Mw.ing. W.C.M. Tielemans
Secretaris

To whom it may concern,

The Daily Board of the Medical Ethics Committee Erasmus MC (hereafter the Committee) of Rotterdam, The Netherlands, has reviewed the above mentioned research proposal. As a result of this review, the Committee informs you that the rules laid down in the Medical Research Involving Human Subjects Act (also known by its Dutch abbreviation WMO), do not apply to this research proposal.

Please indicate the above MEC-number in every correspondence on this study

Yours sincerely,
On behalf of the Medical Ethics Committee Erasmus MC,

i.o.

Mrs. W.C.M. Tielemans, BASc
Secretary of the Committee

STANDARD PROTOCOL ITEMS: RECOMMENDATIONS FOR INTERVENTIONAL TRIALS

SPIRIT 2013 Checklist: Recommended items to address in a clinical trial protocol and related documents*

Section/item	ItemNo	Description	BMJ Open
**Administrative information**			
Title	1	Descriptive title identifying the study design, population, interventions, and, if applicable, trial acronym	Page 1
Trial registration	2a	Trial identifier and registry name. If not yet registered, name of intended registry	Page 1
	2b	All items from the World Health Organization Trial Registration Data Set	N/A
Protocol version	3	Date and version identifier	Page 1, Trial registration
Funding	4	Sources and types of financial, material, and other support	Page 14, Funding
Roles and responsibilitie s	5a	Names, affiliations, and roles of protocol contributors	Page 1<br>Page 14, Authors' contributions
	5b	Name and contact information for the trial sponsor	Page 1
	5c	Role of study sponsor and funders, if any, in study design; collection, management, analysis, and interpretation of data; writing of the report; and the decision to submit the report for publication, including whether they will have ultimate authority over any of these activities	Page 11, Availability of data and materials<br>Page 3, Objectives (details on study consortium)<br>Page 14, Funding
	5d	Composition, roles, and responsibilities of the coordinating centre, steering committee, endpoint adjudication committee, data management team, and other individuals or groups overseeing the trial, if applicable (see Item 21a for data monitoring committee)	Page 11, Data collection and management<br>Page 12, Ethics, paragraph 5
**Introduction**			

For peer review only - http://bmjopen.bmj.com/site/about/guidelines.xhtml

Background and rationale	6a	Description of research question and justification for undertaking the trial, including summary of relevant studies (published and unpublished) examining benefits and harms for each intervention	Page 3, Objectives
	6b	Explanation for choice of comparators	Page 8, Outcomes
Objectives	7	Specific objectives or hypotheses	Page 3, Objectives<br>Page 9, Table 3
Trial design	8	Description of trial design including type of trial (eg, parallel group, crossover, factorial, single group), allocation ratio, and framework (eg, superiority, equivalence, noninferiority, exploratory)	Page 6, Study Design<br>Page 7, Figure 3
**Methods: Participants, interventions, and outcomes**			
Study setting	9	Description of study settings (eg, community clinic, academic hospital) and list of countries where data will be collected. Reference to where list of study sites can be obtained	Page 4, Study setting
Eligibility criteria	10	Inclusion and exclusion criteria for participants. If applicable, eligibility criteria for study centres and individuals who will perform the interventions (eg, surgeons, psychotherapists)	Page 4, Participants and eligibility criteria
Interventions	11a	Interventions for each group with sufficient detail to allow replication, including how and when they will be administered	Page 6, Community based skin camp intervention<br>Page 6, Health center-based intervention for household contacts
	11b	Criteria for discontinuing or modifying allocated interventions for a given trial participant (eg, drug dose change in response to harms, participant request, or improving/worsening disease)	Page 4, Participants and eligibility criteria<br>Page 5, Table 1
	11c	Strategies to improve adherence to intervention protocols, and any procedures for monitoring adherence (eg, drug tablet return, laboratory tests)	N/A
	11d	Relevant concomitant care and interventions that are permitted or prohibited during the trial	Page 4, Participants and eligibility criteria<br>Page 5, Table 1

For peer review only - http://bmjopen.bmj.com/site/about/guidelines.xhtml

Outcomes	12	Primary, secondary, and other outcomes, including the specific measurement variable (eg, systolic blood pressure), analysis metric (eg, change from baseline, final value, time to event), method of aggregation (eg, median, proportion), and time point for each outcome. Explanation of the clinical relevance of chosen efficacy and harm outcomes is strongly recommended	Page 8, Outcomes
Participant timeline	13	Time schedule of enrolment, interventions (including any run-ins and washouts), assessments, and visits for participants. A schematic diagram is highly recommended (see Figure)	Page 4, Study setting<br>Page 6, Study Design<br>Page 7, Figure 3
Sample size	14	Estimated number of participants needed to achieve study objectives and how it was determined, including clinical and statistical assumptions supporting any sample size calculations	Page 10, Sample size
Recruitment	15	Strategies for achieving adequate participant enrolment to reach target sample size	Page 10, Sample size

Methods: Assignment of interventions (for controlled trials)

Allocation:

Sequence generation	16a	Method of generating the allocation sequence (eg, computer-generated random numbers), and list of any factors for stratification. To reduce predictability of a random sequence, details of any planned restriction (eg, blocking) should be provided in a separate document that is unavailable to those who enrol participants or assign interventions	Page 11, Data collection and management, paragraph 3
Allocation concealme nt mechanis m	16b	Mechanism of implementing the allocation sequence (eg, central telephone; sequentially numbered, opaque, sealed envelopes), describing any steps to conceal the sequence until interventions are assigned	Page 11, Randomization

For peer review only - http://bmjopen.bmj.com/site/about/guidelines.xhtml

Implementation	16c	Who will generate the allocation sequence, who will enrol participants, and who will assign participants to interventions	Page 11, Data collection and management, paragraph 3
Blinding (masking)	17a	Who will be blinded after assignment to interventions (eg, trial participants, care providers, outcome assessors, data analysts), and how	N/A
	17b	If blinded, circumstances under which unblinding is permissible, and procedure for revealing a participant's allocated intervention during the trial	N/A
**Methods: Data collection, management, and analysis**			
Data collection methods	18a	Plans for assessment and collection of outcome, baseline, and other trial data, including any related processes to promote data quality (eg, duplicate measurements, training of assessors) and a description of study instruments (eg, questionnaires, laboratory tests) along with their reliability and validity, if known. Reference to where data collection forms can be found, if not in the protocol	Page 11, Data collection and management
	18b	Plans to promote participant retention and complete follow-up, including list of any outcome data to be collected for participants who discontinue or deviate from intervention protocols	Page 11, Data collection and management
Data management	19	Plans for data entry, coding, security, and storage, including any related processes to promote data quality (eg, double data entry; range checks for data values). Reference to where details of data management procedures can be found, if not in the protocol	Page 11, Data collection and management
Statistical methods	20a	Statistical methods for analysing primary and secondary outcomes. Reference to where other details of the statistical analysis plan can be found, if not in the protocol	Page 11, Data analysis
	20b	Methods for any additional analyses (eg, subgroup and adjusted analyses)	Page 11, Data analysis

For peer review only - http://bmjopen.bmj.com/site/about/guidelines.xhtml

	20c	Definition of analysis population relating to protocol non-adherence (eg, as randomised analysis), and any statistical methods to handle missing data (eg, multiple imputation)	Page 11, Data analysis
**Methods: Monitoring**			
Data monitoring	21a	Composition of data monitoring committee (DMC); summary of its role and reporting structure; statement of whether it is independent from the sponsor and competing interests; and reference to where further details about its charter can be found, if not in the protocol. Alternatively, an explanation of why a DMC is not needed	Page 12, Ethics, paragraph 5
	21b	Description of any interim analyses and stopping guidelines, including who will have access to these interim results and make the final decision to terminate the trial	N/A
Harms	22	Plans for collecting, assessing, reporting, and managing solicited and spontaneously reported adverse events and other unintended effects of trial interventions or trial conduct	Page 12, Ethics, paragraph 2
Auditing	23	Frequency and procedures for auditing trial conduct, if any, and whether the process will be independent from investigators and the sponsor	Page 12, Ethics, paragraph 5
**Ethics and dissemination**			
Research ethics approval	24	Plans for seeking research ethics committee/institutional review board (REC/IRB) approval	Page 12, Ethics, paragraph 1
Protocol amendments	25	Plans for communicating important protocol modifications (eg, changes to eligibility criteria, outcomes, analyses) to relevant parties (eg, investigators, REC/IRBs, trial participants, trial registries, journals, regulators)	Page 12, Ethics, paragraph 1
Consent or assent	26a	Who will obtain informed consent or assent from potential trial participants or authorised surrogates, and how (see Item 32)	Page 12, Ethics, paragraph 2

only

For peer review only - http://bmjopen.bmj.com/site/about/guidelines.xhtml

	26b	Additional consent provisions for collection and use of participant data and biological specimens in ancillary studies, if applicable	N/A
Confidentialit y	27	How personal information about potential and enrolled participants will be collected, shared, and maintained in order to protect confidentiality before, during, and after the trial	Page 11, Data collection and management, paragraph 3
Declaration of interests	28	Financial and other competing interests for principal investigators for the overall trial and each study site	Page 14, Competing interests
Access to data	29	Statement of who will have access to the final trial dataset, and disclosure of contractual agreements that limit such access for investigators	Page 11, Availability of data and materials
Ancillary and post-trial care	30	Provisions, if any, for ancillary and post-trial care, and for compensation to those who suffer harm from trial participation	N/A
Disseminatio n policy	31a	Plans for investigators and sponsor to communicate trial results to participants, healthcare professionals, the public, and other relevant groups (eg, via publication, reporting in results databases, or other data sharing arrangements), including any publication restrictions	Page 11, Availability of data and materials
	31b	Authorship eligibility guidelines and any intended use of professional writers	N/A
	31c	Plans, if any, for granting public access to the full protocol, participant-level dataset, and statistical code	Page 11, Availability of data and materials
**Appendices**			
Informed consent materials	32	Model consent form and other related documentation given to participants and authorised surrogates	Added to supplementary materials of submission
Biological specimens	33	Plans for collection, laboratory evaluation, and storage of biological specimens for genetic or molecular analysis in the current trial and for future use in ancillary studies, if applicable	N/A

*It is strongly recommended that this checklist be read in conjunction with the SPIRIT 2013 Explanation & Elaboration for important clarification on the items. Amendments to the

For peer review only - http://bmjopen.bmj.com/site/about/guidelines.xhtml

protocol should be tracked and dated. The SPIRIT checklist is copyrighted by the SPIRIT Group under the Creative Commons "Attribution-NonCommercial-NoDerivs 3.0 Unported" license.

For peer review only

For peer review only - http://bmjopen.bmj.com/site/about/guidelines.xhtml

BMJ Open

The PEP4LEP study protocol: Integrated skin screening and SDR-PEP administration for leprosy prevention. Comparing the effectiveness and feasibility of a community-based intervention to a health center-based intervention in Ethiopia, Mozambique and Tanzania

Journal:	*BMJ Open*
Manuscript ID	bmjopen-2020-046125.R1
Article Type:	Protocol
Date Submitted by the Author:	28-Jun-2021
Complete List of Authors:	Schoenmakers, Anne; NLR<br>Hambridge, Thomas; Erasmus Medical Center<br>van Wijk, Robin; NLR<br>Kasang, Christa; DAHW<br>Richardus, Jan Hendrik; Erasmus Medical Center, Department of Public Health<br>Bobosha, Kidist; Armauer Hansen Research Institute<br>Mitano, Fernando; Lurio University<br>Mshana, Stephen E.; Catholic University of Health and Allied Sciences, Department of Microbiology and Immunology<br>Mamo, Ephrem; Armauer Hansen Research Institute<br>Marega, Abdoulaye; Lurio University<br>Mwageni, Nelly; Catholic University of Health and Allied Sciences, Department of Microbiology and Immunology<br>Letta, Taye; Ethiopia Ministry of Health<br>Muloliwa, Artur; Ministry of Health, Mozambique<br>Kamara, Vedastus; Ministry of Health, Tanzania<br>Eman, Ahmed; DAHW, Ethiopia<br>Raimundo, Litos; NLR, Mozambique<br>Njako, Blasdus; DAHW, Tanzania<br>Mieras, Liesbeth; NLR
**Primary Subject Heading**:	Infectious diseases
Secondary Subject Heading:	Public health, Dermatology
Keywords:	Public health < INFECTIOUS DISEASES, Infectious diseases & infestations < DERMATOLOGY, Tropical medicine < INFECTIOUS DISEASES

SCHOLARONE™
Manuscripts

I, the Submitting Author has the right to grant and does grant on behalf of all authors of the Work (as defined in the below author licence), an exclusive licence and/or a non-exclusive licence for contributions from authors who are: i) UK Crown employees; ii) where BMJ has agreed a CC-BY licence shall apply, and/or iii) in accordance with the terms applicable for US Federal Government officers or employees acting as part of their official duties; on a worldwide, perpetual, irrevocable, royalty-free basis to BMJ Publishing Group Ltd ("BMJ") its licensees and where the relevant Journal is co-owned by BMJ to the co-owners of the Journal, to publish the Work in this journal and any other BMJ products and to exploit all rights, as set out in our licence.

The Submitting Author accepts and understands that any supply made under these terms is made by BMJ to the Submitting Author unless you are acting as an employee on behalf of your employer or a postgraduate student of an affiliated institution which is paying any applicable article publishing charge ("APC") for Open Access articles. Where the Submitting Author wishes to make the Work available on an Open Access basis (and intends to pay the relevant APC), the terms of reuse of such Open Access shall be governed by a Creative Commons licence – details of these licences and which Creative Commons licence will apply to this Work are set out in our licence referred to above.

Other than as permitted in any relevant BMJ Author's Self Archiving Policies, I confirm this Work has not been accepted for publication elsewhere, is not being considered for publication elsewhere and does not duplicate material already published. I confirm all authors consent to publication of this Work and authorise the granting of this licence.

For peer review only

The PEP4LEP study protocol: Integrated skin screening and SDR-PEP administration for leprosy prevention. Comparing the effectiveness and feasibility of a community-based intervention to a health center-based intervention in Ethiopia, Mozambique and Tanzania

A. Schoenmakers[1*], T. Hambridge[2*], R. van Wijk[1], C. Kasang[3], J.H. Richardus[2], K. Bobosha[4], F. Mitano[5], S.E. Mshana[6], E. Mamo[4], A. Marega[5], N. Mwageni[6], Taye Letta[7], A. M. Muloliwa[8], D. V. Kamara[9], A. M. Eman[10], L. Raimundo[11], B. Njako[12], L. Mieras[1]

[1] NLR, The Netherlands; [2] Erasmus MC, University Medical Center Rotterdam, The Netherlands; [3] DAHW, Germany; [4] AHRI, Ethiopia; [5] Universidade Lúrio, Mozambique; [6] CUHAS University, Tanzania, [7] Ministry of Health, Ethiopia; [8] Ministry of Health, Mozambique; [9] Ministry of Health, Tanzania; [10] GLRA, Ethiopia; [11] NLR, Mozambique, [12] GLRA, Tanzania

Both authors contributed equally to this article.

Correspondence: t.hambridge@erasmusmc.nl

For peer review only

Abstract

Introduction

Leprosy, or Hansen's disease, remains a cause of preventable disability. Early detection, treatment and prevention are key to reducing transmission. Post-exposure prophylaxis with single-dose rifampicin (SDR-PEP) reduces the risk of developing leprosy when administered to screened contacts of patients. This has been adopted in the World Health Organization (WHO) leprosy guidelines. The PEP4LEP study aims to determine the most effective and feasible method of screening people at risk of developing leprosy and administering chemoprophylaxis to contribute to interrupting transmission.

Methods and analysis

PEP4LEP is a cluster-randomized implementation trial comparing two interventions of integrated skin screening combined with SDR-PEP distribution to contacts of leprosy patients in Ethiopia, Mozambique, and Tanzania. One intervention is community-based, using skin camps to screen approximately 100 community contacts per leprosy patient and to administer SDR-PEP when eligible. The other intervention is health center-based, inviting household contacts of leprosy patients to be screened in a local health center and subsequently receive SDR-PEP when eligible. The mobile health (mHealth) tool SkinApp will support health workers' capacity in integrated skin screening. The effectiveness of both interventions will be compared by assessing

the rate of leprosy patients detected and case detection delay in months, as well as feasibility in terms of cost-effectiveness and acceptability.

Ethics and dissemination

Ethical approval was obtained from the national ethical committees of Ethiopia (MoSHE), Mozambique (CNBS) and Tanzania (NIMR/ MoHCDEC). Study results will be published open access in peer-reviewed journals, providing evidence for the implementation of innovative leprosy screening methods and chemoprophylaxis to policymakers.

Trial registration: The PEP4LEP project is registered at the Netherlands Trial Register (NTR), receiving trial registration number NL7294 (NTR7503), registration date September 10, 2018.

Keywords: leprosy, Hansen's disease, NTD, chemoprophylaxis, prevention, skin screening, case detection, single dose rifampicin, SDR-PEP, post-exposure prophylaxis, detection delay, skin camps, Ethiopia, Mozambique, Tanzania, Africa, feasibility, acceptability, cost-effectiveness, mHealth, eHealth

Article Summary

Strengths and Limitations

- In both interventions, a combination of screening contacts and providing SDR-PEP will be used according to the World Health Organization's guidelines to reduce the contacts' risk of developing leprosy
- An integrated skin screening approach will be used in which multiple diseases can be detected and treated at once, overcoming the often negative associations with leprosy
- The SkinApp will be used as a mHealth tool to support peripheral health workers in recognizing and treating signs and symptoms of skin diseases; while innovative and potentially increasing capacity, the accuracy and reproducibility of this tool awaits further investigation
- Since the epidemiological impact on new case detection rate will not become apparent within the study duration, the primary outcome measures are case detection delay, number of contacts diagnosed with leprosy and number of contacts who received chemoprophylaxis

For peer review only - http://bmjopen.bmj.com/site/about/guidelines.xhtml

- Because difficulties in recalling the first signs and symptoms are expected to increase over a longer duration of the disease, only recently diagnosed index patients will be included in this study to establish case detection delay

Introduction

Leprosy, or Hansen's disease, is a communicable disease caused by *Mycobacterium leprae* that is still a public health problem in many countries. It is formally recognized by the World Health Organization (WHO) as a neglected tropical disease (NTD).[1] The annual reported number of newly detected leprosy patients was 202,185 in 2019.[2] If left untreated, leprosy potentially results in disability, which can have severe consequences such as stigma and poverty.[3] Leprosy has a long and variable incubation time, ranging from 2 to 20 years, during which it is assumed that transmission can take place.[4] The risk of developing leprosy is higher in household contacts and neighbors of patients than it is in the general community.[5] Moet et al. demonstrated that physical and genetic distance were independently associated with the risk of a contact developing leprosy.[6] According to the WHO, contract tracing should be offered to a person who has been in contact with an untreated leprosy index case for at least 20 hours per week during at least 3 months in the previous year.[4,7,8] An index case is defined as a person diagnosed with leprosy for the first time.[7]

The WHO has provided multidrug therapy (MDT) free of charge to all leprosy patients since 1995.[9] However, to overcome ongoing transmission in high-endemic areas, innovative measures are needed.[8,10] In 2008, a large randomized controlled trial in Bangladesh (Chemoprophylaxis of Leprosy study, COLEP) demonstrated that a single dose of rifampicin (SDR) given to contacts of newly diagnosed leprosy patients is effective in reducing the risk of leprosy by 57% (95% CI: 24–75%).[11] SDR-PEP was found to be cost-effective in Bangladesh.[12] In the Leprosy Post-Exposure Prophylaxis (LPEP) program, SDR-PEP was implemented in areas representing various health systems across three continents and eight countries, to evaluate the feasibility, effectiveness and impact.[13] The implementation of SDR-PEP within the routine leprosy control programs was proven to be safe and generally well accepted. Based on the LPEP program and a microsimulation leprosy model (SIMCOLEP), SDR-PEP was also found to be cost-effective in India.[14] The concern that SDR-PEP could lead to increased rifampicin resistance in other diseases, such as tuberculosis (TB), was considered in an expert consultation that concluded that SDR-PEP given to contacts of leprosy patients, in the absence of symptoms of active TB, poses a negligible risk of generating resistance in *Mycobacterium tuberculosis* in individuals and in populations.[15] In 2018, SDR-PEP was included in the WHO "Guidelines for the Diagnosis,

For peer review only - http://bmjopen.bmj.com/site/about/guidelines.xhtml

Treatment and Prevention of Leprosy". Once contact tracing has been established, SDR-PEP can be included into the routines of leprosy control programmes with minimal additional efforts and costs.[7,16]

Skin screening is an important detection strategy for skin-NTDs such as leprosy, and is recommended to be embedded in leprosy programmes.[1,7,17,18] Screening for multiple skin diseases at once (integrated or common skin screening) is promoted by WHO.[1,8,19,20] Integration is considered to increase effectiveness and efficiency by minimizing costs and expanding intervention coverage.[19,21] An important obstacle for integrated skin screening is the scarcity of dermatologists in many areas with a high skin NTD endemicity.[22] In sub-Saharan Africa, the situation is critical, with approximately 1 dermatologist per 500,000–1 million inhabitants and even larger shortages in Mozambique and Tanzania according to field reports from PEP4LEP consortium members.[23,24] According to the WHO, community health workers (CHWs) and village volunteers can play a role in screening for skin diseases, but improved knowledge, capacity, and motivation of health workers and community volunteers is essential.[17,19,25–29] As both integrated skin screening for NTDs and SDR-PEP against leprosy are promoted by the WHO, additional implementation studies are necessary to establish whether a combined intervention is acceptable, feasible, and cost-effective in leprosy endemic areas.[1,4,8,19]

Objectives

The PEP4LEP project is a collaboration among study consortium members in five countries in sub-Saharan Africa and the European Union (EU) (Figure 1). The overall aim of this cluster-randomized implementation trial is to contribute to the interruption of *M. leprae* transmission by identifying the most effective and feasible method of screening people at risk of developing leprosy and by administering post-exposure chemoprophylaxis in Ethiopia, Mozambique, and Tanzania. The primary study objectives are to compare the effectiveness and feasibility of a community-based screening and prophylaxis (skin camp) intervention with a health center-based screening and prophylaxis intervention solely for household contacts of a leprosy patient. The case detection delay will be the primary outcome measure to assess effectiveness. Additional objectives are to assess the cost-effectiveness, acceptability and health workers' capacity regarding the integrated skin diseases approach and the use of the supportive mobile health (mHealth) tool SkinApp.[30,31]

Figure 1. PEP4LEP Project organization chart

For peer review only - http://bmjopen.bmj.com/site/about/guidelines.xhtml

Methods and analysis

Study setting

This study will take place in three countries in sub-Saharan Africa: Ethiopia, Mozambique, and Tanzania. The three countries differ socioculturally and in the endemicity for NTDs like leprosy (Figure 2).[2] Districts within these countries were purposefully chosen because of endemicity and the focal distribution of reported leprosy cases. In Ethiopia, three endemic districts are located in East Hararghe Zone (Oromiya region): Girawa, Jarso, and Midega. In Mozambique, the included districts are located in Nampula province: Meconta, Mogovolas, and Murrupula. The Tanzanian districts are Lindi in Lindi Region and Morogoro and Mvomero in Morogoro Region. The original overall study period was October 2018 until January 2023, with an estimated duration of 2.5–3 years for the inclusion of leprosy patients and their contacts. A study extension is expected due to the impact of COVID-19.

Figure 2. PEP4LEP countries' leprosy incidence in 2019 according to the World Health Organization (2020)[2]

Participants and eligibility criteria

Leprosy patients enrolled in the PEP4LEP study are referred to as "index patients". These patients derived from the leprosy programme registries, and preferably diagnosed up to 6 months prior to inclusion to prevent recall problems when assessing the delay in case detection.[32] The inclusion and exclusion criteria for index patients and contacts are summarized in Table 1 and are based on the WHO guidelines and the LPEP program.[4,13] Following the emergence of the Coronavirus Disease 2019 (COVID-19) pandemic, a suspicion of a COVID-19 infection was added as contact exclusion criteria for this study, as physical distancing cannot be guarded when performing skin screening.[33–36] Index patients with suspected COVID-19 can still be included after they have been tested negative and are symptom-free for at least 2 weeks.[33–35]

The target population for the feasibility component of this study as well as the other research objectives, consists of various stakeholders, including: (index) patients, household contacts, community contacts, community leaders, health workers, community health volunteers and health policy decision makers. If applicable, contacts refusing to take SDR-PEP but who are willing to

For peer review only - http://bmjopen.bmj.com/site/about/guidelines.xhtml

participate in the qualitative study component will also be included in the project, contributing to the acceptability component of the study.

The exclusion criterium for these stakeholders is refusal to provide informed consent to participate.

Table 1. PEP4LEP eligibility criteria patients and contacts [4,7,13]

	Index patients	Contacts
**Inclusion criteria**	• Consent to participate in the PEP4LEP project<br>• Diagnosed with leprosy (preferred maximum of 6 months prior to inclusion)[32]<br>• Residence in the PEP4LEP districts for ≥3 months prior to the date of diagnosis<br>• Index patient has started MDT<br>• Community-based skin camp intervention: Leprosy patient gives permission for the set-up of a skin camp in his/her community (sharing their leprosy diagnosis with their contacts is not needed)<br>• Health center-based household screening intervention: Leprosy patient with household contacts, and who is willing to inform these contacts about PEP4LEP	• Consent to participate in the PEP4LEP project<br>• Community-based skin camp intervention: Community contact (living in the 20 closest houses to the index-patient) for ≥3 months<br>• Health center-based household screening intervention: Contact which is a household member of the index patient for ≥3 months, visiting the screening health center ≤3 months after the index patient was included

For peer review only

For peer review only - http://bmjopen.bmj.com/site/about/guidelines.xhtml

**Exclusion criteria**	• Index patient or parents/legal guardians unable to understand the purpose and risks of participating in the PEP4LEP study	• Contact or parents/legal guardians unable to understand the purpose and risks of participating in the PEP4LEP study<br>• Age <2 years and/or <10 kg of weight*<br>• Pregnancy*<br>• Receiving or having received rifampicin for any reason in the last 2 years<br>• Known allergy to rifampicin<br>• History of liver or renal disorders<br>• Individuals with leprosy and those who have possible signs and/or symptoms of leprosy (e.g., leprosy-like skin lesions or nerve manifestations) until their disease status has been clarified[37]**<br>• Individuals with possible signs and/or symptoms of TB (cough for more than two weeks or cough in known HIV/AIDS patients, night sweats, unexplained fever, weight loss) until their disease status has been clarified[37]***<br>• Individuals with possible signs and/or symptoms of COVID-19 (self-assessed temperature ≥38°C, respiratory or cold-like symptoms, sudden loss of smell/taste) or possible contact with a COVID-19 patient in the past 14 days.[33–36]***

* A voucher will be given for repeated skin screening and SDR-PEP. This can be used in a PEP4LEP affiliated health center when this person becomes eligible (e.g., after giving birth).
** If referral was needed and no leprosy is detected, repeated skin screening and SDR-PEP can be provided in a PEP4LEP affiliated health center.

For peer review only - http://bmjopen.bmj.com/site/about/guidelines.xhtml

*** Skin screening and SDR-PEP can only be provided in a PEP4LEP affiliated health center after the contact is tested negative for COVID-19/TB (according to national guidelines).[33–36]
Abbreviations: COVID-19: Coronavirus Disease 2019; MDT: multidrug therapy; SDR-PEP: single-dose rifampicin post-exposure prophylaxis; TB: tuberculosis

Study design

The study is a two-arm, cluster-randomized implementation trial (Figure 3). One intervention is community-based, using skin camps to screen approximately 100 community contacts (household members and neighbors) of a leprosy index patient and to provide them with SDR-PEP when eligible. The second intervention is health center-based, inviting the household contacts of an index patient to be screened and given SDR-PEP when eligible.

Community-based skin camp intervention

A skin camp will be organized when a leprosy patient is diagnosed by inviting approximately 100 people from the same community (Table 1) living in the surrounding area (field definition: inhabitants from the 20 closest houses). Community contacts from outside of the 20 closest households who attend a skin camp can still receive skin screening or referral, but will not be given SDR-PEP. Health camps are designed to bring specialized care closer to the community, thus expanding access.[38] Besides preventive and curative treatment, these camps often also play a significant role to create awareness.[39] Community "skin health camps" have been proposed as an effective way to screen for leprosy and other NTDs.[7,40] Skin camps are organized at the community level and in close collaboration with community leaders and local organizations.[38,41] In a skin camp, health staff screen individuals for skin diseases and then treat or refer patients if necessary. Assistance from a dermatologist (or, if none available, a senior health staff member with sufficient dermatology experience) is vital.[42] A key advantage of this community intervention is that the identity of the person affected by leprosy can be protected since no individual disease disclosure is needed. This non-disclosure approach is of utmost importance, as people affected by leprosy are often stigmatized and discriminated against and are therefore reluctant to share their disease status.[43–45] Moreover, including a wider group of contacts and using an integrated skin diseases approach may overcome the frequently negative associations with leprosy that can prevent people from participating in a leprosy-related intervention.[19] Including approximately 100 contacts per identified leprosy patient in the PEP4LEP skin camps is in-line with the risk profiles of the contact groups and is operationally manageable conduct within 1-2 days, also when using time slots to prevent crowding taking COVID-19 into consideration.[6,13,34,36,39,40,46–48]

Health center-based intervention for household contacts

In the second intervention, newly detected leprosy patients will be asked to invite their household

For peer review only - http://bmjopen.bmj.com/site/about/guidelines.xhtml

contacts to visit a health center to have their skin screened and, if eligible, to be offered SDR-PEP. Clustering of the disease within households is commonly seen.[6,48,49] Household contacts are defined as living under the same roof as the leprosy index patient for a minimum of 3 months (Table 1).[13,50,51] To prevent re-infection within a household and for operational management reasons, contacts need to visit the health center within 3 months after the index patient was included, which is also in-line with contact tracing interventions in literature.[52] Around 6 household contacts per patient are expected to visit the health center for screening.[13] Previous studies showed that leprosy patients are usually willing to disclose their leprosy diagnosis to their household members to facilitate screening and prophylaxis, but they are often reluctant to share this information with neighbors or other social contacts.[43–45]

Figure 3. Flow of participants through the PEP4LEP study

Integrated skin screening

For contact screening in both interventions, an integrated skin diseases approach - also called common skin screening approach - will be used to diagnose common skin diseases (e.g., eczema), skin conditions related to HIV/AIDS (e.g., Kaposi's sarcoma), and skin-NTDs (e.g., leprosy). "Integration" in this context refers to combined screening for a minimum of two diseases at the same time in the same communities.[53] In the PEP4LEP project, free topical treatment for the most frequently diagnosed skin diseases will be provided as well as referral advice, in-line with WHO and national medical guidelines.[54–58] The screening for signs and symptoms of skin diseases, as well as the chemoprophylaxis distribution, will follow standard operating procedures (SOPs) in which the eligibility criteria for SDR-PEP are clearly stated. In both interventions, the integrated skin diseases approach will be used and supported by the SkinApp, a mHealth tool developed by NLR and Erasmus University Medical Center (Erasmus MC).[30,31] The SkinApp will support peripheral health workers in recognizing and treating signs and symptoms of skin diseases, including skin-NTDs like leprosy.[30,31] A senior health staff member with sufficient dermatology experience (preferably a dermatologist) will attend in person or via secure medical messaging via the application (app) Siilo.[59]

Post-exposure prophylaxis

Chemoprophylaxis with SDR-PEP has been adopted in the 2018 WHO "Guidelines for the diagnosis, treatment and prevention of leprosy".[4] The SDR-PEP dosages used in this project (Table 2) are consistent with these WHO guidelines, the in 2020 published WHO document

For peer review only - http://bmjopen.bmj.com/site/about/guidelines.xhtml

"Leprosy/Hansen disease: Contact tracing and post-exposure prophylaxis. Technical guidance" and the LPEP program.[4,7,13]

Table 2. PEP4LEP single-dose rifampicin dosages[4,7,13]

Age and body weight of contact	Rifampicin dosage
≥15 years	600 mg
10-14 years	450 mg
6-9 years and body weight of ≥20 kg	300 mg
≥2 years old and body weight between 10-20 kg	150 mg

Contacts who are temporarily ineligible to receive SDR-PEP (e.g., because of pregnancy, Table 1) will receive skin screening and a SDR-PEP voucher, useable in an affiliated PEP4LEP health center when becoming eligible (e.g., after giving birth). Contacts receiving SDR-PEP will also receive a SDR-PEP Red Card to keep in their homes. This card indicates that the person has received SDR-PEP for leprosy prevention and is ineligible to receive this again within the next two years. These methods were previously used as part of the LPEP program in Tanzania.[13] In PEP4LEP, serious adverse events (SAEs) will be reported and followed up according to national and PEP4LEP guidelines (see ethical section).[60]

<u>Outcomes</u>

The primary objectives of this study are to identify the most effective and feasible approach for screening contacts of leprosy patients in combination with administering chemoprophylaxis to prevent leprosy (Table 3). Because of the long incubation period of leprosy, it will not be possible to observe reduced transmission at the population level, in terms of a reduced new case detection rate, during this project period. The active case finding component and raised awareness, however, are expected to lead to more detected cases, improved early case detection and reduced child cases and disability rates at the time of diagnosis. We hypothesize that enhanced case finding and integrated skin screening will lead to an overall reduction of detection delay in the community-based intervention over the study duration, driven by diagnosis of patients with early signs of leprosy (and shorter delays) that would otherwise go undetected.

Primary outcome measures

The primary outcome measures of effectiveness in the comparison of the two interventions are:

For peer review only - http://bmjopen.bmj.com/site/about/guidelines.xhtml

1) Case detection delay, measured in months since the first signs or symptoms of leprosy until diagnosis and in the number of patients with G2D.
2) Number of new patients with leprosy, subdivided into child proportion, female proportion, and multibacillary (MB) / paucibacillary (PB) classification.
3) Number of contacts screened and receiving SDR-PEP.

Secondary outcome measures

Feasibility will be assessed by looking at outcome measures related to cost-effectiveness and acceptability (Table 3):

- A cost-effectiveness analysis will be undertaken in the third year of the project, encompassing the costs incurred by the health system and the beneficiaries (out-of-pocket expenditure). It will include collecting indicators such as unit costs, costs per case detected, costs per disability-adjusted life years (DALY) averted and costs per extra case found. The current practice "routine service provision" will be compared with the two study interventions.
- The acceptability of both interventions will be determined by comparing the number of index patients and contacts included, as well as by using qualitative research methods, such as semi-structured interviews guided by topic lists, focus group discussions (FGDs) with relevant stakeholders and potentially ethnographic observations during the interventions for further data validation. More in-depth (country-specific) protocol descriptions on the acceptability and cost-effectiveness side-studies will be developed together with a health economist and social scientist.

Additional objectives

The additional objectives are to assess the acceptability of integrated skin screening and the use of the SkinApp as a supporting mHealth tool in the field, as well as health workers' capacity regarding the integrated skin screening approach (Table 3). This will be measured by the number of contacts diagnosed with skin diseases and NTDs and by recording the use of the SkinApp during contact screening. The capacity of health workers to diagnose leprosy and other skin diseases will be determined by a series of 4 assessments in which the SkinApp can be used: before (baseline) and after PEP4LEP training, during the study, at the end of the study.

The 4 assessments were designed in collaboration with an educational specialist and each include 30 questions (20 multiple choice questions on leprosy and 10 skin disease cases of which 5 formulated as open questions). The primary PEP4LEP health worker training is conducted over several days and consists of interactive training modules focusing on: the PEP4LEP research project, integrated skin screening including the use of mHealth tools (NLR's SkinApp and Siilo),

For peer review only - http://bmjopen.bmj.com/site/about/guidelines.xhtml

clinical leprosy and the administration of SDR-PEP.[4,27,28,30,59,61,62] Refresher trainings will also be organized. The capacity of health workers to diagnose leprosy and other skin diseases will be determined by the series of assessments in which the SkinApp can be used. Additionally, qualitative methods including semi-structured interviews, FGDs, and potentially ethnographic field observations will be used to gain a more in-depth understanding of these objectives.

Case detection delay

Case detection delay is defined by Muthuvel et al. as the number of months between the onset of signs and symptoms of leprosy and the time of diagnosis, including both "patient delay" (period in months between noticing the first sign/symptom to the first health care provider consultation) and "health-system delay" (period in months between the first health care provider consultation and the patient receiving the leprosy diagnosis).[63] Several studies have investigated delay in leprosy diagnosis in countries like Bangladesh, Brazil, India, Colombia, and Paraguay.[63–70] However, recent literature on delay in diagnosis is limited and mainly focuses on other geographical regions. Therefore, for this project, delay will be determined with a structured questionnaire designed in the project countries, with input from several stakeholders, which will be shared open access (publication expected). The questionnaire includes two annexes: a set of clinical photos of signs of leprosy and a context-specific calendar indicating important local dates, such as festivities, agricultural seasons and religious celebrations.[71,72] A "Question-by-Question Guide" was designed to provide support in the administration of the questionnaire. The questionnaires were culturally validated in all three countries, based on the conceptual framework of Herdman et al. (publication expected).[73]

Table 3. PEP4LEP project outcomes and statistical methods

Objective	Outcome	Hypothesis	Outcome measure	Method of analysis
1.1 To compare the effectiveness of a skin camp prophylaxis intervention with a health center-based prophylaxis intervention in terms of the rate of leprosy patients detected	Primary: Case detection delay	Reduction in case detection delay is expected to be greater in the community-based intervention compared with the health center-based household contact approach	Number of months since first signs or symptoms of leprosy until diagnosis (including assessing both "patient delay" and "health-	Descriptive statistics; multivariate models; non-parametric tests

For peer review only - http://bmjopen.bmj.com/site/about/guidelines.xhtml

and delay in case detection			system delay"); G2D percentage among newly diagnosed leprosy patients	
	Primary: Number of contacts diagnosed with leprosy	The community-based intervention will identify more cases of leprosy from contact screening compared with the health center household contact-based approach	Number of contacts diagnosed with leprosy; child proportion; female proportion; MB/PB classification of newly diagnosed leprosy patients	Descriptive statistics; Pearson's chi square test; Fisher's exact test; multivariate logistic regression analysis
	Primary: Number of contacts who received chemoprophylaxis	The community-based intervention will allow more contacts to be screened and receive SDR-PEP compared with the health center-based household contact approach	Number of contacts screened; number of contacts who received SDR-PEP	Descriptive statistics
1.2 To compare the feasibility of the two chemoprophylaxis interventions (screening household contacts or screening contacts via skin camps) in terms of cost- effectiveness and acceptability	Secondary: Cost-effectiveness of each intervention	The community-based intervention will be more expensive but will have a greater impact compared with the health center-based household contact approach	Number of index patients included; number of contacts screened; number of cases prevented; number of disabilities avoided; operational costs;	Health economic evaluations

For peer review only - http://bmjopen.bmj.com/site/about/guidelines.xhtml

			out-of-pocket expenses	
	Secondary: Acceptability of each intervention	Both interventions will be accepted in participating countries	Number of index patients included; number of contacts screened; and qualitative methods	Descriptive statistics; qualitative content analysis of interviews; FGDs and potentially observations
2.1 To assess the acceptability of an integrated skin diseases approach and the use of the SkinApp	Additional: Number of contacts diagnosed with other skin diseases	The community-based intervention will identify more cases of other skin diseases from contact screening compared with the health center-based household contact approach	Number of contacts diagnosed with skin diseases including and with NTDs that manifest with skin lesions	Descriptive statistics; Pearson's chi square test; Fisher's exact test; multivariate logistic regression analysis
	Additional: Acceptability of an integrated skin screening approach and the use of the SkinApp	The integrated skin screening approach will encourage screening participation, and the SkinApp will help health workers to diagnose skin diseases	Number of contacts diagnosed with skin diseases and with NTDs that manifest with skin lesions; utilization of the SkinApp during contact screening; and qualitative methods	Descriptive statistics; sensitivity and specificity; positive and negative predictive values; qualitative content analysis of interviews, FGDs, and potentially observations
2.2 To compare the capacity of health workers in diagnosing leprosy,	Additional: Capacity of health workers in diagnosing	Participation in training and the use of the SkinApp	Results of health worker capacity assessments and	Descriptive statistics; qualitative content analysis

For peer review only - http://bmjopen.bmj.com/site/about/guidelines.xhtml

other skin diseases and other NTDs that manifest with skin lesions before the start of the study with their capacity in the third year	leprosy and other skin diseases	will improve health worker capacity	qualitative methods	of interviews, FGDs, and potentially observations
Abbreviations: FGD: focus group discussion; G2D: grade-2 disability; MB: multibacillary; NTD: neglected tropical disease; PB: paucibacillary; SDR-PEP: single-dose rifampicin				

<u>Sample size</u>

The sample size calculation was based on case detection delay as the main outcome measure for comparing the effectiveness of each intervention. This measure was used for the calculation because the epidemiological impact (i.e. reduction in overall new case detection rate in PEP4LEP districts) will not become apparent within the study duration due to the long incubation time of leprosy. The mean or median delay will be compared between both interventions and with the baseline. A baseline case detection delay will be estimated in each country by interviewing recently diagnosed leprosy patients with the same structured questionnaire prior to the start of the study. For the sample size calculation, a literature-based estimated average case detection delay of 24 months for leprosy patients with a standard deviation of 8 months was used, with the conservative assumption that a minimal delay difference of 3 months would be detected between both interventions.[74,75] In order to achieve this, we aim to include at least 675 index patients in the study: 270 in the community-based intervention areas (30 per country per year) and 405 new patients in the health center-based intervention areas (45 per country per year). Approximately 100 contacts will be screened per index patient in the community-based intervention areas, and approximately 6 contacts will be screened per index patient in the health center-based intervention areas; thus, a total of approximately 30,000 contacts will be screened (Figure 3). We expect no major differences in case detection delay between clusters and within clusters, thus no significant design effect is foreseen. For the feasibility study component and additional research objectives, interviews and FGDs are planned. For the interviews, a minimum of 10 index patients, 10 household contacts, 10-20 community contacts, 10 health workers / community volunteers, 4 health decision makers and 10 community leaders will be included. For the FGDs, 6-10 participants will be included: 2 groups of index patients, 2 groups of household contacts, 2 groups of community contacts, 2 groups with health workers and 1-2 groups with decision makers. Contacts refusing to take SDR-PEP but who are willing to participate in the qualitative study component and community members outside of the 20 closest houses to the index patient in intervention 1 will also be included. The qualitative research sampling will be purposive,

For peer review only

For peer review only - http://bmjopen.bmj.com/site/about/guidelines.xhtml

according to the defined target groups, and balanced according to e.g. gender, age, education level, religion and/or socio-cultural background. All fully trained health staff involved in the PEP4LEP project will be asked to consent to enroll in the capacity assessment.

Randomization

PEP4LEP used randomization without blinding at the (clustered) health center level (health centers/posts), ensuring that clusters were similar in size. There are 17 health facilities included in Ethiopia, 22 in Mozambique and 23 in Tanzania. Blinding is not possible because of the varying operational components of the interventions. Cluster randomization is commonly used when trying to capture the impact of an intervention at community level on both infectiousness and susceptibility.[76] This method is stated to be feasible logistically, and contamination (e.g., information-sharing between participants from both interventions) is unlikely.[76] Randomization was performed using the statistical software package R.[77] Per country, health centers were randomly divided into the community-based intervention or health center-based intervention.

Data collection and management

The PEP4LEP data management plan was developed by Erasmus MC in collaboration with the consortium. Regarding quantitative data, collectors will record their findings onto paper-based forms. Information from the forms will be entered into the Research Electronic Data Capture (REDCap) system from Vanderbilt University.[78] The REDCap software will be linked to a centralized database server hosted by Erasmus MC.

To determine the cost-effectiveness, data for establishing costs (such as infrastructure, human resources, transportation) and output (such as number of contacts seen, rifampicin capsules provided, patients diagnosed with other NTD related skin diseases and treatments provided) will be derived from the ongoing surveillance data. Other costs (such as general programme costs, treatment costs and other direct or indirect costs) will be collected from ancillary studies.

Besides quantitative data, qualitative data will also be collected for the acceptability and health workers' capacity assessment. Data from (semi-)structured interviews, FGDs, and possible ethnographic observations will be audio-recorded and/or paper-based. Data will be transcribed (verbatim), translated to English and entered into computer-assisted qualitative data analysis software.[79] The transcriptions will be securely stored at Erasmus MC after analysis. A system of identification (ID) codes has been developed to record and maintain data systematically, as well as to maintain "pseudo-anonymity."

Data analysis

For peer review only - http://bmjopen.bmj.com/site/about/guidelines.xhtml

Data from the PEP4LEP study will be analyzed primarily through quantitative methods using descriptive analysis for all variables (Table 3). Mean and median case detection delays will be compared between both interventions and the established baseline. This includes newly diagnosed cases identified through each contact screening intervention as well as those detected through ongoing passive case finding, currently the primary method of detection in routine leprosy programs in the three countries. The *p*-values for each statistical test will be two-tailed with $p \leq 0.05$ considered significant and 95% confidence intervals (CI) presented for regression analyses. Quantitative analysis will be conducted using statistical software such as SPSS.[80]

The acceptability and capacity assessments will include qualitative research data (Table 3), which will be coded and analyzed using computer-assisted qualitative data analysis software, including Atlas.ti.[79,81] Data coding is necessary to categorize and define what the data signify by identifying concepts, patterns, relations, and themes.[82] Data reanalysis will occur until no new topics are emerging and data saturation is reached, which means that no significant new themes are emerging.[83]

Availability of data and materials

Data will be stored for 25 years according to EU regulation 536/2014 considering clinical medication-related research projects.[67] Data will be made available in a repository for potential authorized re-use for future data analysis or study replication. Sharing data and study materials as well as open access publishing are important values of the EU research and innovation program Horizon 2020, the European and Developing Countries Clinical Trials Partnership (EDCTP) and the PEP4LEP consortium.[67,84]

Patient and public involvement

Community leaders, people affected by leprosy, and representatives of disabled people organizations (DPO) are and will be involved in monitoring the study as well as in mobilizing community participation. Results will be reported back to the communities via community workshops. Capacity building is an important part of this project. Besides training health staff and community volunteers, four PhD-candidates will obtain a PhD from this project, of which three candidates originate from the endemic countries included in this project to increase local research capacity.[61]

Ethics

For peer review only - http://bmjopen.bmj.com/site/about/guidelines.xhtml

Ethical approval was obtained in each country according to national guidelines (Table 4). Erasmus MC, as European consortium member, received a waiver of full medical ethics review and approval from its ethical board according to the Dutch Medical Research Involving Human Subjects Act (Wet Medisch-Wetenschappelijk Onderzoek met mensen, WMO).[85]

Table 4. PEP4LEP ethical approvals

Country	Ethical board	Outcome	Primary approval/waiver date
**Ethiopia**	National Research Ethics Review Committee from the Ministry of Science and Higher Education (MoSHE)	Approved	17 February 2020
**Mozambique**	Comité Nacional de Bioética para a Saúde (CNBS) from the Ministério da Saúde	Approved	16 August 2019
**Tanzania**	Ethical Clearance Committee linked to the National Institute for Medical Research (NIMR) and Ministry of Health, Community Development, Gender, Elderly & Children (MoHCDEC)	Approved	17 June 2019
**The Netherlands**	Medical Ethics Committee Erasmus University Medical Center	Waiver	11 April 2019

Written (or thumbprint) informed consent will be obtained from all study participants. If a participant is below 18 years old, a parent or legal guardian will be asked for consent. Study information given to the study participants prior to asking for consent contains details about: leprosy; the study purpose; the right to withdraw; the fact that SDR-PEP leads to a leprosy risk reduction and not absolute prevention (i.e. awareness of leprosy signs/symptoms remains important after taking SDR-PEP); possible side effects of SDR-PEP (i.e., urine discoloration) and AEs; the incidental findings procedure; and national contact information. AEs are expected to be rare after SDR-PEP. In the LPEP study, in which SDR-PEP was administered to 151,928 screened contacts, a single adverse event was reported (an allergic reaction to rifampicin in Brazil) and no serious adverse events were seen.[13] Urine discoloration, a common rifampicin side-effect, was not considered as an AE requiring follow-up in LPEP. Nevertheless, in

For peer review only - http://bmjopen.bmj.com/site/about/guidelines.xhtml

(chemo)prophylaxis programs AEs are of utmost importance because large numbers of healthy individuals are involved. In PEP4LEP, SAEs will be reported following national pharmacovigilance guidelines and by using the PEP4LEP AE Form for registration and to inform the principal investigator.[13,60] The PEP4LEP project's SOP on rifampicin administration therefore included the availability of an emergency allergy kit at community study sites where no health center is located, which should be used according to national medical/pharmacological guidelines.[56–58] All participants with suspected AEs will be referred for proper medical management and treated free of charge according to national standard treatment guidelines.[60]

Throughout both screening interventions and research projects involving human subjects, incidental findings with potential health importance may be observed.[86] Incidental findings are discoveries made during a research or screening project which are outside the scope of the project.[87] Examples of possible incidental findings when performing full body skin screening include: signs of cancer, venous insufficiency, bleeding diathesis, herniation, dental problems, or indications of possible abuse. Incidental findings in a research setting are often not explicit enough to be used for diagnosis, treatment, or clinical care.[88]
The procedures for reporting both SAEs and incidental findings are included in the evidence-based PEP4LEP SOPs, on the participant information sheet and in the health workers' training [60,86,87,89,90] The importance will also be emphasized during ongoing monitoring activities, including field visits.[13]

During the developmental phase of this project, the COVID-19 pandemic emerged. Regarding COVID-19, national governmental and WHO guidelines will be followed.[33–36] Information about COVID-19 and project implications (e.g., physical distancing, working in time slots) are included in the project's SOPs, Information, Education and Communication (IEC) materials and health workers' training. Hand washing facilities and personal protective equipment (PPE) such as gloves, face masks and aprons, will be made available at the study sides.

A code of conduct will be designed for the PEP4LEP consortium, based on the code of conducts from WHO and All European Academies (ALLEA).[91,92] All researchers in the project are encouraged to participate in good clinical practice (GCP) courses, facilitated by the research consortium.[93] National data-safety monitoring boards, an international publication committee, and an international scientific steering committee were formed to monitor the project (Figure 1).

<u>Trial registration</u>

The PEP4LEP project is registered at The Netherlands Trial Register (NTR), receiving trial registration number NL7294 (NTR7503), registration date September 10, 2018.[94]

For peer review only - http://bmjopen.bmj.com/site/about/guidelines.xhtml

Dissemination

Study outcomes are expected to be relevant for other sub-Saharan countries, but also for leprosy endemic areas outside the African context. Results will be shared open-access via peer-reviewed journals and at conferences. Tools designed for this study will be made available via https://www.infolep.org, the international knowledge center for information resources on leprosy.[95] Best practices will be shared with the Global Partnership for Zero Leprosy (GPZL).[96] Communities affected and local and national policymakers will be informed on the study outcomes via community meetings/workshops. In addition, project recommendations will be offered to all relevant authorities and the WHO in Ethiopia, Mozambique and Tanzania; the uptake of SDR-PEP into national leprosy guidelines is advised by the WHO.[8]

Discussion

The PEP4LEP study will use an integrated skin screening approach, which is also recommended by the WHO.[1,19,20] Skin diseases are among the most common human illnesses, affecting almost 900 million people worldwide.[23] They are thought to be the fourth leading cause of global non-fatal disease burden and can result in disabilities, stigmatization, and discrimination.[23,97] Additionally, dermatological problems can be the first expression of systemic or chronic diseases, including HIV/AIDS, diabetes, and NTDs.[17,98] Integrated skin screening is therefore expected to generate a greater health benefit compared with vertical health programs which focus on one disease only. Pooling diseases in projects like PEP4LEP can also be helpful in educating and in raising awareness, as health workers' knowledge of NTDs like leprosy has been declining.[53,99,100] This was reflected in a study performed by Abeje et al. among general health workers diagnosing leprosy in Ethiopia, which revealed that only 18% diagnosed leprosy correctly.[101] Detecting skin NTDs like leprosy therefore requires capacity-strengthening programs.[17,19,25–29]

This study will also use mHealth solutions to support peripheral health workers in recognizing and treating signs and symptoms of skin diseases. "Digital health applications in leprosy" is described as key research topic in the WHO "Global Leprosy Strategy 2021–2030".[8] Evidence indicates that mobile technology tools can substantially benefit healthcare workers, their patients, and adequate health care delivery.[102] In dermatology, electronic health (eHealth) was adopted early, with teledermatology as a widespread example, fostering the possibility of remote patient care and education.[103,104] This is especially valuable if health services are scarce and during periods of service disruption (e.g., flooding, civil unrest, COVID-19 pandemic).[36,59,62,104,105] We emphasize

For peer review only - http://bmjopen.bmj.com/site/about/guidelines.xhtml

the importance of studying the effect of mHealth technologies, aimed at capacity strengthening, like NLR's SkinApp, before fully focusing on upscaling.[30,31,62,102]

Despite the conclusion of the expert meeting that SDR-PEP poses negligible risk of generating rifampicin resistance in *M. tuberculosis*, ongoing resistance surveillance is important to consider.[15,106–108] However, because of the limited study period, resistance surveillance in the PEP4LEP implementation areas alone would add no value to the project as the number of patients will be too small and the project duration would be too short for any resistance to emerge during that period. It is therefore recommended to integrate the surveillance of rifampicin resistance in the PEP4LEP project areas with the resistance surveillance systems for TB and leprosy during the project period and beyond, consistent with WHO recommendations on resistance surveillance.[106–108]

Although SDR-PEP has been adopted in the WHO guidelines on leprosy, little is known about the feasibility of several implementation methods of SDR as chemoprophylaxis for leprosy in combination with varying and integrated contact screening methods, especially in sub-Saharan Africa.[4] Tanzania was the only sub-Sahara African country included in the LPEP Program.[13] Ortuno-Gutierrez et al. recently outlined the Post-Exposure Prophylaxis for Leprosy in the Comoros and Madagascar (PEOPLE) study protocol.[109] PEOPLE assesses the effectiveness of different modalities of SDR-PEP, using door-to-door surveys and a double dose of SDR-PEP. Both the PEOPLE and the PEP4LEP research questions comply with the Aligned Research Agenda for Zero Leprosy from the GPZL regarding the call for more operational studies and research focusing on SDR-PEP and on digital health.[110,111] Too often, innovative medical interventions fail because the factors contributing to success are poorly understood and hence not considered.[112] Lessons learned from SDR-PEP implementation are also expected to be relevant when improved preventive approaches, such as new chemotherapeutic regimens and vaccines, become available in the future.[8,108] Therefore, our goal is to share key insights gained from the PEP4LEP study to foster the implementation of integrated skin screening and chemoprophylaxis for leprosy in the sub-Sahara African context, which may also be relevant for the global leprosy community.

Declarations

Acknowledgments

Our thanks go to all those involved in the PEP4LEP project, including the study participants; the full research consortium; and our funders European and Developing Countries Clinical Trials Partnership (EDCTP) and Leprosy Research Initiative (LRI).

For peer review only - http://bmjopen.bmj.com/site/about/guidelines.xhtml

Author contributions

LM, CK, JHR, AS, TH and RvW designed the study. KB, FM, SEM, EM, AM, NM, TL, AMM, DVK, AME, LR, BN supported the development of country-specific protocols, materials and coordinate the study implementation. AS, TH and RvW have drafted the manuscript. All authors have reviewed the draft manuscript and have read and approved the final version.

Funding

This project was supported by the EDCTP2 program under Horizon 2020 (grant number RIA2017NIM-1839-PEP4LEP). The project also received funding from the Leprosy Research Initiative (LRI; www.leprosyresearch.org) under LRI grant number 707.19.58. Both funding bodies reviewed and approved the study proposal.

Competing interests

No competing interest have been declared by the authors.

For peer review only

References

1. World Health Organization. Promoting an integrated approach to enhance detection of neglected tropical diseases of the skin. World Health Organisation. https://www.who.int/neglected_diseases/news/Promoting-453 integrated-approach-to-enhance-detection-skin-NTDs/en/. Published 2019. Accessed June 19, 2019.
2. World Health Organization. Weekly epidemiological record / Relevé épidémiologique hebdomadaire. *Wkly Epidemiol Rec*. 2020;95(36):417-440.
3. Smith CS, Noordeen SK, Richardus JH, et al. A strategy to halt leprosy transmission. *Lancet Infect Dis*. 2014;14(2):96-98. doi:10.1016/S1473-3099(13)70365-7
4. World Health Organization. *Guidelines for the Diagnosis, Treatment and Prevention of Leprosy*. New Delhi: World Health Organization; 2018.
5. Van Beers SM, Hatta M, Klatser PR. Patient contact is the major determinant in incident leprosy: Implications for future control. *Int J Lepr Other Mycobact Dis*. 1999;67(2):119-128.
6. Moet FJ, Pahan D, Schuring RP, Oskam L, Richardus JH. Physical distance, genetic relationship, age, and leprosy classification are independent risk factors for leprosy in contacts of patients with leprosy. *J Infect Dis*. 2006;193(3):346-353. doi:10.1086/499278
7. World Health Organization. *Leprosy/Hansen Disease: Contact Tracing and Post-Exposure Prophylaxis - Technical Guide*. (Cooreman E, ed.).; 2020.

https://www.who.int/publications/i/item/9789290228073. Accessed May 18, 2021.

8. World Health Organization. *Towards Zero Leprosy. Global Leprosy (Hansen's Disease) Strategy 2021–2030.*; 2021. https://www.who.int/publications/i/item/9789290228509. Accessed June 2, 2021.

9. Smith WC, van Brakel W, Gillis T, Saunderson P, Richardus JH. The Missing Millions: A Threat to the Elimination of Leprosy. Lockwood DNJ, ed. *PLoS Negl Trop Dis*. 2015;9(4):e0003658. doi:10.1371/journal.pntd.0003658

10. Taal AT, Blok DJ, van Brakel WH, de Vlas SJ, Richardus JH. Number of people requiring post-exposure prophylaxis to end leprosy: A modeling study. Pappas G, ed. *PLoS Negl Trop Dis*. 2021;15(2):e0009146. doi:10.1371/journal.pntd.0009146

11. Moet FJ, Pahan D, Oskam L, Richardus JH. Effectiveness of single dose rifampicin in preventing leprosy in close contacts of patients with newly diagnosed leprosy: cluster randomised controlled trial. *Bmj*. 2008;336(7647):761-764.

12. Idema WJ, Majer IM, Pahan D, Oskam L, Polinder S, Richardus JH. Cost-effectiveness of a chemoprophylactic intervention with single dose rifampicin in contacts of new leprosy patients. *PLoS Negl Trop Dis*. 2010;4(11):e874-e874. doi:10.1371/journal.pntd.0000874

13. Richardus JH, Tiwari A, Barth-Jaeggi T, et al. Leprosy post-exposure prophylaxis with single-dose rifampicin (LPEP): an international feasibility programme. *Lancet Glob Heal*. 2021;9(1):e81-e90. doi:10.1016/S2214-109X(20)30396-X

14. Tiwari A, Blok DJ, Arif M, Richardus JH. Leprosy post-exposure prophylaxis in the indian health system: A cost-effectiveness analysis. *PLoS Negl Trop Dis*. 2020;14(8):1-12. doi:10.1371/journal.pntd.0008521

15. Mieras L, Anthony R, van Brakel W, et al. Negligible risk of inducing resistance in Mycobacterium tuberculosis with single-dose rifampicin as post-exposure prophylaxis for leprosy. *Infect Dis Poverty*. 2016;5(1):46. doi:10.1186/s40249-016-0140-y

16. Blok DJ, Steinmann P, Tiwari A, et al. The long-term impact of the leprosy post-exposure prophylaxis (Lpep) program on leprosy incidence: a modelling study. *PLoS Negl Trop Dis*. 2021;15(3):e0009279. doi:10.1371/journal.pntd.0009279

17. Yotsu RR. Integrated Management of Skin NTDs-Lessons Learned from Existing Practice and Field Research. *Trop Med Infect Dis*. 2018;3(4):120. doi:10.3390/tropicalmed3040120

18. World Health Organization. Recognizing neglected tropical diseases through changes on the skin. WHO. http://www.who.int/neglected_diseases/resources/9789241513531/en/. Published 2018. Accessed July 27, 2020.

19. Mitjà O, Marks M, Bertran L, et al. Integrated control and management of neglected tropical skin diseases. *PLoS Negl Trop Dis*. 2017;11(1):e0005136.

20. Hay RJ, Asiedu K. Skin-Related Neglected Tropical Diseases (Skin NTDs)—A New Challenge. *Trop Med Infect Dis*. 2018;4(1):4. doi:10.3390/tropicalmed4010004

For peer review only

For peer review only - http://bmjopen.bmj.com/site/about/guidelines.xhtml

21. Kabatereine NB, Malecela M, Lado M, Zaramba S, Amiel O, Kolaczinski JH. How to (or not to) integrate vertical programmes for the control of major neglected tropical diseases in Sub-Saharan Africa. Brooker S, ed. 2010;4(6). doi:10.1371/journal.pntd.0000755
22. Ryan TJ, Ersser SJ, Fuller LC. The Public Health Intervention of Skin Care for All: Community Dermatology. In: *Public Health - Social and Behavioral Health*. InTech; 2012. doi:10.5772/36326
23. Hay RJ, Johns NE, Williams HC, et al. The Global Burden of Skin Disease in 2010: An Analysis of the Prevalence and Impact of Skin Conditions. *J Invest Dermatol*. 2014;134(6):1527-1534. doi:10.1038/jid.2013.446
24. Mosam A, Todd G. Dermatology Training in Africa: Successes and Challenges. *Dermatol Clin*. 2021;39(1):57-71. doi:10.1016/j.det.2020.08.006
25. Abdela SG, Diro E, Zewdu FT, et al. Looking for NTDs in the skin; an entry door for offering patient centered holistic care. *J Infect Dev Ctries*. 2020;14(06.1):16S-21S. doi:10.3855/jidc.11707
26. Figueroa JI, Fuller LC, Abraha A, Hay RJ. Dermatology in southwestern Ethiopia: rationale for a community approach. *Int J Dermatol*. 1998;37(10):752-758. doi:10.1046/j.1365-4362.1998.00425.x
27. Hay RJ, Estrada R, Grossmann H. Managing skin disease in resource-poor environments - the role of community-oriented training and control programs. *Int J Dermatol*. 2011;50(5):558-563. doi:10.1111/j.1365-4632.2011.04954.x
28. Faye O, Hay RJ, Ryan TJ, Keita S, Traore AK, Mahe A. A public health approach for leprosy detection based on a very short term-training of primary health care workers in basic dermatology. *Lepr Rev*. 2007;78(1):11.
29. Mahe A, N'Diaye HT, Bobin P. The proportion of medical consultations motivated by skin diseases in the health centers of Bamako (Republic of Mali). *Int J Dermatol*. 1997;36(3):185-186. doi:10.1046/j.1365-4362.1997.00140.x
30. NLR. SkinApp. https://nlrinternational.org/what-we-do/projects/skinapp/. Accessed July 30, 2020.
31. Mieras L, Taal A, Post E, Ndeve A, van Hees C. The development of a mobile application to support peripheral health workers to diagnose and treat people with skin diseases in resource-poor settings. *Trop Med Infect Dis*. 2018;3(3):102.
32. Raphael KG, Cloitre M, Dohrenwend BP. Problems of recall and misclassification with checklist methods of measuring stressful life events. *Health Psychol*. 1991;10(1):62-74. doi:10.1037/0278-6133.10.1.62
33. World Health Organization. Coronavirus disease (COVID-19). https://www.who.int/emergencies/diseases/novel-coronavirus-2019. Accessed July 17, 2020.

For peer review only - http://bmjopen.bmj.com/site/about/guidelines.xhtml

34. World Health Organization. Considerations for implementing mass treatment, active case-finding and population-based surveys for neglected tropical diseases in the context of the COVID-19 pandemic Interim guidance. WHO. https://www.who.int/publications/i/item/WHO-2019-nCoV-neglected-tropical-diseases-2020-1. Published 2020. Accessed August 3, 2020.

35. Government of the Netherlands. Frequently Asked Questions about Coronavirus (COVID-19) and Telehealth. 2020. https://www.government.nl/topics/coronavirus-covid-19/frequently-asked-questions-about-coronavirus-and-health. Accessed July 17, 2020.

36. World Health Organization. COVID-19 significantly impacts health services for noncommunicable diseases. *https://www.who.int/news/item/01-06-2020-Covid-19-Significantly-Impacts-Health-Services-for-Noncommunicable-Diseases*. 2020;41(June):1-3. https://www.who.int/news-room/detail/01-06-2020-covid-19-significantly-impacts-health-services-for-noncommunicable-diseases. Accessed July 24, 2020.

37. Mayo Clinic Staff. Tuberculosis. Diseases & Conditions. https://www.mayoclinic.org/diseases-conditions/tuberculosis/symptoms-causes/syc-20351250. Published 2018. Accessed July 30, 2018.

38. Gajuryal SH, Gautam S, Satyal N, Pant B. Organizing a Health Camp: Management Perspective. *Nepal Med J*. 2019;2(1):196-198. doi:10.3126/nmj.v2i1.23557

39. Ali M. What Are the Requirements to Organize a Free Medical Camp. https://www.transparenthands.org/what-are-the-requirements-to-organize-a-free-medical-camp/. Published 2018. Accessed July 17, 2019.

40. Grover S, Ranyal RK, Bedi MK. A cross section of skin diseases in rural Allahabad. *Indian J Dermatol*. 2008;53(4):179-181. doi:10.4103/0019-5154.44789

41. Tindana POO, Singh JAA, Tracy CSS, et al. *Grand Challenges in Global Health: Community Engagement in Research in Developing Countries*. Vol 4. Public Library of Science; 2007:1451-1455. doi:10.1371/journal.pmed.0040273

42. Shrestha R, Shrestha DP, Lama L, Gurung D, Rosdahl I. Pattern of skin diseases in a rural village development community of Nepal. *Nepal J Dermatology, Venereol Leprol*. 2014;12(1):41-44.

43. Feenstra SG, Nahar Q, Pahan D, Oskam L, Richardus JH. Acceptability of chemoprophylaxis for household contacts of leprosy patients in Bangladesh: a qualitative study. *Lepr Rev*. 2011;82(2):178-187.

44. Espiridion-Calma ADVD V, Dofitas BLL, Elinor M, Sison GQ, Sison MEGQ. Acceptability of immunoprophylaxis and/or chemoprophylaxis for household contacts of patients with Hansen's disease: A prospective, single-center, mixed methods study. *Acta Med Philipp*. 2020;54(3). doi:10.47895/amp.v54i3.1663

45. Peters R, Mieras L, Subedi M, et al. A single dose of rifampicin to prevent leprosy:

For peer review only

For peer review only - http://bmjopen.bmj.com/site/about/guidelines.xhtml

Qualitative analysis of perceptions of persons affected, contacts, community members and health professionals towards chemoprophylaxis and the impact on their attitudes in India, Nepal and Indonesia. *Lepr Rev*. 2018;89(4):335-352. doi:10.47276/lr.89.4.335

46. Aarogyasri Health Care Trust. *Revised Health Camp Policy-Guidelines*. https://www.aarogyasri.telangana.gov.in/documents/10202/0/Revised+Health+Camp+Policy.pdf/e5571 794475-1546-4221-9f63-f9a3ca1d2005. Accessed June 17, 2019.

47. Sathya Sai International Medical Committee. Guidelines for Medical Camps Conducted under the The Auspices of Sathya Sai International Organization. https://www.sathyasai.org/organisation/guidelines/medical-camps. Published 2013. Accessed April 8, 2019.

48. Hoeven TA, Fischer EAJ, Pahan D, Richardus JH. Social distance and spatial distance are not the same, observations on the use of GIS in leprosy epidemiology. *Epidemiol Infect*. 2008;136(12):1624-1627. doi:10.1017/S0950268808000381

49. Fischer E, De Vlas S, Meima A, Habbema D, Richardus J. Different mechanisms for heterogeneity in leprosy susceptibility can explain disease clustering within households. *PLoS One*. 2010;5(11):e14061-e14061. doi:10.1371/journal.pone.0014061

50. Cavaliero A, Greter H, Fürst T, et al. An innovative approach to screening and chemoprophylaxis among contacts of leprosy patients in low endemic settings: experiences from Cambodia. Small PLC, ed. *PLoS Negl Trop Dis*. 2019;13(3):e0007039. doi:10.1371/journal.pntd.0007039

51. Khoudri I, Elyoussfi Z, Mourchid Y, et al. Trend analysis of leprosy in Morocco between 2000 and 2017: Evidence on the single dose rifampicin chemoprophylaxis. *PLoS Negl Trop Dis*. 2018;12(12):e0006910. doi:10.1371/journal.pntd.0006910

52. Smith WCS, Aerts A. Role of contact tracing and prevention strategies in the interruption of leprosy transmission. *Lepr Rev*. 2014;85(1):2-17.

53. Chandler DJ, Fuller LC. The Skin-A Common Pathway for Integrating Diagnosis and Management of NTDs. *Trop Med Infect Dis*. 2018;3(3):101. doi:10.3390/tropicalmed3030101

54. World Health Organization. *WHO Model List of Essential Medicines - 21st List, 2019.*; 2019. https://www.who.int/publications/i/item/WHOMVPEMPIAU201907. Accessed June 18, 2021.

55. World Health Organization. *WHO Model List of Essential Medicines for Children - 7th List, 2019.*; 2019. https://www.who.int/publications/i/item/WHOMVPEMPIAU201907. Accessed June 18, 2021.

56. Food Medicine and Healthcare Administration and Control Authority of Ethiopia. *Standard Treatment Guidelines for Primary Hospital - Third Edition, 2014.*; 2014. https://www.pascar.org/uploads/files/Ethiopia_-_Primary_Hospital_CPG.PDF. Accessed

For peer review only

August 2, 2018.

57. República de Moçambique Ministério da Saúde. *Lista Nacional de Medicamentos Essenciais.*; 2017. https://www.afro.who.int/sites/default/files/2018-07/LISTA NACIONAL DE MEDICAMENTOS ESSENCIAIS 2017.pdf. Accessed July 24, 2018.
58. The United Republic of Tanzania Ministry of Health and Social Welfare. *Standard Treatment Guidelines & National Essential Medicines List Tanzania Mainland - Fourth Edition.*; 2013. https://www.who.int/selection_medicines/country_lists/Tanzania_STG_052013.pdf. Accessed August 2, 2018.
59. Siilo B.V. The free secure messaging app for medical team players - Siilo. https://www.siilo.com/. Published 2016. Accessed November 27, 2018.
60. World Health Organization. *Assuring Safety of Preventive Chemotherapy Interventions for the Control of Neglected Tropical Diseases.*; 2011. http://www.who.int/about/licensing/. Accessed July 27, 2020.
61. Cancedda C, Farmer PE, Kerry V, et al. Maximizing the Impact of Training Initiatives for Health Professionals in Low-Income Countries: Frameworks, Challenges, and Best Practices. *PLoS Med.* 2015;12(6). doi:10.1371/journal.pmed.1001840
62. Aranda-Jan CB, Mohutsiwa-Dibe N, Loukanova S. Systematic review on what works, what does not work and why of implementation of mobile health (mHealth) projects in Africa. *BMC Public Health.* 2014;14(1):188. doi:10.1186/1471-2458-14-188
63. Muthuvel T, Govindarajulu S, Isaakidis P, et al. "I Wasted 3 Years, Thinking It's Not a Problem": Patient and Health System Delays in Diagnosis of Leprosy in India: A Mixed-Methods Study. *PLoS Negl Trop Dis.* 2017;11(1):1-15. doi:10.1371/journal.pntd.0005192
64. Fischer EAJ, De Vlas SJ, Habbema JDF, Richardus JH. The long term effect of current and new interventions on the new case detection of leprosy: a modeling study. *PLoS Negl Trop Dis.* 2011;5(9):e1330.
65. Henry M, GalAn N, Teasdale K, et al. Factors Contributing to the Delay in Diagnosis and Continued Transmission of Leprosy in Brazil – An Explorative, Quantitative, Questionnaire Based Study. *PLoS Negl Trop Dis.* 2016;10(3):1-12. doi:10.1371/journal.pntd.0004542
66. Deps PD, Guedes BVS, Filho B, Andreatta MK, Marcari RS, Rodrigues LC. Delay in the diagnosis of leprosy in the Metropolitan Region of Vito. *Lepr Rev.* 2006;77(1):41-47.
67. European Commission. Guidelines on Open Access to Scientific Publications and Research Data in Horizon 2020, Version 2.1. https://ec.europa.eu/research/participants/data/ref/h2020/grants_manual/hi/oa_pilot/h2020-hi-oa-pilot-guide_en.pdf. Published 2016. Accessed July 31, 2018.
68. Srinivas G, Muthuvel T, Lal V, Vaikundanathan K, Schwienhorst-Stich EM, Kasang C. Risk of disability among adult leprosy cases and determinants of delay in diagnosis in five

For peer review only - http://bmjopen.bmj.com/site/about/guidelines.xhtml

states of India: A case-control study. *PLoS Negl Trop Dis*. 2019;13(6). doi:10.1371/journal.pntd.0007495

69. Gómez L, Rivera A, Vidal Y, et al. Factors associated with the delay of diagnosis of leprosy in north-eastern Colombia: a quantitative analysis. *Trop Med Int Heal*. 2018;23(2):193-198. doi:10.1111/tmi.13023

70. Nicholls PG, Wiens C, Smith WCS. Delay in Presentation in the Context of Local Knowledge and Attitude Towards Leprosy—The Results of Qualitative Fieldwork in Paraguay. *Int J Lepr Other Mycobact Dis*. 2003;71(3):198. doi:10.1489/1544-581X(2003)71<198:DIPITC>2.0.CO;2

71. Schmier JK, Halpern MT. Patient recall and recall bias of health state and health status. *Expert Rev Pharmacoecon Outcomes Res*. 2004;4(2):159-163. doi:10.1586/14737167.4.2.159

72. Stull DE, Leidy NK, Parasuraman B, Chassany O. Optimal recall periods for patient-reported outcomes: challenges and potential solutions. *Curr Med Res Opin*. 2009;25(4):929-942. doi:10.1185/03007990902774765

73. Herdman M, Fox-Rushby J, Badia X. A model of equivalence in the cultural adaptation of HRQoL instruments: the universalist approach. *Qual Life Res*. 1998;7(4):323-335.

74. Li J, Yang L, Wang Y, Liu H, Liu J, Cross H. How to improve early case detection in low endemic areas with pockets of leprosy: a study of newly detected leprosy patients in Guizhou Province, People's Republic of China. *Lepr Rev*. 2016;87(1):23-31. doi:10.47276/lr.87.1.23

75. Van Veen NHJ, Meima A, Richardus JH. The relationship between detection delay and impairment in leprosy control: a comparison of patient cohorts from Bangladesh and Ethiopia. *Lepr Rev*. 2006;77(4):356.

76. Hayes RJ, Alexander NDE, Bennett S, Cousens SN. Design and analysis issues in cluster-randomized trials of interventions against infectious diseases. *Stat Methods Med Res*. 2000;9(2):95-116. doi:10.1191/096228000670953670

77. Foundation TR. The R Project for Statistical Computing The R Project for Statistical Computing. https://www.r-project.org/. Published 2015. Accessed November 9, 2018.

78. Vanderbilt University. About – REDCap. https://projectredcap.org/about/. Accessed February 24, 2020.

79. Denzin NK, Lincoln YS. *Collecting and Interpreting Qualitative Materials*. Sage Publications; 2008.

80. IBM Analytics. IBM SPSS Software. International Business Machines Corporation. https://www.ibm.com/analytics/spss-statistics-software. Published 2016. Accessed May 1, 2020.

81. Scientific Software Development GmbH. ATLAS.ti: The Qualitative Data Analysis;

For peer review only - http://bmjopen.bmj.com/site/about/guidelines.xhtml

For peer review only

Research Software. Web. https://atlasti.com/. Published 2017. Accessed March 23, 2020.

82. Gibbs G. Thematic Coding and Categorizing. In: *Analyzing Qualitative Data*. ; 2012:38-55. doi:10.4135/9781849208574.n4

83. Morse JM. The Significance of Saturation. *Qual Health Res*. 1995;5(2):147-149. doi:10.1177/104973239500500201

84. European and Developing Countries Clinical Trials Partnership. EDCTP2 policy on clinical trials registration, publication and data. http://www.edctp.org/publication/edctp2-policy-on-clinical-trials-registration-publication-and-data-sharing/. Published 2018. Accessed August 31, 2019.

85. Centrale Commissie Mensgebonden Onderzoek. Uw onderzoek: WMO-plichtig of niet? https://www.ccmo.nl/onderzoekers/wet-en-regelgeving-voor-medisch-wetenschappelijk-onderzoek/uw-onderzoek-wmo-plichtig-of-niet. Accessed July 31, 2018.

86. Wolf SM, Lawrenz FP, Nelson CA, et al. Managing Incidental Findings in Human Subjects Research: Analysis and Recommendations. *J Law, Med Ethics*. 2008;36(2):219-248. doi:10.1111/j.1748-720X.2008.00266.x

87. University of Waterloo - Office of Research Ethics. Guideline for the Reporting of Incidental and Secondary Findings to Study Participants University of Waterloo Office of Research Ethics. https://uwaterloo.ca/research/sites/ca.research/files/uploads/files/guideline_on_incidental_findings_reporting_october_2014.pdf. Published 2014. Accessed July 20, 2020.

88. Phung C. Ethics of disclosing results of genetic testing of donor-derived leukemia to recipient in a hereditary cancer biology research setting Connie Phung, MS 1The. *bioethics.yale.edu*. https://bioethics.yale.edu/sites/default/files/files/Ethics of Donor Derived Leukemia.pd. Accessed December 13, 2018.

89. Illes J, Kirschen MP, Edwards E, et al. Ethics. Incidental findings in brain imaging research. *Science*. 2006;311(5762):783-784. doi:10.1126/science.1124665

90. Europe C of. Convention for the protection of human rights and dignity of the human being with regard to the application of biology and medicine: Convention on human rights and biomedicine. *Eur J Health Law*. 1997;4(1):89-100. doi:10.1163/15718099720521896

91. World Health Organization. WHO Ethics: Promoting compliance, risk management and ethics. www.who.int/about/ethics. Published 2003. Accessed January 8, 2020.

92. All European Academies. The European Code of Conduct for Research Integrity. https://ec.europa.eu/info/funding-tenders/opportunities/docs/2021-2027/horizon/guidance/european-code-of-conduct-for-research-integrity_horizon_en.pdf. Published 2017. Accessed October 20, 2020.

93. European Union. Good clinical practice. https://ec.europa.eu/health/sites/health/files/files/eudralex/vol-10/3cc1aen_en.pdf.

For peer review only

For peer review only - http://bmjopen.bmj.com/site/about/guidelines.xhtml

Published 1997. Accessed August 6, 2018.

94. Netherlands Trial Register. Trial NL7294 (NTR7503) - PEP4LEP. https://www.trialregister.nl/trial/7294. Published 2018. Accessed June 21, 2021.

95. Infolep. Infolep - International knowledge center for information resources on leprosy. https://www.leprosy-information.org/. Accessed May 28, 2021.

96. Global Partnership for Zero Leprosy. Zero Leprosy Toolkit - Best Practices to reach no disease, no disability, & no stigma. https://zeroleprosy.org/toolkit/. Accessed May 28, 2021.

97. Mphande FA. Skin Diseases: Need for Attention. *Ski Disord Vulnerable Popul*. 2020:1-12. doi:10.1007/978-981-15-3879-7_1

98. Engelman D, Fuller LC, Solomon AW, et al. Opportunities for Integrated Control of Neglected Tropical Diseases That Affect the Skin. *Trends Parasitol*. 2016;32(11):843-854. doi:10.1016/j.pt.2016.08.005

99. Henderson CA. Skin disease in rural Tanzania. *Int J Dermatol*. 1996;35(9):640-642.

100. Muloliwa AM, Dreva D, Banquimane M, et al. Descrição da tendência de registo de casos de lepra em três distritos de Nampula, 2014-2018. *II Jornadas Reg Saúde - Região Norte, Programa Científico e Livro Resumo*. 2019:61.

101. Abeje T, Negera E, Kebede E, et al. Performance of general health workers in leprosy control activities at public health facilities in Amhara and Oromia States, Ethiopia. *BMC Health Serv Res*. 2016;16(1):122. doi:10.1186/s12913-016-1329-2

102. White A, Thomas DSKSK, Ezeanochie N, Bull S. *Health Worker MHealth Utilization: A Systematic Review*. Vol 34. Lippincott Williams and Wilkins; 2016:206-214. doi:10.1097/CIN.0000000000000231

103. Faye O, Bagayoko CO, Dicko A, et al. A teledermatology pilot programme for the management of skin diseases in primary health care centres: Experiences from a resource-limited country (Mali, West Africa). *Trop Med Infect Dis*. 2018;3(3):88. doi:10.3390/tropicalmed3030088

104. Wurm EMT, Hofmann-Wellenhof R, Wurm R, Soyer HPP. Telemedicine and teledermatology: Past, present and future. *JDDG*. 2008;6(2):106-112. doi:10.1111/j.1610-0387.2007.06440.x

105. Källander K, Tibenderana JK, Akpogheneta OJ, et al. Mobile health (mhealth) approaches and lessons for increased performance and retention of community health workers in lowand middle-income countries: A review. *J Med Internet Res*. 2013;15(1):e17. doi:10.2196/jmir.2130

106. World Health Organization. Guidelines for surveillance of drug resistance in tuberculosis - Fourth Edition. https://www.who.int/tb/publications/surveillance_guidelines/en/. Published 2009. Accessed April 6, 2020.

107. World Health Organization. A guide for surveillance of antimicrobial resistance in leprosy.

For peer review only - http://bmjopen.bmj.com/site/about/guidelines.xhtml

https://www.who.int/lep/resources/9789290226192/en/. Published August 2017. Accessed April 6, 2020.

108. Schoenmakers A, Mieras L, Budiawan T, van Brakel WH. The State of Affairs in Post-Exposure Leprosy Prevention: A Descriptive Meta-Analysis on Immuno- and Chemo-Prophylaxis. *Res Rep Trop Med*. 2020;Volume 11:97-117. doi:10.2147/rrtm.s190300

109. Ortuno-Gutierrez N, Younoussa A, Randrianantoandro A, et al. Protocol, rationale and design of PEOPLE (Post ExpOsure Prophylaxis for LEprosy in the Comoros and Madagascar): a cluster randomized trial on effectiveness of different modalities of implementation of post-exposure prophylaxis of leprosy contacts. *BMC Infect Dis*. 2019;19(1):1033. doi:10.1186/s12879-019-4649-0

110. Global Partnership for Zero Leprosy. Action Framework for Zero Leprosy. https://zeroleprosy.org/wp-content/uploads/2019/04/Action-Framework-PPT-slide-12-March-1.pdf. Published 2019. Accessed July 20, 2019.

111. Blok DJ. GPZL Reports on Research Priorities. *Lepr Rev*. 2019;90(3):237-289.

112. Wiltsey Stirman S, Kimberly J, Cook N, Calloway A, Castro F, Charns M. The sustainability of new programs and innovations: A review of the empirical literature and recommendations for future research. *Implement Sci*. 2012;7(1). doi:10.1186/1748-5908-7-17

For peer review only

For peer review only - http://bmjopen.bmj.com/site/about/guidelines.xhtml

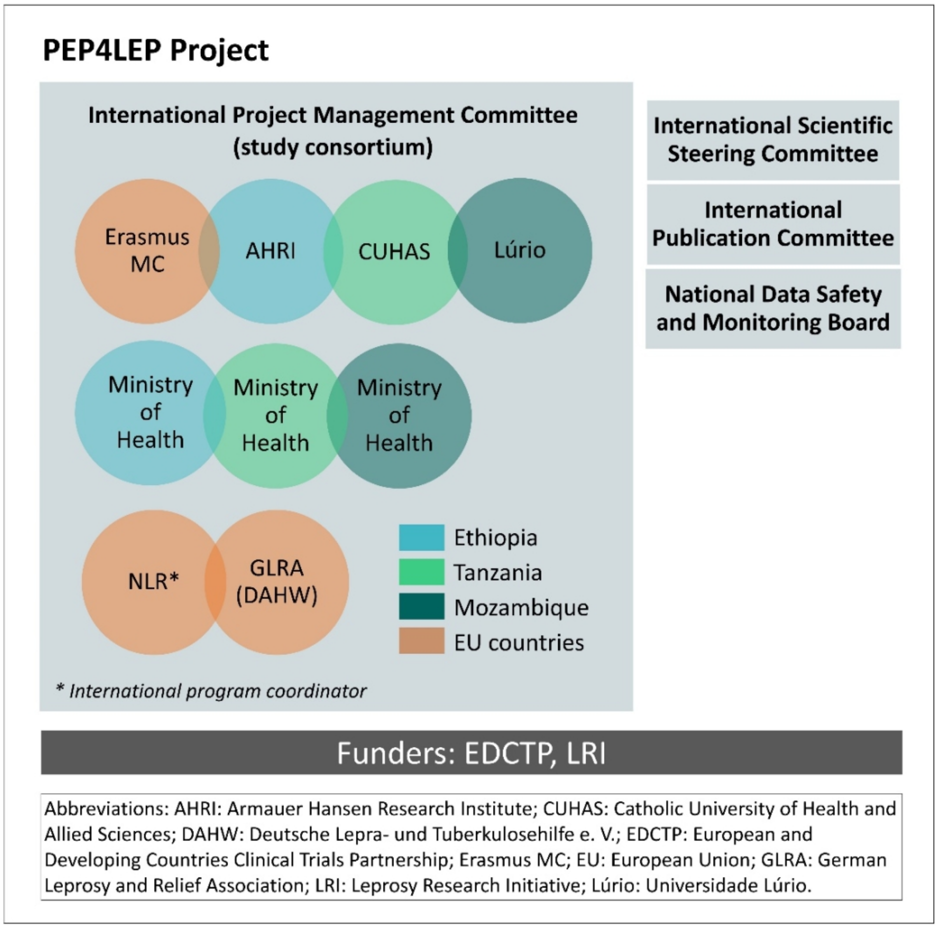

Figure 1. PEP4LEP Project organization chart

144x142mm (220 x 220 DPI)

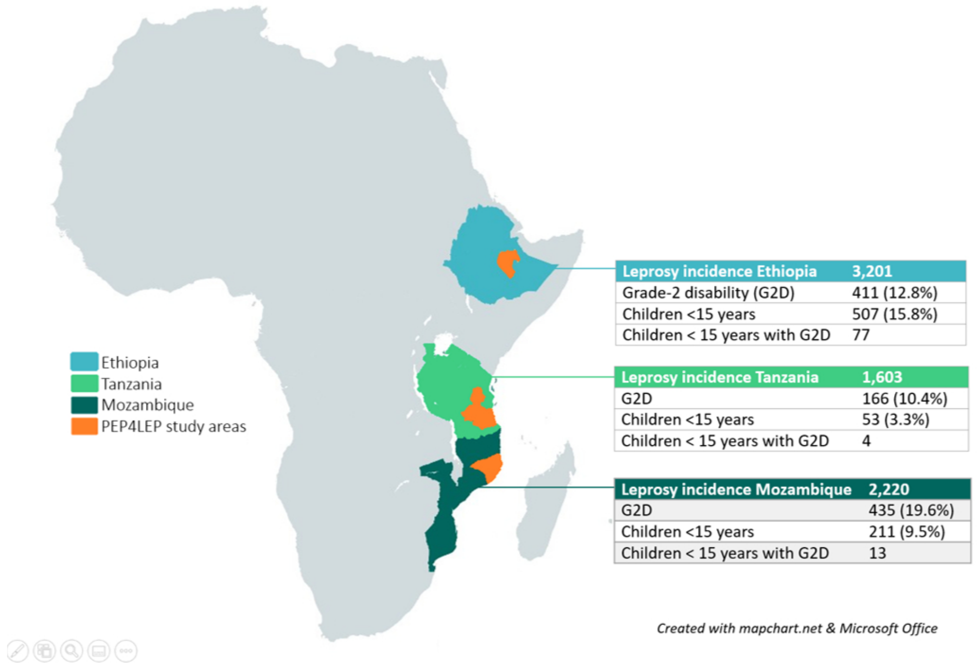

Figure 2. PEP4LEP countries' leprosy incidence (2019) according to the World Health Organization 2020

279x190mm (96 x 96 DPI)

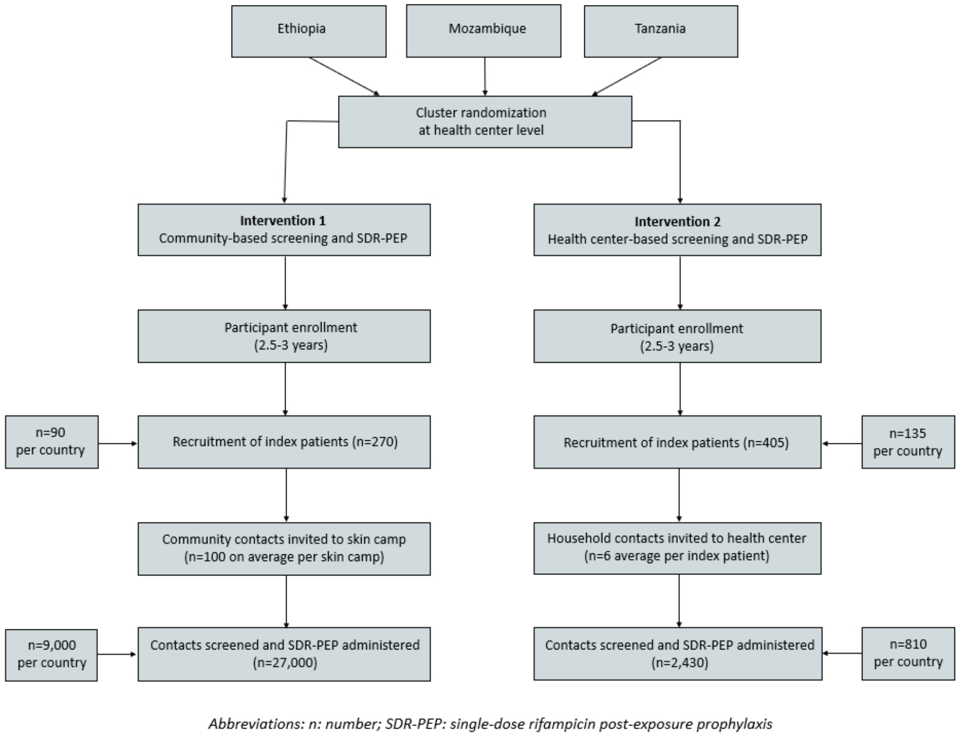

Figure 3. Flow of participants through the PEP4LEP study

224x174mm (110 x 110 DPI)

SPIRIT
STANDARD PROTOCOL ITEMS: RECOMMENDATIONS FOR INTERVENTIONAL TRIALS

SPIRIT 2013 Checklist: Recommended items to address in a clinical trial protocol and related documents*

Section/item	ItemNo	Description	BMJ Open
**Administrative information**			
Title	1	Descriptive title identifying the study design, population, interventions, and, if applicable, trial acronym	Page 1
Trial registration	2a	Trial identifier and registry name. If not yet registered, name of intended registry	Page 1
	2b	All items from the World Health Organization Trial Registration Data Set	N/A
Protocol version	3	Date and version identifier	Page 1, Trial registration
Funding	4	Sources and types of financial, material, and other support	Page 14, Funding
Roles and responsibilities	5a	Names, affiliations, and roles of protocol contributors	Page 1<br>Page 14, Authors' contributions
	5b	Name and contact information for the trial sponsor	Page 1
	5c	Role of study sponsor and funders, if any, in study design; collection, management, analysis, and interpretation of data; writing of the report; and the decision to submit the report for publication, including whether they will have ultimate authority over any of these activities	Page 11, Availability of data and materials<br>Page 3, Objectives (details on study consortium)<br>Page 14, Funding
	5d	Composition, roles, and responsibilities of the coordinating centre, steering committee, endpoint adjudication committee, data management team, and other individuals or groups overseeing the trial, if applicable (see Item 21a for data monitoring committee)	Page 11, Data collection and management<br>Page 12, Ethics
**Introduction**			

For peer review only

Background and rationale	6a	Description of research question and justification for undertaking the trial, including summary of relevant studies (published and unpublished) examining benefits and harms for each intervention	Page 3, Objectives
	6b	Explanation for choice of comparators	Page 7, Outcomes
Objectives	7	Specific objectives or hypotheses	Page 3, Objectives<br>Page 8, Table 3
Trial design	8	Description of trial design including type of trial (eg, parallel group, crossover, factorial, single group), allocation ratio, and framework (eg, superiority, equivalence, noninferiority, exploratory)	Page 5, Study Design<br>Page 6, Figure 3

Methods: Participants, interventions, and outcomes

Study setting	9	Description of study settings (eg, community clinic, academic hospital) and list of countries where data will be collected. Reference to where list of study sites can be obtained	Page 3, Study setting
Eligibility criteria	10	Inclusion and exclusion criteria for participants. If applicable, eligibility criteria for study centres and individuals who will perform the interventions (eg, surgeons, psychotherapists)	Page 4, Participants and eligibility criteria
Interventions	11a	Interventions for each group with sufficient detail to allow replication, including how and when they will be administered	Page 5, Community based skin camp intervention<br>Page 6, Health center-based intervention for household contacts
	11b	Criteria for discontinuing or modifying allocated interventions for a given trial participant (eg, drug dose change in response to harms, participant request, or improving/worsening disease)	Page 4, Participants and eligibility criteria<br>Page 4, Table 1
	11c	Strategies to improve adherence to intervention protocols, and any procedures for monitoring adherence (eg, drug tablet return, laboratory tests)	N/A
	11d	Relevant concomitant care and interventions that are permitted or prohibited during the trial	Page 4, Participants and eligibility criteria<br>Page 4, Table 1

For peer review only - http://bmjopen.bmj.com/site/about/guidelines.xhtml

Outcomes	12	Primary, secondary, and other outcomes, including the specific measurement variable (eg, systolic blood pressure), analysis metric (eg, change from baseline, final value, time to event), method of aggregation (eg, median, proportion), and time point for each outcome. Explanation of the clinical relevance of chosen efficacy and harm outcomes is strongly recommended	Page 7, Outcomes
Participant timeline	13	Time schedule of enrolment, interventions (including any run-ins and washouts), assessments, and visits for participants. A schematic diagram is highly recommended (see Figure)	Page 3, Study setting<br>Page 5, Study design<br>Page 6, Figure 3
Sample size	14	Estimated number of participants needed to achieve study objectives and how it was determined, including clinical and statistical assumptions supporting any sample size calculations	Page 10, Sample size
Recruitment	15	Strategies for achieving adequate participant enrolment to reach target sample size	Page 10, Sample size

Methods: Assignment of interventions (for controlled trials)

Allocation:

Sequence generation	16a	Method of generating the allocation sequence (eg, computer-generated random numbers), and list of any factors for stratification. To reduce predictability of a random sequence, details of any planned restriction (eg, blocking) should be provided in a separate document that is unavailable to those who enrol participants or assign interventions	Page 11, Data collection and management, paragraph 3
Allocation concealment mechanism	16b	Mechanism of implementing the allocation sequence (eg, central telephone; sequentially numbered, opaque, sealed envelopes), describing any steps to conceal the sequence until interventions are assigned	Page 10, Randomization

Implementation	16c	Who will generate the allocation sequence, who will enrol participants, and who will assign participants to interventions	Page 11, Data collection and management, paragraph 3
Blinding (masking)	17a	Who will be blinded after assignment to interventions (eg, trial participants, care providers, outcome assessors, data analysts), and how	N/A
	17b	If blinded, circumstances under which unblinding is permissible, and procedure for revealing a participant's allocated intervention during the trial	N/A
**Methods: Data collection, management, and analysis**			
Data collection methods	18a	Plans for assessment and collection of outcome, baseline, and other trial data, including any related processes to promote data quality (eg, duplicate measurements, training of assessors) and a description of study instruments (eg, questionnaires, laboratory tests) along with their reliability and validity, if known. Reference to where data collection forms can be found, if not in the protocol	Page 11, Data collection and management
	18b	Plans to promote participant retention and complete follow-up, including list of any outcome data to be collected for participants who discontinue or deviate from intervention protocols	Page 11, Data collection and management
Data management	19	Plans for data entry, coding, security, and storage, including any related processes to promote data quality (eg, double data entry; range checks for data values). Reference to where details of data management procedures can be found, if not in the protocol	Page 11, Data collection and management
Statistical methods	20a	Statistical methods for analysing primary and secondary outcomes. Reference to where other details of the statistical analysis plan can be found, if not in the protocol	Page 11, Data analysis
	20b	Methods for any additional analyses (eg, subgroup and adjusted analyses)	Page 11, Data analysis

	20c	Definition of analysis population relating to protocol non-adherence (eg, as randomised analysis), and any statistical methods to handle missing data (eg, multiple imputation)	Page 11, Data analysis
**Methods: Monitoring**			
Data monitoring	21a	Composition of data monitoring committee (DMC); summary of its role and reporting structure; statement of whether it is independent from the sponsor and competing interests; and reference to where further details about its charter can be found, if not in the protocol. Alternatively, an explanation of why a DMC is not needed	Page 12, Ethics
	21b	Description of any interim analyses and stopping guidelines, including who will have access to these interim results and make the final decision to terminate the trial	N/A
Harms	22	Plans for collecting, assessing, reporting, and managing solicited and spontaneously reported adverse events and other unintended effects of trial interventions or trial conduct	Page 12, Ethics
Auditing	23	Frequency and procedures for auditing trial conduct, if any, and whether the process will be independent from investigators and the sponsor	Page 12, Ethics
**Ethics and dissemination**			
Research ethics approval	24	Plans for seeking research ethics committee/institutional review board (REC/IRB) approval	Page 12, Ethics
Protocol amendments	25	Plans for communicating important protocol modifications (eg, changes to eligibility criteria, outcomes, analyses) to relevant parties (eg, investigators, REC/IRBs, trial participants, trial registries, journals, regulators)	Page 12, Ethics
Consent or assent	26a	Who will obtain informed consent or assent from potential trial participants or authorised surrogates, and how (see Item 32)	Page 12, Ethics

only

For peer review only - http://bmjopen.bmj.com/site/about/guidelines.xhtml

	26b	Additional consent provisions for collection and use of participant data and biological specimens in ancillary studies, if applicable	N/A
Confidentiality	27	How personal information about potential and enrolled participants will be collected, shared, and maintained in order to protect confidentiality before, during, and after the trial	Page 11, Data collection and management, paragraph 3
Declaration of interests	28	Financial and other competing interests for principal investigators for the overall trial and each study site	Page 14, Competing interests
Access to data	29	Statement of who will have access to the final trial dataset, and disclosure of contractual agreements that limit such access for investigators	Page 11, Availability of data and materials
Ancillary and post-trial care	30	Provisions, if any, for ancillary and post-trial care, and for compensation to those who suffer harm from trial participation	N/A
Dissemination policy	31a	Plans for investigators and sponsor to communicate trial results to participants, healthcare professionals, the public, and other relevant groups (eg, via publication, reporting in results databases, or other data sharing arrangements), including any publication restrictions	Page 11, Availability of data and materials
	31b	Authorship eligibility guidelines and any intended use of professional writers	N/A
	31c	Plans, if any, for granting public access to the full protocol, participant-level dataset, and statistical code	Page 11, Availability of data and materials
**Appendices**			
Informed consent materials	32	Model consent form and other related documentation given to participants and authorised surrogates	Added to supplementary materials of submission
Biological specimens	33	Plans for collection, laboratory evaluation, and storage of biological specimens for genetic or molecular analysis in the current trial and for future use in ancillary studies, if applicable	N/A

*It is strongly recommended that this checklist be read in conjunction with the SPIRIT 2013 Explanation & Elaboration for important clarification on the items. Amendments to the

For peer review only - http://bmjopen.bmj.com/site/about/guidelines.xhtml

protocol should be tracked and dated. The SPIRIT checklist is copyrighted by the SPIRIT Group under the Creative Commons "Attribution-NonCommercial-NoDerivs 3.0 Unported" license.

For peer review only

For peer review only - http://bmjopen.bmj.com/site/about/guidelines.xhtml
